# Defining variant-resistant epitopes targeted by SARS-CoV-2 antibodies: A global consortium study

**DOI:** 10.1126/science.abh2315

**Published:** 2021-09-23

**Authors:** Kathryn M. Hastie, Haoyang Li, Daniel Bedinger, Sharon L. Schendel, S. Moses Dennison, Kan Li, Vamseedhar Rayaprolu, Xiaoying Yu, Colin Mann, Michelle Zandonatti, Ruben Diaz Avalos, Dawid Zyla, Tierra Buck, Sean Hui, Kelly Shaffer, Chitra Hariharan, Jieyun Yin, Eduardo Olmedillas, Adrian Enriquez, Diptiben Parekh, Milite Abraha, Elizabeth Feeney, Gillian Q. Horn, CoVIC-DB team, Yoann Aldon, Hanif Ali, Sanja Aracic, Ronald R. Cobb, Ross S. Federman, Joseph M. Fernandez, Jacob Glanville, Robin Green, Gevorg Grigoryan, Ana G. Lujan Hernandez, David D. Ho, Kuan-Ying A. Huang, John Ingraham, Weidong Jiang, Paul Kellam, Cheolmin Kim, Minsoo Kim, Hyeong Mi Kim, Chao Kong, Shelly J. Krebs, Fei Lan, Guojun Lang, Sooyoung Lee, Cheuk Lun Leung, Junli Liu, Yanan Lu, Anna MacCamy, Andrew T. McGuire, Anne L. Palser, Terence H. Rabbitts, Zahra Rikhtegaran Tehrani, Mohammad M. Sajadi, Rogier W. Sanders, Aaron K. Sato, Liang Schweizer, Jimin Seo, Bingqing Shen, Jonne L. Snitselaar, Leonidas Stamatatos, Yongcong Tan, Milan T. Tomic, Marit J. van Gils, Sawsan Youssef, Jian Yu, Tom Z. Yuan, Qian Zhang, Bjoern Peters, Georgia D. Tomaras, Timothy Germann, Erica Ollmann Saphire

**Affiliations:** 1 Center for Infectious Disease and Vaccine Research, La Jolla Institute for Immunology, 9420 Athena Circle, La Jolla, CA 92037, USA.; 2 Carterra, 825 N. 300 W. Ste C309, Salt Lake City, UT 84103, USA.; 3 Center for Human Systems Immunology, Departments of Surgery, Immunology, and Molecular Genetics and Microbiology and Duke Human Vaccine Institute, Duke University, Durham, NC 27701, USA.; 4 Department of Medical Microbiology and Infection Prevention, Amsterdam University Medical Centers, Location AMC, University of Amsterdam, Amsterdam Infection & Immunity Institute, 1105 AZ Amsterdam, Netherlands.; 5 Quadrucept Bio, Ltd., Cambridge CB23 6DW, UK.; 6 Myrio Therapeutics Pty, Ltd., 1 Dalmore Drive, Scoresby, VIC 3179, Australia.; 7 National Resilience, Inc., 13200 NW Nano Ct., Alachua, FL 32615, USA.; 8 Generate Biomedicines, Inc., 26 Landsdowne Street, Cambridge, MA 02139, USA.; 9 Activemotif, Inc., 1914 Palomar Oaks Way, Suite 150, Carlsbad, CA 92008, USA.; 10 Centivax, Inc., 201 Gateway Blvd., Floor 1, South San Francisco, CA 94080, USA.; 11 Twist Bioscience, 681 Gateway Blvd., South San Francisco, CA 94080, USA.; 12 Aaron Diamond AIDS Research Center, Columbia University Vagelos College of Physicians and Surgeons, 701 West 168th St., HHSC 1102, New York, NY 10032, USA.; 13 Division of Pediatric Infectious Diseases, Department of Pediatrics, Chang Gung Memorial Hospital and Research Center for Emerging Viral Infections, Chang Gung University, Taoyuan, Taiwan.; 14 Shanghai Henlius Biotech, Inc., 9/F, Innov Tower, Zone A, no. 1801 Hongmei Road, Xuhui District, Shanghai, China.; 15 Kymab, Ltd., The Bennet Building, Babraham Research Campus, Cambridge CB22 3AT, UK.; 16 Department of Infectious Disease, Imperial College, London SW7 2AZ, UK.; 17 Celltrion, Inc., Department of Research and Development, 23 Academy-ro Yeonsu-gu Incheon, Republic of Korea.; 18 Sanyou Biopharmaceuticals Co., Ltd., no. 188 Xinjunhuan Road, Building 6B-C, 3rd Floor, Minhang District, Shanghai 201114, China.; 19 Emerging Infectious Diseases Branch, Walter Reed Army Institute of Research, Silver Spring, MD 20910, USA.; 20 Shanghai Key Laboratory of Medical Epigenetics, International Laboratory of Medical Epigenetics and Metabolism, Ministry of Science and Technology, Institutes of Biomedical Sciences, Fudan University, Shanghai, China.; 21 AbCipher Biotechnology, 188 Xinjun Ring Road, Building 2, 4th Floor, Minhang District, Shanghai 201114, China.; 22 Fred Hutchinson Cancer Research Center, Vaccines and Infectious Diseases Division, Seattle, WA, USA.; 23 Institute of Cancer Research, Centre for Cancer Drug Discovery, London SM2 5NG, UK.; 24 Division of Clinical Care and Research, Institute of Human Virology, University of Maryland, Baltimore, MD 21201, USA.; 25 HiFiBiO, Inc., 237 Putnam Avenue, Cambridge, MA 02139, USA.; 26 National Resilience, Inc., 2061 Challenger Dr., Alameda, CA 94501, USA.; 27 Department of Medicine, University of California, San Diego, La Jolla, CA 92037, USA.

## Abstract

Antibody-based therapeutics and vaccines are essential to combat COVID-19 morbidity and mortality after severe acute respiratory syndrome coronavirus 2 (SARS-CoV-2) infection. Multiple mutations in SARS-CoV-2 that could impair antibody defenses propagated in human-to-human transmission and spillover or spillback events between humans and animals. To develop prevention and therapeutic strategies, we formed an international consortium to map the epitope landscape on the SARS-CoV-2 spike protein, defining and structurally illustrating seven receptor binding domain (RBD)–directed antibody communities with distinct footprints and competition profiles. Pseudovirion-based neutralization assays reveal spike mutations, individually and clustered together in variants, that affect antibody function among the communities. Key classes of RBD-targeted antibodies maintain neutralization activity against these emerging SARS-CoV-2 variants. These results provide a framework for selecting antibody treatment cocktails and understanding how viral variants might affect antibody therapeutic efficacy.

## Community of antibodies against COVID-19

The severe acute respiratory syndrome coronavirus 2 spike protein is the basis of many vaccines and is a primary target of neutralizing antibodies after COVID-19 infection. The Coronavirus Immunotherapeutic Consortium (CoVIC), comprising 56 partners across the world, has analyzed a panel of 269 monoclonal antibodies (mAbs) and, on the basis of competition profiles, sorted 186 mAbs that target the receptor binding domain into seven communities. Hastie *et al*. went on to structurally analyze representative antibody binding and used pseudovirus neutralization assays to study the effect of spike mutations on antibody function, including the combinations of mutations found in certain variants of concern. These results are important to guide both treatment and prevention efforts. —VV

## Teaser

SARS-CoV-2 spike antibodies in three distinct epitope communities retain activity against SARS-CoV-2 variants.

Cell entry of severe acute respiratory syndrome coronavirus 2 (SARS-CoV-2) is mediated by its surface glycoprotein, spike. The S1 subunit of spike contains the N-terminal domain (NTD) and the receptor binding domain (RBD), which mediates recognition of the host cell receptor angiotensin-converting enzyme 2 (ACE2). The S2 subunit drives fusion between virus and host cell membranes. Spike, particularly the S1 subunit, is the primary target of neutralizing antibodies against SARS-CoV-2 (*1*).

Since SARS-CoV-2 first emerged, recurrent mutations in spike arose during both human-to-human transmission (*2*–*4*) and spillover or spillback events between humans and animals (*5*–*8*). Distinct variants of concern (VOCs) or variants of interest (VOIs)—including those first identified in the UK (Alpha, B.1.1.7), South Africa (Beta, B.1.351), Brazil (Gamma, P.1), India (Delta, B.1.617.2), and California (Epsilon, B.1.429)—carry several mutations associated with enhancement of human-to-human transmission (*9*). In particular, the receptor binding motif (RBM) mutations K417, L452, E484, and N501 affect ACE2-spike interactions (*10*). Variations at positions N439 and S477 are frequently detected in patient samples (*3*, *11*, *12*), whereas others such as V367F, Y453F, and F486L are associated with cross-species transmission (*6*, *8*). The NTD is also highly mutable and is especially prone to deletions: ∆HV69-70 and ∆Y144 are both seen in B.1.1.7 and ∆HV69-70 is in the mink-associated Cluster V (*6*). ∆LAL242-244 appears in B.1.351, and ∆FR157-158 is found in B.1.617.2 (*9*). The NTD point mutations S13I and W152C alter disulfide bonding and conformation of the B.1.429 NTD (*13*) (fig. S1).

SARS-CoV-2 will continue to evolve. By understanding antibody footprints and the distinct ways by which antibodies target spike, we may deduce optimal combinations of monoclonal antibodies (mAbs) to prevent and treat infection by emerging variants and to minimize the risk of viral escape. We can also gauge the susceptibility of mapped antibodies to new mutations and predict whether newly identified mAbs might also be susceptible to viral escape. Thus, we sought to define functionally important groups in an array of therapeutic candidates and to dissect how key mutations, both individually and combined as in VOCs, affect antibody-mediated neutralization in a pseudovirus neutralization assay.

The Coronavirus Immunotherapeutic Consortium (CoVIC) was formed to analyze candidate antibody therapeutics side by side in standardized assays (*14*) and now includes more than 350 mAbs directed against the SARS-CoV-2 spike protein from 56 different partners across four continents (*15*). The panel includes antibodies derived from COVID-19 survivors, phage display, naïve libraries, in silico methods, and other strategies—each elicited, evaluated, and selected using distinct criteria. The panel thus represents a broader and deeper array of antibodies from which both fundamental information and therapeutic cocktails can be derived. With the goals of FAIR (findable, accessible, interoperable, reusable) data analysis and management as well as inclusion of otherwise inaccessible clinical candidates, candidate antibody therapeutics were blinded and tested in multiple in vitro and in vivo assays with comparative data uploaded into a publicly accessible database (covic.lji.org).

**Fig. 1.**
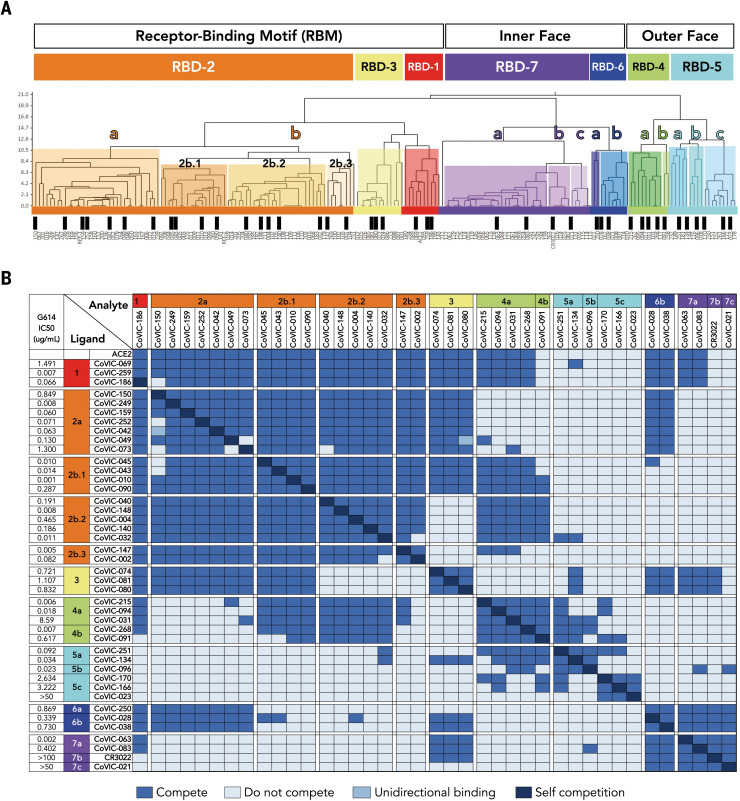
The antigenic landscape of the SARS-CoV-2 RBD can be divided into seven binding communities. (**A**) HT-SPR was used to determine the competitive relationship between 186 RBD-directed mAbs. The dataset was analyzed by Carterra Epitope software to sort competition profiles of clones into related clusters, which are represented as regions of the dendrogram with shared color. The RBD epitope landscape can be broadly divided into seven communities containing mAbs that bind the RBM (RBD-1 through RBD-3), the outer face of the RBD (RBD-4 and RBD-5), or the inner face of the RBD (RBD-6 and RBD-7). Communities can be further divided into smaller clusters (e.g., RBD-2a and RBD-2b) and bins (e.g., RBD-2b.1, RBD-2b.2, and RBD-2b.3) on the basis of their discrete competition with other clusters and/or their ability to compete with ACE2 for spike binding. Black bars indicate single clones that were used in further analyses. Table S1 lists additional metrics (i.e., ACE2 blocking, kinetic analyses, and germline information) for the indicated mAbs; detailed information for the entire CoVIC panel can be found at covic.lji.org. (**B**) Binary heatmap matrix demonstrating the competition profile for the finer clusters and bins for the subset of single clones indicated by black bars in (A). The matrix here contains representative examples of each epitope community. RBD-2 can be divided into clusters a and b, which have varying ability to compete with mAbs in RBD-4 (e.g., RBD-2a mAbs do not compete, whereas most RBD-2b mAbs do). Cluster RBD-2b can be divided into three smaller bins that vary in their competition with both RBD-3 and RBD-4 mAbs: Those in 2b.1, but not 2b.2 or 2b.3, compete with RBD-3 mAbs, whereas mAbs in 2b.1 and 2b.2, but not 2b.3, compete with RBD-4 mAbs. RBD-4 contains mAbs that do (RBD-4a) and do not (RBD-4b) compete with ACE2. RBD-5 and RBD-7 have clusters of mAbs with lower neutralizing potency (i.e., RBD-5c, RBD-7b, and RBD-7c) relative to the other cluster in the same community (i.e., RBD-5a, RBD-5b, and RBD-7a). Rows and columns indicate the immobilized mAb and injected analyte mAb, respectively. Table S2 shows the complete matrix for competition among all 186 mAbs.

We first measured the affinity of 269 CoVIC mAbs for D614-Hexapro spike ectodomain trimers and monomeric RBD and NTD and the ability of each of these mAbs to block ACE2-RBD binding (figs. S2 to S5, table S1, and covic.lji.org). The panel, formed by candidates for therapeutic use, includes NTD- or S2-directed antibodies but is dominated by those targeting the RBD. In contrast to previous studies that classified mAbs using germline or structural information (*10*, *16*), the 186 RBD-reactive mAbs of CoVIC analyzed in this study were instead distinguished by a competition profile created by high-throughput surface plasmon resonance (HT-SPR). RBD-directed antibodies can be sorted into seven core “communities” (Fig. 1, fig. S6A, and table S2) that are broadly defined by the competition profiles of each mAb relative to the others. Communities can be further divided into finer clusters and bins on the basis of their discrete competition with other clusters and/or their ability to compete with ACE2 (Fig. 1 and tables S1 and S2).

To understand the position of each community relative to the others, we next mapped the footprints by negative-stain electron microscopy (NS-EM) for 25 example RBD-reactive mAbs chosen to span the range of communities and key clusters (table S3). To have a relatively agnostic view of antibody interactions with spike, mAbs were not chosen on the basis of germline origin, CDR (complementarity-determining region) feature or length, neutralization potency, or particular antibody origin (e.g., human, mouse, in silico) or format (e.g., IgG, scFv-Fc, VHH-Fc, multivalent).

In parallel, we measured the neutralization activity of 41 RBD-directed mAbs (chosen to span the range of communities and key clusters) as well as a human ACE2-Fc fusion therapeutic candidate (CoVIC-069). Neutralization was measured against pseudoviruses that display the spike protein bearing (i) the globally dominant G614 variation, (ii) 15 single point mutations or deletions represented in circulating strains, (iii) constellations of mutations found in four VOCs [B.1.1.1 (Alpha), B.1.351 (Beta), P.1 (Gamma), and B.1.617.2 (Delta)] and one VOI [B.1.429 (Epsilon)], and (iv) two pseudovariants carrying four mutations (termed 4xM; containing G261D, Y453F, F486L, and N501T) or five mutations (termed 5xM; carrying the 4xM mutations plus V367F) identified in human–mink spillover events (fig. S1).

**Fig. 2.**
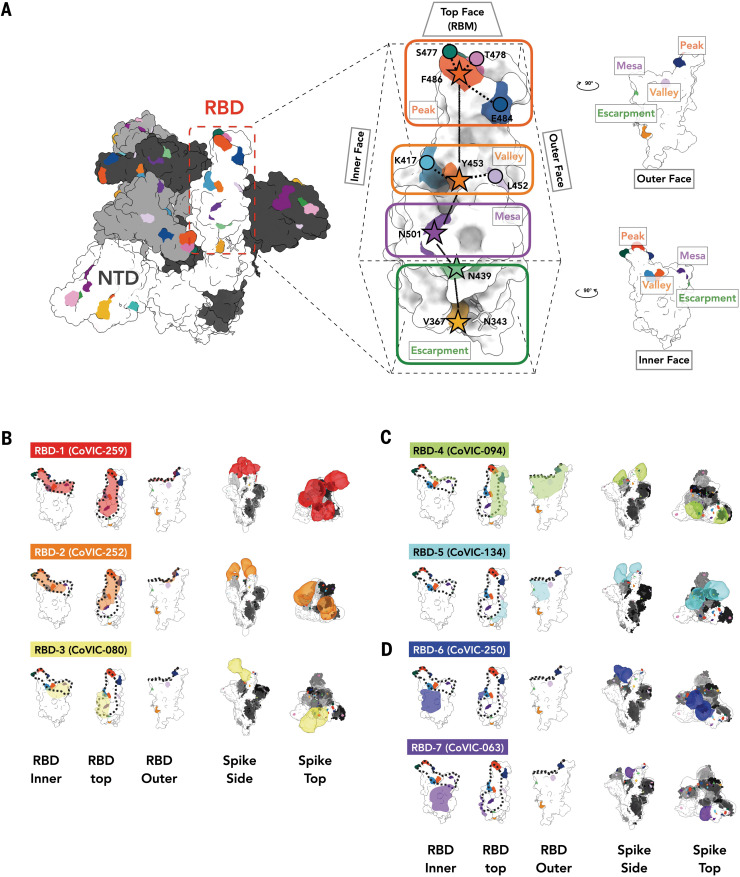
NS-EM analysis of representatives from each RBD-directed community. (**A**) Location of important emerging mutations in a RBD. The spike trimer [adapted from PDB ID 7A94 (*39*)] viewed from the top with one “up” RBD is shown; individual spike monomers are colored white, gray, and black. The RBM can be topologically divided into three subsections: the “peak” that includes residues F486, S477, T478, and E484; the “valley” including residues Y453, K417, and L452; and the “mesa” with residue N501. Stars indicate residues on the central axis of the RBD. The “outer face” [exposed in the RBD down (closed) conformation] and “inner face” [buried inside the trimer in the RBD down (closed) conformation] define the lateral faces of the RBD and the “escarpment” (contains residues V367 and N439 and glycan 343). (**B** to **D**) NS-EM footprint of a representative antibody from each community mapped onto an RBD monomer. The colored shading corresponds to the community colors in Fig. 1. The ACE2 binding site is outlined with a dotted line. Side and top views of spike trimers show the Fab approach angle and binding stoichiometry for each representative. Table S3 shows NS-EM data for all of the 29 RBD-directed mAbs that we analyzed.

The mAbs in RBD-1 through RBD-3 target the RBM, compete with ACE2, and generally require the RBD to be in the “up” conformation for binding (footprints defined in Fig. 2B, table S3, and covic.lji.org). Community RBD-1 contains hACE2-derived molecules and IgGs (e.g., CoVIC-259, EMD-24335) that largely overlap with the RBM (Fig. 2B, fig. S6B, and table S3). The footprint for RBD-2 mAbs is shifted from the center of the ACE2 binding site toward the peak of the RBM (Fig. 2B, fig. S6B, and table S3). RBD-2 is the largest community and can be divided further into clusters and then bins on the basis of competition with other communities (Fig. 1). Cluster 2a antibodies (e.g., CoVIC-252, EMD-24339) bind toward the inner face of the RBD and its binding area overlaps highly with that of the therapeutic antibody REGN-10933 (*17*). Antibodies in 2b.1 [e.g., CoVIC-010, EMD-24343; similar to antibody COVA2-39 (*18*)] and 2b.2 [e.g., CoVIC-140, EMD-24383; similar to antibody C144 (*16*)] bind toward the outer face of the RBD, and mAbs in bin 2b.3 [e.g., CoVIC-002, EMD-24345; similar to antibody S2E12 (*19*)] bind to the peak of the RBD (Fig. 2B, fig. S7, and table S3). Lastly, RBD-3 mAbs bind down from the center of the ACE2 binding site toward the RBD “mesa” [Fig. 2B and table S3; e.g., CoVIC-080, EMD-24346; similar to antibody ADI-56046 (*20*)].

To simulate the authentic interactions between antibodies and spike, intact IgGs were used for NS-EM structural analysis whenever possible. RBD-1 IgGs tend to fully occupy all three RBDs on one spike and often cross-link two spike trimers, whereas most RBD-2 IgGs tend to bind bivalently to a single spike trimer (figs. S8, A and B, and S9 and table S3). RBD-3 IgGs can cross-link spikes, and bivalent binding was also observed in some cases (table S3 and fig. S9).

**Fig. 3.**
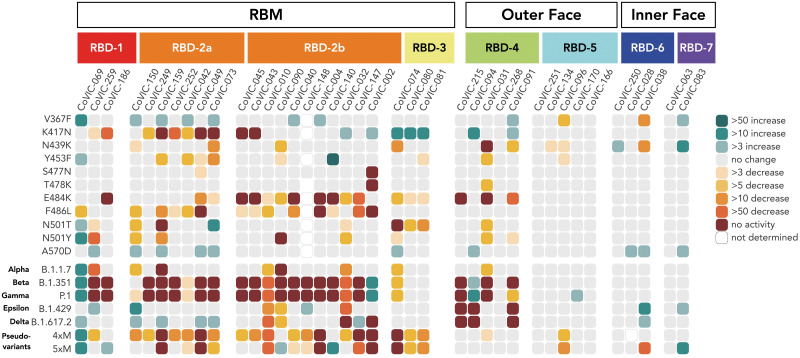
RBD-5, -6, and -7 antibodies retain neutralization activity against pseudovirus bearing mutations singly or together in VOCs. Fold-change differences in potency for 38 RBD-directed antibodies and an ACE2-Fc fusion (CoVIC-069) are shown in a heatmap. In addition to VOCs, we also examined two pseudoviruses bearing clusters of mink-associated mutations: 4xM (G261D, Y453F, F486L, and N501T) and 5xM (G261D, Y453F, F486L, N501T, and V367F). Fig. S1 lists mutations represented in each variant. Fig. S10 shows neutralization curves for each virus–variant pair, and table S4 lists fold-change values corresponding to the heatmap. Single-letter abbreviations for amino acid residues: A, Ala; C, Cys; D, Asp; E, Glu; F, Phe; G, Gly; H, His; I, Ile; K, Lys; L, Leu; M, Met; N, Asn; P, Pro; Q, Gln; R, Arg; S, Ser; T, Thr; V, Val; W, Trp; and Y, Tyr.

General epitope position, particularly that of RBM epitopes, is strongly associated with the propensity of particular spike mutations to escape antibody-mediated neutralization (Fig. 3, fig. S10, and table S4). Neutralization by RBD-2a antibodies is heavily affected by the K417N mutation but rarely by the E484K mutation; those in RBD-2b are affected by the E484K mutation but less so by K417N. Similarly, RBD-2a antibodies are resistant to the L452R mutation found in B.1.429 (Epsilon) and B.1.617.2 (Delta), whereas only some RBD-2b antibodies are sensitive to this mutation. Meanwhile, mAbs in RBD-3 are affected by both N501T/Y and E484K mutations (Fig. 3, figs. S10 and S11, and table S4). In contrast to RBD-2 and RBD-3, the susceptibility of neutralization activity of antibodies in RBD-1 to particular mutations is more variable (Figs. 2B and 3 and tables S3 and S4).

Regardless of the effect of particular single point mutations, nearly every RBD-1 or RBD-2 mAb analyzed showed additive decreases in potency against pseudovirus carrying constellations of multiple mutations in the RBM (Fig. 3, fig. S10, and table S4). For B.1.351 and P.1, almost all of the RBD-1 and RBD-2 antibodies that we analyzed suffer a complete loss of neutralization activity. For example, CoVIC-249 and CoVIC-010 show moderate or no change in IC_50_ (half-maximal inhibitory concentration) against the single point mutations K417N, E484K, and N501Y, but CoVIC-249 loses all neutralization activity and CoVIC-010 potency falls by 1000-fold against B.1.351 (Beta) and P.1 (Gamma), which contain all three mutations. Many RBD-2 antibodies also lose activity against the 4xM mink pseudovariant that carries Y453F, F486L, and N501T mutations (Fig. 3, figs. S1C and S10, and table S4).

By contrast, most RBD-1 and RBD-2 antibodies retain neutralization activity against B.1.1.7 (Alpha), B.1.429 (Epsilon), and B.1.617.2 (Delta) variants, which each contain only one or two RBM-located mutations (N501Y, L452R, or T478K/L452R respectively). Curiously, the V367F mutation identified in mink populations enhances neutralization by some RBD-2 mAbs, and in some cases, this mutation can offset decreases in potency resulting from other single point mutations. For example, CoVIC-040 has 14- and 8-fold decreases in potency against the F486L mutation and the F486L-containing 4xM mink pseudovariant, respectively, but only a 4-fold decrease against the 5xM mink pseudovariant, which contains V367F in addition to the four mutations present in 4xM (Fig. 3, fig. S10, and table S4). V367 is adjacent to an N-linked glycan at position 343, which was recently implicated in providing a gating mechanism for the RBD (*21*). Substitution of valine with phenylalanine could alter the local environment of the N343 glycan moieties and enable the RBD to adopt a conformation more amenable to antibody interaction.

Antibodies in communities RBD-4 and RBD-5 bind to the outer face of the RBD and, like the previously defined class 2 and class 3 mAbs (*16*), can do so in either the “up” or “down” RBD conformation without steric hindrance (Fig. 2C, figs. S6 and S12, and table S3). The footprints of these groups largely overlap, but RBD-4 mAbs bind toward the outer edge of the RBM and can block ACE2 [e.g., CoVIC-094, EMD-24350; similar to antibody C002 (*16*)], whereas RBD-5 mAbs bind away from the RBM, toward the S309 site, and block ACE2 weakly [e.g., CoVIC-134, EMD-24384; similar to antibody REGN-10987 (*17*)] (Figs. 1B and 2C, figs. S5 and S6B, and tables S1 to S3) (*10*). Some RBD-4 and RBD-5 IgGs can cross-link spike trimers in solution (fig. S8C and table S3).

Notably, in accordance with the five RBD-5 IgGs we imaged, only those IgGs that show spike-cross linking tendency have potent neutralizing activity (Fig. 1B, fig. S13, and table S3). A recent cryo–electron tomography study showed that native spike trimers on the SARS-CoV-2 virion surface tilt at variable degrees relative to the viral envelope (*22*). This finding provides a possibility for IgG-mediated spike cross-linking on virions and may contribute to the mechanism of neutralization of the RBD-5 mAbs in the absence of ACE2 blocking (fig. S8D).

Most RBD-4 mAbs are affected by E484K and/or L452R mutations (represented in the B.1.429 variant) (Fig. 3, fig. S10, and table S4), and some are affected by the N439K mutation, which is highly represented in sequences worldwide (*3*). RBD-5 mAbs, however, show broad resistance to nearly all mutations analyzed, with only two mAbs in this group showing moderate decreases in potency against V367F and N439K (Fig. 3, fig. S10, and table S4).

RBD-6 (e.g., CoVIC-250, EMD-24352) and RBD-7 (e.g., CoVIC-063, EMD-24353) antibodies bind to the inner face of the RBD and access a previously described cryptic epitope (*23*, *24*) (Fig. 2D, fig. S6B, and table S3). Similar to the binding of class 4 antibodies (*16*), binding of spike by RBD-6 and RBD-7 antibodies requires two RBDs to be in the up configuration (fig. S12). The representative IgGs in RBD-6 and RBD-7 each show stronger propensities to cross-link spike trimers than do RBM-directed antibodies (fig. S9 and table S3). RBD-6 and RBD-7 antibodies primarily vary in their competition with RBD-2a antibodies: The downward shift of the RBD-7 footprint on the inner face of the RBD relative to the RBD-6 footprint would allow simultaneous binding of RBD-2a antibodies with RBD-7 antibodies but not RBD-6 antibodies (Figs. 1B and 2D, fig. S6B, and table S2). This cryptic site targeted by RBD-6 and RBD-7 antibodies is also recognized by antibodies COVA1-16 (*23*) and CR3022 (*24*). Here, strategies of site recognition are further subdivided by competition subgroups—information that is useful for interpreting differences and antibody behavior and strategies for cocktail selection.

All RBD-6 and RBD-7a antibodies block ACE2, but antibodies in RBD-7b and RBD-7c do not (Fig. 1B and tables S1 to S3). The representatives from the RBD-7b and RBD-7c clusters (CR3022 and CoVIC-021, respectively) demonstrate poor neutralization of pseudoviruses in our assay. The characteristic difference in neutralization behavior between 7a and 7b or 7c suggests that at this cryptic epitope, competition with ACE2 is a determinant of neutralization (Fig. 1B and table S4) (*25*). Notably, owing to their location away from the RBM, RBD-6 and RBD-7 antibodies are resistant to the mutations and variants analyzed (Fig. 3, fig. S10, and table S4).

**Fig. 4.**
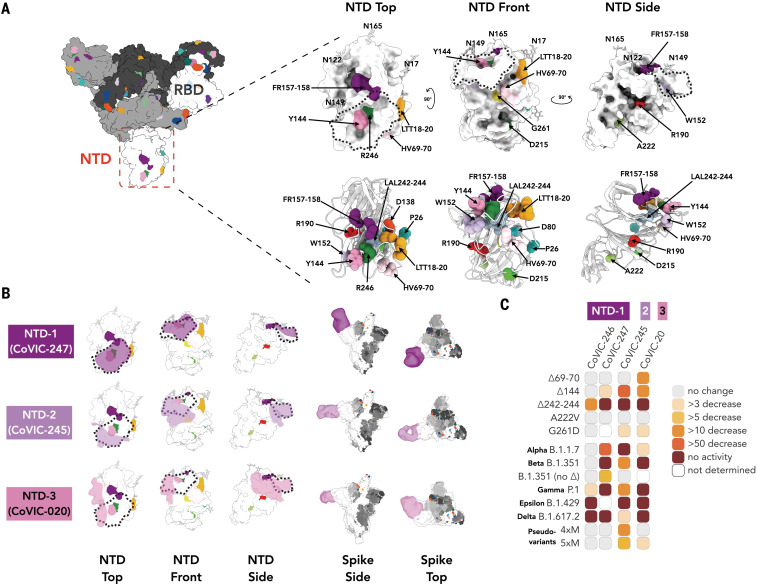
NS-EM and neutralization analysis of mAbs targeting the NTD. (**A**) Surface and cartoon [adapted from PDB ID 7A94 (*39*)] representation of the spike NTD. The residue positions of mutations and deletions in circulating VOCs are indicated in three views of the NTD. Fig. S1 lists mutations represented in each variant. (**B**) Footprints for three NTD-targeted antibodies, with the NTD “supersite” (*26*) outlined with a dashed line. The NTD-directed antibodies shown here define the approximate boundaries of the neutralizing epitope landscape. Additional NS-EM data are in table S3. (**C**) Fold-change in potency of pseudovirus neutralization experiments for each antibody–variant pair.

Previous reports identified a “supersite” as the primary target for neutralizing antibodies directed against the NTD (*26*). In addition to RBD-directed antibodies, we also analyzed four CoVIC NTD-directed antibodies by NS-EM and in neutralization assays. Together, these four antibodies (grouped as NTD-1 through NTD-3), encompass the approximate boundaries of the supersite. The two NTD-1 antibodies bind from the top side of the NTD to cover the NTD N terminus and residue Y144 [Fig. 4; e.g., CoVIC-247, EMD-24355 (table S3)]. The NTD-1 epitope overlaps with that of mAb 4A8 (*27*) and other supersite binders (*28*, *29*). The NTD-2 antibody (CoVIC-245, EMD-24360) approaches from the front side of the NTD and contacts Y144 as well as residues H69, V70, W152, and G261, all of which are deleted or substituted in emerging variants (Fig. 4 and fig. S1). The NTD-2 footprint is similar to the footprint of antibodies in the antigenic site V group (*26*). The NTD-3 mAb (CoVIC-020, EMD-24356) binds to the left side of the NTD, proximal to the RBD of the adjacent monomer and in contact with residue W152 (Fig. 4B). The NTD-3 mAb represents a novel epitope and binding location of an anti-NTD antibody.

Unlike the RBD-directed antibodies, for which neutralization escape is strongly associated with antibody footprint, the NTD-directed antibodies are conformationally sensitive and affected by mutations outside of the discrete footprint. This finding is consistent with that for antibodies elicited by vaccines (*30*). Each of the four NTD mAbs analyzed exhibits a decreased or total loss of neutralization capacity for one or more of the NTD-located deletions (∆69/70, ∆Y144, ∆157-158, and ∆242-244) found in circulating VOCs, regardless of their binding location on the NTD (Fig. 4C, fig. S10, and table S4). All NTD mAbs were affected by P.1 (Gamma), which lacks deletions and instead has several point mutations in the NTD. For B.1.429 (Epsilon), altered disulfide bonding in the NTD arising from the S13I and W152C mutations (*13*) also abrogated mAb-mediated neutralization. Our results indicate that NTD mutations decrease not only neutralization potency but also the total fraction of virus neutralized (fig. S10).

Several therapeutic antibody cocktails that include pairs of different mAbs against spike are currently under investigation for postexposure treatment of COVID-19 (*16*, *17*, *31*, *32*). However, the potency of some antibodies in these cocktails is compromised by emerging SARS-CoV-2 variants (*33*, *34*). Meanwhile, exposure of virus to monoclonal or polyclonal antibodies can promote antibody-resistant mutations in spike (*34*–*37*). Notably, SARS-CoV-2 variants that share critical mutations with B.1.1.7 (Alpha) were isolated from an immunocompromised COVID-19 patient who received three rounds of convalescent plasma treatment, indicating that even a polyclonal therapeutic can drive evolution of resistant virus strains in unresolved infections (*38*).

Potency, variant resistance, and the ability to cobind are important considerations when selecting antibodies for therapeutic cocktails. The analysis of the 186 RBD-directed mAbs presented here—each donated by different groups around the world and selected in different ways—describes discrete antibody communities and functionally relevant subclusters and/or bins. This analysis provides a competition grid and a framework for cocktail selection. Notably, combining these data with neutralization potency and mutational analysis can guide selection of broadly protective therapeutic cocktails.

Overall, antibodies from community RBD-1 through RBD-4 and those directed against the NTD are generally more potent than antibodies of other communities. The high potency and nonoverlapping epitopes of RBD- and NTD-directed antibodies make them attractive as pairs for therapeutic cocktails. However, members of each of these groups are also highly susceptible to neutralization escape by mutations and deletions found in emerging VOCs. Indeed, a CoVIC bispecific antibody targeting the RBD-1 and NTD-1 sites could still neutralize single point mutations in the RBD (where the NTD arm could compensate) but was ineffective against B.1.351 (Beta) and P.1 (Gamma), which contain mutations that simultaneously escape both arms of the bispecific (fig. S14).

By contrast, RBD-5, RBD-6, and RBD-7 antibodies often have lower potency but are more resistant to escape. Notably, the epitopes targeted by RBD-5, RBD-6, and RBD-7 antibodies have high sequence conservation among the *Sarbecovirus* subgenus of *Betacoronavirus* (fig. S15). Enhanced potency for these communities might be achieved through engineering them as multivalent formats, making them key members of a variant-resistant cocktail that may also be suitable for treating other *Sarbecovirus* infections.

Taken together, the analysis presented here, made possible by broad participation of a few hundred therapeutic candidates in a global study, offers a detailed structural and competitive landscape of key antibody binding sites on spike. The results of this effort can be used to predict and interpret effects of VOCs and for strategic selection of durable therapeutics and cocktails against emerging variants.

## Supplementary Material

20210923-1

## Supplementary Materials

### This PDF file includes:

Materials and Methods Figs. S1 to S15 References (*40*–*56*)

### Other Supplementary Material for this manuscript includes the following:

Tables S1 to S4

MDAR Reproducibility Checklist

## Notes

View/request a protocol for this paper from *Bio-protocol*.
