# Supplementary material for: Defining variant-resistant epitopes targeted by SARS-CoV-2 antibodies: A global consortium study
Source: Science. 2021 Sep 23;374(6566):472–8. doi: 10.1126/science.abh2315 (PMC9302186; doi:10.1126/science.abh2315)
Supplement: 20210923-1 [file science.abh2315.v1.pdf]

Cite as: K. M. Hastie *et al.*, *Science* 10.1126/science.abh2315 (2021).

Defining variant-resistant epitopes targeted by SARS-CoV-2 antibodies: A global consortium study

Kathryn M. Hastie[1]†, Haoyang Li[1]†, Daniel Bedinger[2], Sharon L. Schendel[1], S. Moses Dennison[3], Kan Li[3], Vamseedhar Rayaprolu[1], Xiaoying Yu[1], Colin Mann[1], Michelle Zandonatti[1], Ruben Diaz Avalos[1], Dawid Zyla[1], Tierra Buck[1], Sean Hui[1], Kelly Shaffer[1], Chitra Hariharan[1], Jieyun Yin[1], Eduardo Olmedillas[1], Adrian Enriquez[1], Diptiben Parekh[1], Milite Abraha[3], Elizabeth Feeney[3], Gillian Q. Horn[3], CoVIC-DB team[1], Yoann Aldon[4], Hanif Ali[5], Sanja Aracic[6], Ronald R. Cobb[7], Ross S. Federman[8], Joseph M. Fernandez[9], Jacob Glanville[10], Robin Green[8], Gevorg Grigoryan[8], Ana G. Lujan Hernandez[11], David D. Ho[12], Kuan-Ying A. Huang[13], John Ingraham[8], Weidong Jiang[14], Paul Kellam[15,16], Cheolmin Kim[17], Minsoo Kim[17], Hyeong Mi Kim[17], Chao Kong[18], Shelly J. Krebs[19], Fei Lan[9,20], Guojun Lang[18], Sooyoung Lee[17], Cheuk Lun Leung[8], Junli Liu[14], Yanan Lu[9,21], Anna MacCamy[22], Andrew T. McGuire[22], Anne L. Palser[15], Terence H. Rabbitts[5,23], Zahra Rikhtegaran Tehrani[24], Mohammad M. Sajadi[24], Rogier W. Sanders[4], Aaron K. Sato[11], Liang Schweizer[25], Jimin Seo[17], Bingqing Shen[25], Jonne J. Snitselaar[4], Leonidas Stamatatos[22], Yongcong Tan[18], Milan T. Tomic[26], Marit J. van Gils[4], Sawsan Youssef[10], Jian Yu[12], Tom Z. Yuan[11], Qian Zhang[25], Bjoern Peters[1,27], Georgia D. Tomaras[3], Timothy Germann[2], Erica Ollmann Saphire[1,27]*

[1]Center for Infectious Disease and Vaccine Research, La Jolla Institute for Immunology, 9420 Athena Circle, La Jolla, CA 92037, USA. [2]Carterra, 825 N. 300 W. Ste 309, Salt Lake City, UT 84103, USA. [3]Center for Human Systems Immunology, Departments of Surgery, Immunology, and Molecular Genetics and Microbiology and Duke Human Vaccine Institute, Duke University, Durham, NC, 27701, USA. [4]Department of Medical Microbiology and Infection Prevention, Amsterdam University Medical Centers, Location AMC, University of Amsterdam, Amsterdam Infection & Immunity Institute, 1105 AZ Amsterdam, the Netherlands. [5]Quadrucept Bio Ltd., Cambridge CB23 6DW, UK. [6]Myrio Therapeutics Pty, Ltd., 1 Dalmore Drive, Scoresby, VIC 3179, Australia. [7]National Resilience, Inc., 13200 NW Nano Ct., Alachua, FL 32615, USA. [8]Generate Biomedicines, Inc., 26 Landsdowne Street, Cambridge, MA 02139, USA. [9]Activemotif, Inc., 1914 Palomar Oaks Way, Suite 150, Carlsbad, CA 92008, USA. [10]Centivax, Inc., 201 Gateway Blvd. Floor 1, South San Francisco, CA 94080, USA. [11]Twist Bioscience, 681 Gateway Blvd., South San Francisco, CA 94080, USA. [12]Aaron Diamond AIDS Research Center, Columbia University Vagelos College of Physicians and Surgeons, 701 West 168th St. HHSC 1102, New York, NY 10032, USA. [13]Division of Pediatric Infectious Diseases, Department of Pediatrics, Chang Gung Memorial Hospital and Research Center for Emerging Viral Infections, Chang Gung University, Taoyuan, Taiwan. [14]Shanghai Henlius Biotech, Inc., 9/F, Innov Tower, Zone A, No. 1801 Hongmei Road, Xuhui District, Shanghai, China. [15]Kymab, Ltd., The Bennet Building, Babraham Research Campus, Cambridge CB22 3AT, UK. [16]Department of Infectious Disease, Imperial College, London SW7 2AZ, UK. [17]Celltrion, Inc., Department of Research and Development, 23 Academy-ro Yeonsu-gu Incheon, Republic of Korea. [18]Sanyou Biopharmaceuticals Co., Ltd., No. 188 Xinjunhuan Road, Building 6B-C, 3rd Floor, Minhang District, Shanghai 201114, China. [19]Emerging Infectious Diseases Branch, Walter Reed Army Institute of Research, Silver Spring, MD 20910, USA. [20]Shanghai Key Laboratory of Medical Epigenetics, International Laboratory of Medical Epigenetics and Metabolism, Ministry of Science and Technology, Institutes of Biomedical Sciences, Fudan University, Shanghai, China. [21]AbCipher Biotechnology, 188 Xinjun Ring Road, Building 2, 4th Floor, Minhang District, Shanghai, 201114, China. [22]Fred Hutchinson Cancer Research Center, Vaccines and Infectious Diseases Division, Seattle, WA, USA. [23]Institute of Cancer Research, Centre for Cancer Drug Discovery, London SM2 5NG, UK. [24]Division of Clinical Care and Research, Institute of Human Virology, University of Maryland, Baltimore, MD 21201, USA. [25]HiFiBiO, Inc., 237 Putnam Avenue, Cambridge, MA 02139, USA. [26]National Resilience, Inc., 2061 Challenger Dr., Alameda, CA 94501, USA. [27]Department of Medicine, University of California, San Diego, La Jolla, CA 92037, USA.

†These authors contributed equally to this work.

***Corresponding author. Email: erica@lji.org**

Antibody-based therapeutics and vaccines are essential to combat COVID-19 morbidity and mortality following severe acute respiratory syndrome coronavirus-2 (SARS-CoV-2) infection. Multiple mutations in SARS-CoV-2 that could impair antibody defenses propagated in human-to-human transmission and spillover/spillback events between humans and animals. To develop prevention and therapeutic strategies, we formed an international consortium to map the epitope landscape on the SARS-CoV-2 Spike, defining and structurally illustrating seven receptor-binding domain (RBD)-directed antibody communities with distinct footprints and competition profiles. Pseudovirion-based neutralization assays reveal Spike mutations, individually and clustered together in variants, that impact antibody function among the communities. Key classes of RBD-targeted antibodies maintain neutralization activity against these emerging SARS-CoV-2 variants. These results provide a framework for selecting antibody treatment cocktails and understanding how viral variants might affect antibody therapeutic efficacy.

Cell entry of severe acute respiratory syndrome coronavirus 2 (SARS-CoV-2) is mediated by its surface glycoprotein, Spike. The S1 subunit of Spike contains the N-terminal domain (NTD) and the receptor-binding domain (RBD), which mediates recognition of the host cell receptor angiotensin-converting enzyme 2 (ACE2). The S2 subunit drives fusion

between virus and host cell membranes. Spike, particularly the S1 subunit, is the primary target of neutralizing antibodies against SARS-CoV-2 (*1*).

Since SARS-CoV-2 first emerged, recurrent mutations in Spike arose during both human-to-human transmission (*2*–*4*) and spillover/spillback events between humans and animals (*5*–*8*). Distinct Variants of Concern (VOCs) or Variants of Interest (VOIs), including those first identified in the UK (alpha, B.1.1.7), South Africa (beta, B.1.351), Brazil (gamma, P.1), India (delta, B.1.617.2) and California (epsilon, B.1.429) carry several mutations associated with enhancement of human-to-human transmission (*9*). In particular, the receptor-binding motif (RBM) mutations K417, L452, E484 and N501 affect ACE2-Spike interactions (*10*). Variations at positions N439 and S477 are frequently detected in patient samples (*3*, *11*, *12*), whereas others such as V367F, Y453F and F486L are associated with cross-species transmission (*6*, *8*). The NTD is also highly mutable and is especially prone to deletions: ΔHV69-70 and ΔY144 are both seen in B.1.1.7 and ΔHV69-70 is in the mink-associated Cluster V (*6*). ΔLAL242-244 appears in B.1.351, and ΔFR157-158 is found in B.1.617.2 (*9*). The NTD point mutations S13I and W152C alter disulfide bonding and conformation of the B.1.429 NTD (*13*) (fig. S1).

SARS-CoV-2 will continue to evolve. By understanding antibody footprints and the distinct ways by which antibodies target Spike, we may deduce optimal combinations of mAbs to prevent and treat infection by emerging variants and to minimize the risk of viral escape. We can also gauge the susceptibility of mapped antibodies to new mutations and predict whether newly identified mAbs might also be susceptible to viral escape. Thus, we sought to define functionally important groups in an array of therapeutic candidates, and to dissect how key mutations, both individually and combined as in VOCs, affect antibody-mediated neutralization in a pseudovirus neutralization assay.

The Coronavirus Immunotherapeutic Consortium (CoVIC) was formed to analyze candidate antibody therapeutics side-by-side in standardized assays (*14*) and now includes over 350 monoclonal antibodies (mAbs) directed against the SARS-CoV-2 Spike protein from 56 different partners across four continents (*15*). The panel includes antibodies derived from COVID-19 survivors, phage display, naïve libraries, *in silico* methods and other strategies, each elicited, evaluated and selected using distinct criteria. The panel thus represents a broader and deeper array of antibodies from which both fundamental information and therapeutic cocktails can be derived. With the goals of FAIR (findable, accessible, interoperable, reusable) data analysis and management as well as inclusion of otherwise inaccessible clinical candidates, candidate antibody therapeutics were blinded, and tested in multiple in vitro and in vivo assays with comparative data uploaded into a publicly accessible database (covic.lji.org).

We first measured the affinity of 269 CoVIC mAbs for D614-Hexapro Spike ectodomain trimers and monomeric RBD and NTD, and the ability of each of these mAbs to block ACE2-RBD binding (figs. S2 to S5, table S1, and covic.lji.org). The panel, formed by candidates for therapeutic use, includes NTD- or S2-directed antibodies, but is dominated by those targeting the RBD. In contrast to previous studies that classified mAbs using germline or structural information (*10*, *16*), the 186 RBD-reactive mAbs of CoVIC analyzed here were instead distinguished by a competition profile created by high-throughput surface plasmon resonance (HT-SPR). RBD-directed antibodies can be sorted into seven core "communities" (Fig. 1, fig. S6A, and table S2) that are broadly defined by the competition profiles of each mAb to one another. Communities can be further divided into finer clusters and bins based on their discrete competition with other clusters and/or their ability to compete with ACE2 (Fig. 1 and tables S1 and S2).

To understand the position of each community relative to the others, we next mapped the footprints by negative-stain EM (NS-EM) for 25 example RBD-reactive mAbs chosen to span the range of communities and key clusters (table S3). To have a relatively agnostic view of antibody interactions with Spike, mAbs were not chosen based on germline origin, CDR feature or length, neutralization potency, particular antibody origin (e.g., human, mouse or *in silico*) or format (e.g., IgG, scFv-Fc, VHH-Fc, multivalent).

In parallel, we measured the neutralization activity of 41 RBD-directed mAbs (chosen to span the range of communities and key clusters) as well as a human ACE2-Fc format-based therapeutic candidate (CoVIC-069) fusion format. Neutralization was measured against pseudoviruses displaying the Spike protein bearing (a) the globally dominant G614 variation, (b) 15 single point mutations or deletions represented in circulating strains, (c) constellations of mutations found in four VOCs [B.1.1.1 (alpha), B.1.351 (beta), P.1 (gamma), and B.1.617.2 (delta)] and one VOI [B.1.429 (epsilon)] and (d) two pseudovariants carrying four mutations (termed 4xM, containing G261D, Y453F, F486L, and N501T) or five mutations (termed 5xM, carrying the 4xM mutations plus V367F) identified in human-mink spillover events (fig. S1).

The mAbs in RBD-1 through -3 target the receptor-binding motif (RBM), compete with ACE2, and generally require the RBD to be in the "up" conformation for binding (footprints defined in Fig. 2B, table S3, and covic.lji.org). Community RBD-1 contains hACE2-derived molecules and IgGs (e.g., CoVIC-259, EMD-24335) that largely overlap with the RBM (Fig. 2B, fig. S6B, and table S3). The footprint for RBD-2 mAbs is shifted from the center of the ACE2 binding site toward the peak of the RBM (Fig. 2B, fig. S6B, and table S3). RBD-2 is the largest community and can be divided further into clusters and then bins based on competition with other communities (Fig. 1).

Cluster 2a antibodies (e.g., CoVIC-252, EMD-24339) bind toward the inner face of the RBD and its binding area overlaps highly with that of the therapeutic antibody REGN-10933 (*17*). Antibodies in 2b.1 (e.g., CoVIC-010, EMD-24343; similar to antibody COVA2-39 (*18*)) and 2b.2 (e.g., CoVIC-140, EMD-24383; similar to antibody C144 (*16*)) bind toward the outer face of the RBD and mAbs in bin 2b.3 (e.g., CoVIC-002, EMD-24345; similar to antibody S2E12 (*19*)) bind to the peak of the RBD (Fig. 2B, fig. S7, and table S3). Lastly, RBD-3 mAbs bind down from the center of the ACE2 binding site toward the RBD “mesa” (Fig. 2B and table S2; e.g., CoVIC-080, EMD-24346; similar to antibody ADI-56046 (*20*)).

To simulate the authentic interactions between antibodies and Spike, intact IgGs were used for NS-EM structural analysis whenever possible. RBD-1 IgGs tend to fully occupy all three RBDs on one Spike and often crosslink two Spike trimers, whereas most RBD-2 IgGs tend to bind bivalently to a single Spike trimer (figs. S8, A and B, and S9 and table S3). RBD-3 IgGs can crosslink Spikes, and bivalent binding was also observed in some cases (table S3 and fig. S9).

General epitope position, and particularly RBM epitopes, is strongly associated with the propensity of particular Spike mutations to escape antibody-mediated neutralization (Fig. 3, fig. S10, and table S4). Neutralization by RBD-2a antibodies is heavily impacted by the K417N mutation, but rarely by the E484K mutation; those in RBD-2b are impacted by the E484K mutation but less so by K417N. Similarly, RBD-2a antibodies are resistant to the L452R mutation found in B.1.429 (epsilon) and B.1.617.2 (delta), while only some RBD-2b antibodies are sensitive to this mutation. Meanwhile, mAbs in RBD-3 are impacted by both N501T/Y and E484K mutations (Fig. 3, figs. S10 and S11, and table S4). In contrast to RBD-2 and -3, the susceptibility of neutralization activity of antibodies in RBD-1 to particular mutations is more variable (Figs. 2B and 3 and tables S3 and S4).

Regardless of the effect of particular single point mutations, nearly every RBD-1 or -2 mAb analyzed showed additive decreases in potency against pseudovirus carrying constellations of multiple mutations in the RBM (Fig. 3, fig. S10, and table S4). For B.1.351 and P.1, almost all RBD-1 and RBD-2 antibodies analyzed suffer a complete loss of neutralization activity. For example, CoVIC-249 and CoVIC-010 show moderate or no change in IC50 against the single point mutations K417N, E484K and N501Y, but CoVIC-249 loses all neutralization activity and CoVIC-010 potency falls by 1000-fold against B.1.351 (beta) and P.1 (gamma) which contain all three mutations. Many RBD-2 antibodies also lose activity against the 4xM mink pseudovariant that carries Y453F, F486L and N501T mutations (Fig. 3, figs. S1B and S10, and table S4).

In contrast, most RBD-1 and RBD-2 antibodies retain neutralization activity against B.1.1.7 (alpha), B.1.429 (epsilon) and B.1.617.2 (delta) variants, which each contain only one or two RBM-located mutations (N501Y, L452R or T478K/L452R respectively). Curiously, the V367F mutation identified in mink populations enhances neutralization by some RBD-2 mAbs and in some cases this mutation can offset decreases in potency resulting from other single point mutations. For example, CoVIC-040 has a 14- and 8-fold decrease in potency against the F486L mutation and the F486L-containing 4xM mink pseudovariant, respectively, but only a 4-fold decrease against the 5xM mink pseudovariant, which contains V367F in addition to the four mutations present in 4xM (Fig. 3, fig. S10, and table S4). V367 is adjacent to an N-linked glycan at position 343, which was recently implicated in providing a gating mechanism for the RBD (*21*). Substitution of valine with phenylalanine could alter the local environment of the N343 glycan moieties and enable the RBD to adopt a conformation more amenable to antibody interaction.

Antibodies in communities RBD-4 and RBD-5 bind to the outer face of the RBD and, like the Class 2 and Class 3 mAbs previously defined in (*16*), can do so in either the “up” or “down” RBD conformation without steric hindrance (Fig. 2C, figs. S6 and S12, and table S3). The footprints of these groups largely overlap, but RBD-4 mAbs bind toward the outer edge of the RBM and can block ACE2 (e.g., CoVIC-094, EMD-24350; similar to antibody C002 (*16*)), whereas RBD-5 mAbs bind away from the RBM, toward the “S309” site and do not block ACE2 (e.g., CoVIC-134, EMD-24384; similar to antibody REGN-10987 (*17*)) (*10*) (Figs. 1B and 2C, figs. S5 and S6B, and tables S1 to S3). Some RBD-4 and RBD-5 IgGs can crosslink Spike trimers in solution (fig. S8C and table S3).

Interestingly, according to the five RBD-5 IgGs we imaged, only those IgGs that show Spike-cross linking tendency have potent neutralizing activity (Fig. 1B, fig. S13, and table S3). A recent cryo-electron tomography study showed native Spike trimers on the SARS-CoV-2 virion surface tilt at variable degrees relative to the viral envelope (*22*). This finding provides a possibility for IgG-mediated Spike crosslinking on virions, and may contribute to the mechanism of neutralization of the RBD-5 mAbs in the absence of ACE2 blocking (fig. S8D).

Most RBD-4 mAbs are impacted by E484K and/or L452R (represented in the B.1.429 variant) mutations (Fig. 3, fig. S10, and table S4), and some are impacted by the N439K mutation, which is highly represented in sequences worldwide (*3*). RBD-5 mAbs, however, show broad resistance to nearly all mutations analyzed, with only two mAbs in this group showing moderate decreases in potency against V367F and N439K (Fig. 3, fig. S10, and table S4).

RBD-6 (e.g., CoVIC-250, EMD-24352) and RBD-7 (e.g., CoVIC-063, EMD-24353) antibodies bind to the inner face of the RBD and access a previously described cryptic epitope (*23*, *24*) (Fig. 2D, fig. S6B, and table S3). Like Class 4 antibodies described in (*16*), binding of Spike by RBD-6 and RBD-7

antibodies requires two RBDs to be in the "up" configuration (fig. S12). The representative IgGs in RBD-6 and RBD-7 each show stronger propensities to crosslink Spike trimers than RBM-directed antibodies (fig. S9 and table S3). RBD-6 and RBD-7 antibodies primarily vary in their competition with RBD-2a antibodies: the downward shift of the RBD-7 footprint on the inner face of the RBD relative to the RBD-6 footprint would allow simultaneous binding of RBD-2a antibodies with RBD-7, but not RBD-6, antibodies (Figs. 1B and 2D, fig. S6B, and table S2). This cryptic RBD-6/7 site is also recognized by antibodies COVA1-16 (*23*) and CR3022 (*24*). Here, strategies of site recognition are further subdivided by competition subgroups, information useful for interpreting differences and antibody behavior and strategies for cocktail selection.

All RBD-6 and RBD-7a antibodies block ACE2, but antibodies in RBD-7b and 7c do not (Fig. 1B and tables S1 to S3). The representatives from the RBD-7b and 7c clusters (CR3022 and CoVIC-021, respectively) demonstrate poor neutralization of pseudoviruses in our assay. The distinct difference in neutralization behavior between 7a and 7b/7c suggests that at this cryptic epitope, competition with ACE2 is a determinant of neutralization (Fig. 1B and table S4) (*25*). Importantly, due to their location away from the RBM, RBD-6 and RBD-7 antibodies are resistant to the mutations and variants analyzed (Fig. 3, fig. S10, and table S4).

Previous reports identified a "supersite" as the primary target for neutralizing antibodies directed against the NTD (*26*). In addition to RBD-directed antibodies, we also analyzed four CoVIC NTD-directed antibodies by NS-EM and in neutralization assays. Together these four antibodies, grouped as NTD-1 through NTD-3, encompass the approximate boundaries of the supersite. The two NTD-1 antibodies bind from the top side of NTD to cover the NTD N terminus and residue Y144 (Fig. 4, e.g., CoVIC-247, EMD-24355 and Table S3). The NTD-1 epitope overlaps with that of mAb 4A8 (*27*) and other "supersite" binders (*28*, *29*). The NTD-2 antibody (CoVIC-245, EMD-24360) approaches from the front side of NTD and contacts Y144 as well as residues H69, V70, W152 and G261, all of which are deleted or substituted in emerging variants (Fig. 4 and fig. S1). The NTD-2 footprint is similar to the footprint of antibodies in the "antigenic site V" group described in (*26*). The NTD-3 mAb (CoVIC-020, EMD-24356) binds to the left side of the NTD, proximal to the RBD of the adjacent monomer and in contact with residue W152 (Fig. 4B). The NTD-3 mAb represents a novel epitope and binding location of an anti-NTD antibody.

Unlike the RBD-directed antibodies, for which neutralization escape is strongly associated with antibody footprint, the NTD-directed antibodies are conformationally sensitive and affected by mutations outside of the discrete footprint. This finding is consistent with that for antibodies elicited by vaccines (*30*). Each of the four NTD mAbs analyzed exhibit a decreased or total loss of neutralization capacity for one or more of the NTD-located deletions (Δ69/70, ΔY144, Δ157-158 and Δ242-244) found in circulating VOCs, regardless of their binding location on NTD (Fig. 4C, fig. S10, and table S4). All NTD mAbs were impacted by P.1 (gamma), which lacks deletions and instead has several point mutations in the NTD. For B.1.429 (epsilon), altered disulfide bonding in the NTD arising from the S13I and W152C mutations (*13*) also abrogated mAb-mediated neutralization. Our results indicate that NTD mutations decrease not only neutralization potency but also the total fraction of virus neutralized (fig. S10).

Several therapeutic antibody cocktails comprising pairs of different mAbs against Spike are currently under investigation for post-exposure treatment of COVID-19 (*16*, *17*, *31*, *32*). However, the potency of some antibodies in these cocktails is compromised by emerging SARS-CoV-2 variants (*33*, *34*). Meanwhile, exposure of virus to monoclonal or polyclonal antibodies can promote antibody-resistant mutations in Spike (*34*–*37*). Notably, SARS-CoV-2 variants that share critical mutations with B.1.1.7 (alpha) were isolated from an immunocompromised COVID-19 patient who received three rounds of convalescent plasma treatment, indicating that even a polyclonal therapeutic can drive evolution of resistant virus strains in unresolved infections (*38*).

Potency, variant-resistance and the ability to co-bind are important considerations when selecting antibodies for therapeutic cocktails. The analysis of the 186 RBD-directed mAbs presented here, each donated by different groups around the globe and each selected in different ways, describes discrete antibody communities, and functionally relevant sub-clusters and/or bins. This analysis provides a competition grid, and a framework for cocktail selection. Notably, combining this data with neutralization potency and mutational analysis can guide selection of broadly protective therapeutic cocktails.

Overall, antibodies from community RBD-1 through RBD-4 and those directed against the NTD are generally more potent than antibodies of other communities. The high potency and non-overlapping epitopes of RBD- and NTD-directed antibodies make them attractive as pairs for therapeutic cocktails. However, members of each of these groups are also highly susceptible to neutralization escape by mutations and deletions found in emerging VOCs. Indeed, a CoVIC bispecific antibody targeting the RBD-1 and NTD-1 sites could still neutralize single point mutations in the RBD (where the NTD arm could compensate), but was ineffective against B.1.351 (beta) and P.1 (gamma), which contain mutations that simultaneously escape both arms of the bispecific (fig. S14).

In contrast, RBD-5, -6 and -7 antibodies often have lower potency but are more resistant to escape. Notably, the epitopes targeted by RBD-5, -6, and -7 antibodies have high sequence conservation among the *Sarbecovirus* subgenus of *Betacoronavirus* (fig. S15). Enhanced potency for these

communities might be achieved through engineering them as multivalent formats, making them key members of a variant-resistant cocktail that could also be suitable for treating other *Sarbecovirus* infections.

Taken together, the analysis presented here, made possible by broad participation of a few hundred therapeutic candidates in a global study, offers a detailed structural and competitive landscape of key antibody binding sites on Spike. The results of this effort can be used to predict and interpret effects of VOCs, and for strategic selection of durable therapeutics and cocktails against emerging variants.

REFERENCES AND NOTES

1. L. Piccoli, Y.-J. Park, M. A. Tortorici, N. Czudnochowski, A. C. Walls, M. Beltramello, C. Silacci-Fregni, D. Pinto, L. E. Rosen, J. E. Bowen, O. J. Acton, S. Jaconi, B. Guarino, A. Minola, F. Zatta, N. Sprugasci, J. Bassi, A. Peter, A. De Marco, J. C. Nix, F. Mele, S. Jovic, B. F. Rodriguez, S. V. Gupta, F. Jin, G. Piumatti, G. Lo Presti, A. F. Pellanda, M. Biggiogero, M. Tarkowski, M. S. Pizzuto, E. Cameroni, C. Havenar-Daughton, M. Smithey, D. Hong, V. Lepori, E. Albanese, A. Ceschi, E. Bernasconi, L. Elzi, P. Ferrari, C. Garzoni, A. Riva, G. Snell, F. Sallusto, K. Fink, H. W. Virgin, A. Lanzavecchia, D. Corti, D. Veesler, Mapping Neutralizing and Immunodominant Sites on the SARS-CoV-2 Spike Receptor-Binding Domain by Structure-Guided High-Resolution Serology. *Cell* **183**, 1024–1042.e21 (2020). doi:10.1016/j.cell.2020.09.037 Medline
2. B. Korber, W. M. Fischer, S. Gnanakaran, H. Yoon, J. Theiler, W. Abfalterer, N. Hengartner, E. E. Giorgi, T. Bhattacharya, B. Foley, K. M. Hastie, M. D. Parker, D. G. Partridge, C. M. Evans, T. M. Freeman, T. I. de Silva, C. McDanal, L. G. Perez, H. Tang, A. Moon-Walker, S. P. Whelan, C. C. LaBranche, E. O. Saphire, D. C. Montefiori; Sheffield COVID-19 Genomics Group, Tracking Changes in SARS-CoV-2 Spike: Evidence that D614G Increases Infectivity of the COVID-19 Virus. *Cell* **182**, 812–827.e19 (2020). doi:10.1016/j.cell.2020.06.043 Medline
3. E. C. Thomson, L. E. Rosen, J. G. Shepherd, R. Spreafico, A. da Silva Filipe, J. A. Wojcechowskyj, C. Davis, L. Piccoli, D. J. Pascall, J. Dillen, S. Lytras, N. Czudnochowski, R. Shah, M. Meury, N. Jesudason, A. De Marco, K. Li, J. Bassi, A. O'Toole, D. Pinto, R. M. Colquhoun, K. Culap, B. Jackson, F. Zatta, A. Rambaut, S. Jaconi, V. B. Sreenu, J. Nix, I. Zhang, R. F. Jarrett, W. G. Glass, M. Beltramello, K. Nomikou, M. Pizzuto, L. Tong, E. Cameroni, T. I. Croll, N. Johnson, J. Di Iulio, A. Wickenhagen, A. Ceschi, A. M. Harbison, D. Mair, P. Ferrari, K. Smollett, F. Sallusto, S. Carmichael, C. Garzoni, J. Nichols, M. Galli, J. Hughes, A. Riva, A. Ho, M. Schiuma, M. G. Semple, P. J. M. Openshaw, E. Fadda, J. K. Baillie, J. D. Chodera, S. J. Rihn, S. J. Lycett, H. W. Virgin, A. Telenti, D. Corti, D. L. Robertson, G. Snell; ISARIC4C Investigators; COVID-19 Genomics UK (COG-UK) Consortium, Circulating SARS-CoV-2 spike N439K variants maintain fitness while evading antibody-mediated immunity. *Cell* **184**, 1171–1187.e20 (2021). doi:10.1016/j.cell.2021.01.037 Medline
4. CDC, Emerging SARS-CoV-2 Variants (2021); www.cdc.gov/coronavirus/2019-ncov/more/science-and-research/scientific-brief-emerging-variants.html.
5. N. Oreshkova, R. J. Molenaar, S. Vreman, F. Harders, B. B. Oude Munnink, R. W. Hakze-van der Honing, N. Gerhards, P. Tolsma, R. Bouwstra, R. S. Sikkema, M. G. Tacken, M. M. de Rooij, E. Weesendorp, M. Y. Engelsma, C. J. Bruschke, L. A. Smit, M. Koopmans, W. H. van der Poel, A. Stegeman, SARS-CoV-2 infection in farmed minks, the Netherlands, April and May 2020. *Euro Surveill.* **25**, (2020). doi:10.2807/1560-7917.ES.2020.25.23.2001005 Medline
6. WHO, | SARS-CoV-2 mink-associated variant strain – Denmark (2020); www.who.int/csr/don/06-november-2020-mink-associated-sars-cov2-denmark/en/.
7. B. B. Oude Munnink, R. S. Sikkema, D. F. Nieuwenhuijse, R. J. Molenaar, E. Munger, R. Molenkamp, A. van der Spek, P. Tolsma, A. Rietveld, M. Brouwer, N. Bouwmeester-Vincken, F. Harders, R. Hakze-van der Honing, M. C. A. Wegdam-Blans, R. J. Bouwstra, C. GeurtsvanKessel, A. A. van der Eijk, F. C. Velkers, L. A. M. Smit, A. Stegeman, W. H. M. van der Poel, M. P. G. Koopmans, Transmission of SARS-CoV-2 on mink farms between humans and mink and back to humans. *Science* **371**, 172–177 (2021). doi:10.1126/science.abe5901 Medline
8. L. van Dorp, C. C. S. Tan, S. D. Lam, D. Richard, C. Owen, D. Berchtold, C. Orengo, F. Balloux, Recurrent mutations in SARS-CoV-2 genomes isolated from mink point to rapid host-adaptation. Cold Spring Harbor Laboratory (2020), p. 2020.11.16.384743.
9. CDC, SARS-CoV-2 Variants (2021); www.cdc.gov/coronavirus/2019-ncov/cases-updates/variant-surveillance/variant-info.html.
10. M. Yuan, D. Huang, C.-C. D. Lee, N. C. Wu, A. M. Jackson, X. Zhu, H. Liu, L. Peng, M. J. van Gils, R. W. Sanders, D. R. Burton, S. M. Reincke, H. Prüss, J. Kreye, D. Nemazee, A. B. Ward, I. A. Wilson, Structural and functional ramifications of antigenic drift in recent SARS-CoV-2 variants. bioRxiv (2021); doi:10.1101/2021.02.16.430500.
11. J. Chen, R. Wang, M. Wang, G.-W. Wei, Mutations Strengthened SARS-CoV-2 Infectivity. *J. Mol. Biol.* **432**, 5212–5226 (2020). doi:10.1016/j.jmb.2020.07.009 Medline
12. E. B. Hodcroft, M. Zuber, S. Nadeau, K. H. D. Crawford, J. D. Bloom, D. Veesler, T. G. Vaughan, I. Comas, F. G. Candelas, T. Stadler, R. A. Neher, Emergence and spread of a SARS-CoV-2 variant through Europe in the summer of 2020. medRxiv (2020); doi:10.1101/2020.10.25.20219063.
13. M. McCallum, J. Bassi, A. D. Marco, A. Chen, A. C. Walls, J. D. Iulio, M. A. Tortorici, M.-J. Navarro, C. Silacci-Fregni, C. Saliba, M. Agostini, D. Pinto, K. Culap, S. Bianchi, S. Jaconi, E. Cameroni, J. E. Bowen, S. W. Tilles, M. S. Pizzuto, S. B. Guastalla, G. Bona, A. F. Pellanda, C. Garzoni, W. C. Van Voorhis, L. E. Rosen, G. Snell, A. Telenti, H. W. Virgin, L. Piccoli, D. Corti, D. Veesler, SARS-CoV-2 immune evasion by variant B.1.427/B.1.429. bioRxiv (2021); doi:10.1101/2021.03.31.437925.
14. F. S. Collins, J. Woodcock, B. S. Graham, A. Arvin, P. Bieniasz, D. Ho, G. Alter, M. Nussenzweig, D. Burton, J. Tavel, Others, Therapeutic Neutralizing Monoclonal Antibodies: Report of a Summit sponsored by Operation Warp Speed and the National Institutes of Health (2020); www.nih.gov/sites/default/files/research-training/initiatives/activ/20200909-mAb-summit-pub.pdf.
15. Coronavirus Immunotherapy Consortium (2020); https://covic.lji.org/.
16. C. O. Barnes, C. A. Jette, M. E. Abernathy, K. A. Dam, S. R. Esswein, H. B. Gristick, A. G. Malyutin, N. G. Sharaf, K. E. Huey-Tubman, Y. E. Lee, D. F. Robbiani, M. C. Nussenzweig, A. P. West Jr., P. J. Bjorkman, SARS-CoV-2 neutralizing antibody structures inform therapeutic strategies. *Nature* **588**, 682–687 (2020). doi:10.1038/s41586-020-2852-1 Medline
17. J. Hansen, A. Baum, K. E. Pascal, V. Russo, S. Giordano, E. Wloga, B. O. Fulton, Y. Yan, K. Koon, K. Patel, K. M. Chung, A. Hermann, E. Ullman, J. Cruz, A. Rafique, T. Huang, J. Fairhurst, C. Libertiny, M. Malbec, W.-Y. Lee, R. Welsh, G. Farr, S. Pennington, D. Deshpande, J. Cheng, A. Watty, P. Bouffard, R. Babb, N. Levenkova, C. Chen, B. Zhang, A. Romero Hernandez, K. Saotome, Y. Zhou, M. Franklin, S. Sivapalasingam, D. C. Lye, S. Weston, J. Logue, R. Haupt, M. Frieman, G. Chen, W. Olson, A. J. Murphy, N. Stahl, G. D. Yancopoulos, C. A. Kyratsous, Studies in humanized mice and convalescent humans yield a SARS-CoV-2 antibody cocktail. *Science* **369**, 1010–1014 (2020). doi:10.1126/science.abd0827 Medline
18. N. C. Wu, M. Yuan, H. Liu, C. D. Lee, X. Zhu, S. Bangaru, J. L. Torres, T. G. Caniels, P. J. M. Brouwer, M. J. van Gils, R. W. Sanders, A. B. Ward, I. A. Wilson, An Alternative Binding Mode of IGHV3-53 Antibodies to the SARS-CoV-2 Receptor Binding Domain. *Cell Rep.* **33**, 108274 (2020). doi:10.1016/j.celrep.2020.108274 Medline
19. M. A. Tortorici, M. Beltramello, F. A. Lempp, D. Pinto, H. V. Dang, L. E. Rosen, M. McCallum, J. Bowen, A. Minola, S. Jaconi, F. Zatta, A. De Marco, B. Guarino, S. Bianchi, E. J. Lauron, H. Tucker, J. Zhou, A. Peter, C. Havenar-Daughton, J. A. Wojcechowskyj, J. B. Case, R. E. Chen, H. Kaiser, M. Montiel-Ruiz, M. Meury, N. Czudnochowski, R. Spreafico, J. Dillen, C. Ng, N. Sprugasci, K. Culap, F. Benigni, R. Abdelnabi, S. C. Foo, M. A. Schmid, E. Cameroni, A. Riva, A. Gabrieli, M. Galli, M. S. Pizzuto, J. Neyts, M. S. Diamond, H. W. Virgin, G. Snell, D. Corti, K. Fink, D. Veesler, Ultrapotent human antibodies protect against SARS-CoV-2 challenge via multiple mechanisms. *Science* **370**, 950–957 (2020). doi:10.1126/science.abe3354 Medline
20. A. Z. Wec, D. Wrapp, A. S. Herbert, D. P. Maurer, D. Haslwanter, M. Sakharkar, R. K. Jangra, M. E. Dieterle, A. Lilov, D. Huang, L. V. Tse, N. V. Johnson, C.-L. Hsieh, N. Wang, J. H. Nett, E. Champney, I. Burnina, M. Brown, S. Lin, M. Sinclair, C. Johnson, S. Pudi, R. Bortz 3rd, A. S. Wirchnianski, E. Laudermilch, C. Florez, J. M.

Fels, C. M. O'Brien, B. S. Graham, D. Nemazee, D. R. Burton, R. S. Baric, J. E. Voss, K. Chandran, J. M. Dye, J. S. McLellan, L. M. Walker, Broad neutralization of SARS-related viruses by human monoclonal antibodies. *Science* **369**, 731–736 (2020). doi:10.1126/science.abc7424 Medline

21. T. Sztain, S.-H. Ahn, A. T. Bogetti, L. Casalino, J. A. Goldsmith, R. S. McCool, F. L. Kearns, J. Andrew McCammon, J. S. McLellan, L. T. Chong, R. E. Amaro, A glycan gate controls opening of the SARS-CoV-2 spike protein. Cold Spring Harbor Laboratory (2021), p. 2021.02.15.431212.
22. H. Yao, Y. Song, Y. Chen, N. Wu, J. Xu, C. Sun, J. Zhang, T. Weng, Z. Zhang, Z. Wu, L. Cheng, D. Shi, X. Lu, J. Lei, M. Crispin, Y. Shi, L. Li, S. Li, Molecular Architecture of the SARS-CoV-2 Virus. *Cell* **183**, 730–738.e13 (2020). doi:10.1016/j.cell.2020.09.018 Medline
23. H. Liu, N. C. Wu, M. Yuan, S. Bangaru, J. L. Torres, T. G. Caniels, J. van Schooten, X. Zhu, C.-C. D. Lee, P. J. M. Brouwer, M. J. van Gils, R. W. Sanders, A. B. Ward, I. A. Wilson, Cross-neutralization of a SARS-CoV-2 antibody to a functionally conserved site is mediated by avidity. bioRxiv (2020); doi:10.1101/2020.08.02.233536.
24. M. Yuan, H. Liu, N. C. Wu, C. D. Lee, X. Zhu, F. Zhao, D. Huang, W. Yu, Y. Hua, H. Tien, T. F. Rogers, E. Landais, D. Sok, J. G. Jardine, D. R. Burton, I. A. Wilson, Structural basis of a shared antibody response to SARS-CoV-2. *Science* **369**, 1119–1123 (2020). doi:10.1126/science.abd2321 Medline
25. J. Huo, Y. Zhao, J. Ren, D. Zhou, H. M. E. Duyvesteyn, H. M. Ginn, L. Carrique, T. Malinauskas, R. R. Ruza, P. N. M. Shah, T. K. Tan, P. Rijal, N. Coombes, K. R. Bewley, J. A. Tree, J. Radecke, N. G. Paterson, P. Supasa, J. Mongkolsapaya, G. R. Screaton, M. Carroll, A. Townsend, E. E. Fry, R. J. Owens, D. I. Stuart, Neutralization of SARS-CoV-2 by Destruction of the Prefusion Spike. *Cell Host Microbe* **28**, 445–454.e6 (2020). doi:10.1016/j.chom.2020.06.010 Medline
26. M. McCallum, A. De Marco, F. A. Lempp, M. A. Tortorici, D. Pinto, A. C. Walls, M. Beltramello, A. Chen, Z. Liu, F. Zatta, S. Zepeda, J. di Iulio, J. E. Bowen, M. Montiel-Ruiz, J. Zhou, L. E. Rosen, S. Bianchi, B. Guarino, C. S. Fregni, R. Abdelnabi, S. C. Foo, P. W. Rothlauf, L.-M. Bloyet, F. Benigni, E. Cameroni, J. Neyts, A. Riva, G. Snell, A. Telenti, S. P. J. Whelan, H. W. Virgin, D. Corti, M. S. Pizzuto, D. Veesler, N-terminal domain antigenic mapping reveals a site of vulnerability for SARS-CoV-2. *Cell* **184**, 2332–2347.e16 (2021). doi:10.1016/j.cell.2021.03.028 Medline
27. X. Chi, R. Yan, J. Zhang, G. Zhang, Y. Zhang, M. Hao, Z. Zhang, P. Fan, Y. Dong, Y. Yang, Z. Chen, Y. Guo, J. Zhang, Y. Li, X. Song, Y. Chen, L. Xia, L. Fu, L. Hou, J. Xu, C. Yu, J. Li, Q. Zhou, W. Chen, A neutralizing human antibody binds to the N-terminal domain of the Spike protein of SARS-CoV-2. *Science* **369**, 650–655 (2020). doi:10.1126/science.abc6952 Medline
28. G. Cerutti, Y. Guo, T. Zhou, J. Gorman, M. Lee, M. Rapp, E. R. Reddem, J. Yu, F. Bahna, J. Bimela, Y. Huang, P. S. Katsamba, L. Liu, M. S. Nair, R. Rawi, A. S. Olia, P. Wang, G.-Y. Chuang, D. D. Ho, Z. Sheng, P. D. Kwong, L. Shapiro, Potent SARS-CoV-2 Neutralizing Antibodies Directed Against Spike N-Terminal Domain Target a Single Supersite. Cold Spring Harbor Laboratory (2021), p. 2021.01.10.426120.
29. M. McCallum, A. D. Marco, F. Lempp, M. A. Tortorici, D. Pinto, A. C. Walls, M. Beltramello, A. Chen, Z. Liu, F. Zatta, S. Zepeda, J. di Iulio, J. E. Bowen, M. Montiel-Ruiz, J. Zhou, L. E. Rosen, S. Bianchi, B. Guarino, C. S. Fregni, R. Abdelnabi, S.-Y. Caroline Foo, P. W. Rothlauf, L.-M. Bloyet, F. Benigni, E. Cameroni, J. Neyts, A. Riva, G. Snell, A. Telenti, S. P. J. Whelan, H. W. Virgin, D. Corti, M. S. Pizzuto, D. Veesler, N-terminal domain antigenic mapping reveals a site of vulnerability for SARS-CoV-2. bioRxiv (2021); doi:10.1101/2021.01.14.426475.
30. Y. Cao, A. Yisimayi, Y. Bai, W. Huang, X. Li, Z. Zhang, T. Yuan, R. An, J. Wang, T. Xiao, S. Du, W. Ma, L. Song, Y. Li, X. Li, W. Song, J. Wu, S. Liu, X. Li, Y. Zhang, B. Su, X. Guo, Y. Wei, C. Gao, N. Zhang, Y. Zhang, Y. Dou, X. Xu, R. Shi, B. Lu, R. Jin, Y. Ma, C. Qin, Y. Wang, Y. Feng, J. Xiao, X. S. Xie, Humoral immune response to circulating SARS-CoV-2 variants elicited by inactivated and RBD-subunit vaccines. *Cell Res.* **31**, 732–741 (2021). doi:10.1038/s41422-021-00514-9 Medline
31. A. Baum, D. Ajithdoss, R. Copin, A. Zhou, K. Lanza, N. Negron, M. Ni, Y. Wei, K. Mohammadi, B. Musser, G. S. Atwal, A. Oyejide, Y. Goez-Gazi, J. Dutton, E. Clemmons, H. M. Staples, C. Bartley, B. Klaffke, K. Alfson, M. Gazi, O. Gonzalez, E. Dick Jr., R. Carrion Jr., L. Pessaint, M. Porto, A. Cook, R. Brown, V. Ali, J. Greenhouse, T. Taylor, H. Andersen, M. G. Lewis, N. Stahl, A. J. Murphy, G. D. Yancopoulos, C. A. Kyratsous, REGN-COV2 antibodies prevent and treat SARS-CoV-2 infection in rhesus macaques and hamsters. *Science* **370**, 1110–1115 (2020). doi:10.1126/science.abe2402 Medline
32. Clinical trials of monoclonal antibodies to prevent COVID-19 now enrolling (2020); www.nih.gov/news-events/news-releases/clinical-trials-monoclonal-antibodies-prevent-covid-19-now-enrolling.
33. Z. Wang, F. Schmidt, Y. Weisblum, F. Muecksch, C. O. Barnes, S. Finkin, D. Schaefer-Babajew, M. Cipolla, C. Gaebler, J. A. Lieberman, T. Y. Oliveira, Z. Yang, M. E. Abernathy, K. E. Huey-Tubman, A. Hurley, M. Turroja, K. A. West, K. Gordon, K. G. Millard, V. Ramos, J. Da Silva, J. Xu, R. A. Colbert, R. Patel, J. Dizon, C. Unson-O'Brien, I. Shimeliovich, A. Gazumyan, M. Caskey, P. J. Bjorkman, R. Casellas, T. Hatziioannou, P. D. Bieniasz, M. C. Nussenzweig, mRNA vaccine-elicited antibodies to SARS-CoV-2 and circulating variants. *Nature* **592**, 616–622 (2021). doi:10.1038/s41586-021-03324-6 Medline
34. P. Wang, M. S. Nair, L. Liu, S. Iketani, Y. Luo, Y. Guo, M. Wang, J. Yu, B. Zhang, P. D. Kwong, B. S. Graham, J. R. Mascola, J. Y. Chang, M. T. Yin, M. Sobieszczyk, C. A. Kyratsous, L. Shapiro, Z. Sheng, Y. Huang, D. D. Ho, Antibody resistance of SARS-CoV-2 variants B.1.351 and B.1.1.7. *Nature* **593**, 130–135 (2021). doi:10.1038/s41586-021-03398-2 Medline
35. Y. Weisblum, F. Schmidt, F. Zhang, J. DaSilva, D. Poston, J. C. Lorenzi, F. Muecksch, M. Rutkowska, H.-H. Hoffmann, E. Michailidis, C. Gaebler, M. Agudelo, A. Cho, Z. Wang, A. Gazumyan, M. Cipolla, L. Luchsinger, C. D. Hillyer, M. Caskey, D. F. Robbiani, C. M. Rice, M. C. Nussenzweig, T. Hatziioannou, P. D. Bieniasz, Escape from neutralizing antibodies by SARS-CoV-2 spike protein variants. *eLife* **9**, e61312 (2020). doi:10.7554/eLife.61312 Medline
36. Z. Liu, L. A. VanBlargan, P. W. Rothlauf, L.-M. Bloyet, R. E. Chen, S. Stumpf, H. Zhao, J. M. Errico, E. S. Theel, A. H. Ellebedy, D. H. Fremont, M. S. Diamond, S. P. J. Whelan, Landscape analysis of escape variants identifies SARS-CoV-2 spike mutations that attenuate monoclonal and serum antibody neutralization. bioRxiv (2020); doi:10.1101/2020.11.06.372037.
37. A. J. Greaney, T. N. Starr, P. Gilchuk, S. J. Zost, E. Binshtein, A. N. Loes, S. K. Hilton, J. Huddleston, R. Eguia, K. H. D. Crawford, A. S. Dingens, R. S. Nargi, R. E. Sutton, N. Suryadevara, P. W. Rothlauf, Z. Liu, S. P. J. Whelan, R. H. Carnahan, J. E. Crowe Jr., J. D. Bloom, Complete Mapping of Mutations to the SARS-CoV-2 Spike Receptor-Binding Domain that Escape Antibody Recognition. *Cell Host Microbe* **29**, 44–57.e9 (2021). doi:10.1016/j.chom.2020.11.007 Medline
38. S. A. Kemp, D. A. Collier, R. Datir, I. Ferreira, S. Gayed, A. Jahun, M. Hosmillo, C. Rees-Spear, P. Mlcochova, I. U. Lumb, D. J. Roberts, A. Chandra, N. Temperton, K. Sharrocks, E. Blane, J. Briggs, M. J. van Gils, K. Smith, J. R. Bradley, C. Smith, R. Doffinger, L. Ceron-Gutierrez, G. Barcenas-Morales, D. D. Pollock, R. A. Goldstein, A. Smielewska, J. P. Skittrall, T. Gouliouris, I. G. Goodfellow, E. Gkrania-Klotsas, C. Illingworth, L. E. McCoy, R. K. Gupta, Neutralising antibodies in Spike mediated SARS-CoV-2 adaptation. medRxiv (2020); doi:10.1101/2020.12.05.20241927.
39. D. J. Benton, A. G. Wrobel, P. Xu, C. Roustan, S. R. Martin, P. B. Rosenthal, J. J. Skehel, S. J. Gamblin, Receptor binding and priming of the spike protein of SARS-CoV-2 for membrane fusion. *Nature* **588**, 327–330 (2020). doi:10.1038/s41586-020-2772-0 Medline
40. C.-L. Hsieh, J. A. Goldsmith, J. M. Schaub, A. M. DiVenere, H.-C. Kuo, K. Javanmardi, K. C. Le, D. Wrapp, A. G. Lee, Y. Liu, C.-W. Chou, P. O. Byrne, C. K. Hjorth, N. V. Johnson, J. Ludes-Meyers, A. W. Nguyen, J. Park, N. Wang, D. Amengor, J. J. Lavinder, G. C. Ippolito, J. A. Maynard, I. J. Finkelstein, J. S. McLellan, Structure-based design of prefusion-stabilized SARS-CoV-2 spikes. *Science* **369**, 1501–1505 (2020). doi:10.1126/science.abd0826 Medline
41. E. Seydoux, L. J. Homad, A. J. MacCamy, K. R. Parks, N. K. Hurlburt, M. F. Jennewein, N. R. Akins, A. B. Stuart, Y.-H. Wan, J. Feng, R. E. Whaley, S. Singh, M. Boeckh, K. W. Cohen, M. J. McElrath, J. A. Englund, H. Y. Chu, M. Pancera, A. T. McGuire, L. Stamatatos, Analysis of a SARS-CoV-2-Infected Individual Reveals Development of Potent Neutralizing Antibodies with Limited Somatic Mutation. *Immunity* **53**, 98–105.e5 (2020). doi:10.1016/j.immuni.2020.06.001 Medline
42. G. Chao, W. L. Lau, B. J. Hackel, S. L. Sazinsky, S. M. Lippow, K. D. Wittrup, Isolating and engineering human antibodies using yeast surface display. *Nat. Protoc.* **1**, 755–768 (2006). doi:10.1038/nprot.2006.94 Medline
43. A. Miller, S. Carr, T. Rabbitts, H. Ali, Multimeric antibodies with increased valency surpassing functional affinity and potency thresholds using novel formats. *mAbs* **12**, 1752529 (2020). doi:10.1080/19420862.2020.1752529 Medline
44. M. D. Beasley, K. P. Niven, W. R. Winnall, B. R. Kiefel, Bacterial cytoplasmic display platform Retained Display (ReD) identifies stable human germline antibody frameworks. *Biotechnol. J.* **10**, 783–789 (2015). doi:10.1002/biot.201400560 Medline

45. H. Yao, Y. Sun, Y.-Q. Deng, N. Wang, Y. Tan, N.-N. Zhang, X.-F. Li, C. Kong, Y.-P. Xu, Q. Chen, T.-S. Cao, H. Zhao, X. Yan, L. Cao, Z. Lv, D. Zhu, R. Feng, N. Wu, W. Zhang, Y. Hu, K. Chen, R.-R. Zhang, Q. Lv, S. Sun, Y. Zhou, R. Yan, G. Yang, X. Sun, C. Liu, X. Lu, L. Cheng, H. Qiu, X.-Y. Huang, T. Weng, D. Shi, W. Jiang, J. Shao, L. Wang, J. Zhang, T. Jiang, G. Lang, C.-F. Qin, L. Li, X. Wang, Rational development of a human antibody cocktail that deploys multiple functions to confer Pan-SARS-CoVs protection. *Cell Res.* **31**, 25–36 (2021). doi:10.1038/s41422-020-00444-y Medline
46. S. J. Zost, P. Gilchuk, J. B. Case, E. Binshtein, R. E. Chen, J. P. Nkolola, A. Schäfer, J. X. Reidy, A. Trivette, R. S. Nargi, R. E. Sutton, N. Suryadevara, D. R. Martinez, L. E. Williamson, E. C. Chen, T. Jones, S. Day, L. Myers, A. O. Hassan, N. M. Kafai, E. S. Winkler, J. M. Fox, S. Shrihari, B. K. Mueller, J. Meiler, A. Chandrashekar, N. B. Mercado, J. J. Steinhardt, K. Ren, Y.-M. Loo, N. L. Kallewaard, B. T. McCune, S. P. Keeler, M. J. Holtzman, D. H. Barouch, L. E. Gralinski, R. S. Baric, L. B. Thackray, M. S. Diamond, R. H. Carnahan, J. E. Crowe Jr., Potently neutralizing and protective human antibodies against SARS-CoV-2. *Nature* **584**, 443–449 (2020). doi:10.1038/s41586-020-2548-6 Medline
47. S. J. Zost, P. Gilchuk, R. E. Chen, J. B. Case, J. X. Reidy, A. Trivette, R. S. Nargi, R. E. Sutton, N. Suryadevara, E. C. Chen, E. Binshtein, S. Shrihari, M. Ostrowski, H. Y. Chu, J. E. Didier, K. W. MacRenaris, T. Jones, S. Day, L. Myers, F. Eun-Hyung Lee, D. C. Nguyen, I. Sanz, D. R. Martinez, P. W. Rothlauf, L.-M. Bloyet, S. P. J. Whelan, R. S. Baric, L. B. Thackray, M. S. Diamond, R. H. Carnahan, J. E. Crowe Jr., Rapid isolation and profiling of a diverse panel of human monoclonal antibodies targeting the SARS-CoV-2 spike protein. *Nat. Med.* **26**, 1422–1427 (2020). doi:10.1038/s41591-020-0998-x Medline
48. M. T. Tomic, Y. Espinoza, Z. Martinez, K. Pham, R. R. Cobb, D. M. Snow, C. G. Earnhart, T. Pals, E. S. Syar, N. Niemuth, D. J. Kobs, S. Farr-Jones, J. D. Marks, Monoclonal Antibody Combinations Prevent Serotype A and Serotype B Inhalational Botulism in a Guinea Pig Model. *Toxins* **11**, 208 (2019). doi:10.3390/toxins11040208 Medline
49. L. Liu, P. Wang, M. S. Nair, J. Yu, M. Rapp, Q. Wang, Y. Luo, J. F.-W. Chan, V. Sahi, A. Figueroa, X. V. Guo, G. Cerutti, J. Bimela, J. Gorman, T. Zhou, Z. Chen, K.-Y. Yuen, P. D. Kwong, J. G. Sodroski, M. T. Yin, Z. Sheng, Y. Huang, L. Shapiro, D. D. Ho, Potent neutralizing antibodies against multiple epitopes on SARS-CoV-2 spike. *Nature* **584**, 450–456 (2020). doi:10.1038/s41586-020-2571-7 Medline
50. J. Wan, S. Xing, L. Ding, Y. Wang, C. Gu, Y. Wu, B. Rong, C. Li, S. Wang, K. Chen, C. He, D. Zhu, S. Yuan, C. Qiu, C. Zhao, L. Nie, Z. Gao, J. Jiao, X. Zhang, X. Wang, T. Ying, H. Wang, Y. Xie, Y. Lu, J. Xu, F. Lan, Human-IgG-Neutralizing Monoclonal Antibodies Block the SARS-CoV-2 Infection. *Cell Rep.* **32**, 107918 (2020). doi:10.1016/j.celrep.2020.107918 Medline
51. W. Schaefer, J. T. Regula, M. Bähner, J. Schanzer, R. Croasdale, H. Dürr, C. Gassner, G. Georges, H. Kettenberger, S. Imhof-Jung, M. Schwaiger, K. G. Stubenrauch, C. Sustmann, M. Thomas, W. Scheuer, C. Klein, Immunoglobulin domain crossover as a generic approach for the production of bispecific IgG antibodies. *Proc. Natl. Acad. Sci. U.S.A.* **108**, 11187–11192 (2011). doi:10.1073/pnas.1019002108 Medline
52. K. Li, G. Q. Horn, S. M. Alam, G. D. Tomaras, S. M. Dennison, Titrationanalysis: A Tool for High-throughput Analysis of Binding Kinetics Data for Multiple Label-Free Platforms. *Biophys. J.* **120**, 265a–266a (2021). doi:10.1016/j.bpj.2020.11.1701
53. A. Punjani, J. L. Rubinstein, D. J. Fleet, M. A. Brubaker, cryoSPARC: Algorithms for rapid unsupervised cryo-EM structure determination. *Nat. Methods* **14**, 290–296 (2017). doi:10.1038/nmeth.4169 Medline
54. E. F. Pettersen, T. D. Goddard, C. C. Huang, E. C. Meng, G. S. Couch, T. I. Croll, J. H. Morris, T. E. Ferrin, UCSF ChimeraX: Structure visualization for researchers, educators, and developers. *Protein Sci.* **30**, 70–82 (2021). doi:10.1002/pro.3943 Medline
55. C. Zhang, Y. Wang, Y. Zhu, C. Liu, C. Gu, S. Xu, Y. Wang, Y. Zhou, Y. Wang, W. Han, X. Hong, Y. Yang, X. Zhang, T. Wang, C. Xu, Q. Hong, S. Wang, Q. Zhao, W. Qiao, J. Zang, L. Kong, F. Wang, H. Wang, D. Qu, D. Lavillette, H. Tang, Q. Deng, Y. Xie, Y. Cong, Z. Huang, Development and structural basis of a two-MAb cocktail for treating SARS-CoV-2 infections. *Nat. Commun.* **12**, 264 (2021). doi:10.1038/s41467-020-20465-w Medline
56. F. Sievers, A. Wilm, D. Dineen, T. J. Gibson, K. Karplus, W. Li, R. Lopez, H. McWilliam, M. Remmert, J. Söding, J. D. Thompson, D. G. Higgins, Fast, scalable generation of high-quality protein multiple sequence alignments using Clustal Omega. *Mol. Syst. Biol.* **7**, 539 (2011). doi:10.1038/msb.2011.75 Medline

ACKNOWLEDGMENTS

We are grateful for the multiple generous contributions of antibodies to the CoVIC study, with special thanks to Matt Beasley, Shahrad Daraeikia, Vincent Dussupt, Ben Kiefel, Sindy Liao, Chanjuan Liu, Letzibeth Mendez-Rivera, Pramila Rijal, Lisa Schimanski, Pete Smith, Tiong Tan, Alain Townsend, Jack Wang, Run Yan, and Luo Yang. We thank the electron microscope facility of La Jolla Institute for Immunology for the EM data collection. **Funding:** We gratefully acknowledge philanthropic support of the Overton family for this urgent study (Coronavirus Immunotherapeutic Consortium), COVID-19 Therapeutics Accelerator INV-006133 (Coronavirus Immunotherapeutic Consortium), Bill and Melinda Gates Foundation OPP1210938 (Coronavirus Immunotherapeutic Consortium), GHR foundation (Coronavirus Immunotherapeutic Consortium), and NIH/NIAID grant U19 AI142790-S1 (Coronavirus Immunotherapeutic Consortium). We also acknowledge philanthropic support of Carolee Lee, FastGrants from Emergent Ventures at the Mercatus Center, George Mason University for support of essential instrumentation, and T.S. for support of the Saphire laboratory efforts during the pandemic (EOS), Early Postdoc Mobility Fellowship of the Swiss National Science Foundation P2EZP3_195680 (DZ), and Translating Duke Health Immunology Initiative (GT). **Author contributions:** Conceptualization: KH, HL, SS, EOS. Methodology: KH, SS, HL, DB, SD, KL, GT, EOS. Software: JI, GG, KL. Validation: KH, HL, SMD, KL, DB, GT, EOS. Formal analysis: KH, HL, DB. Investigation: KH, HL, SS, DB, DZ, VR, MZ, RDA, CM, TB, XY, SH, KS, CH, JY, EO, AE, DB, SMD, DA, SD, KL, MA, GH, LF. Resources: HA, RF, JJF, JG, RG, GG, JI, ALH, WJ, CK, MK, HMK, CK, FL, GL, SL, AL, JL, YA, ALP, PK, RC, MT, AM, ATM, TR, ZR-T, MS, AS, LS, MJvG, TY, JS, BS, LS, YT, QZ. Data Curation: CoVIC-DB team, BP, SS. Writing – original draft preparation: KH, HL, SS. Writing – review and editing: EOS. Visualization: KH, HL, DB, DZ, KL, MS. Supervision: BP, GT, TG, EOS. Project administration: SS, EOS. Funding acquisition: GT, TG, EOS. The CoVIC-DB team includes Brendan Ha, Mari Kojima, Mahita Jarjapu, Randi Vita, Anaïs Gambiez, Jason A. Greenbaum and James A. Overton under the direction of Bjoern Peters. We thank members of the Antibody Dynamics platform of the Global Health-Vaccine Accelerator Platforms: Sarah Mudrak, Val Bekker, and Karen Makar for program management; David Beaumont and Mark Sampson for data management; and Nathan Eisel, LaTonya Williams for technical expertise. **Competing interests:** Authors declare that they have no competing interests. **Data and materials availability:** EM maps in this study have been uploaded to the EMDataResource. The EMDB access numbers are available in the main text and table S3. Information concerning particular antibodies can be requested through the Coronavirus Immunotherapeutics Consortium at https://covic.lji.org. This work is licensed under a Creative Commons Attribution 4.0 International (CC BY 4.0) license, which permits unrestricted use, distribution, and reproduction in any medium, provided the original work is properly cited. To view a copy of this license, visit https://creativecommons.org/licenses/by/4.0/. This license does not apply to figures/photos/artwork or other content included in the article that is credited to a third party; obtain authorization from the rights holder before using such material.

SUPPLEMENTARY MATERIALS

science.org/doi/10.1126/science.abh2315
Materials and Methods
Figs. S1 to S15
Tables S1 to S4
References (*40–56*)
MDAR Reproducibility Checklist

24 February 2021; accepted 21 September 2021
Published online 23 September 2021
10.1126/science.abh2315

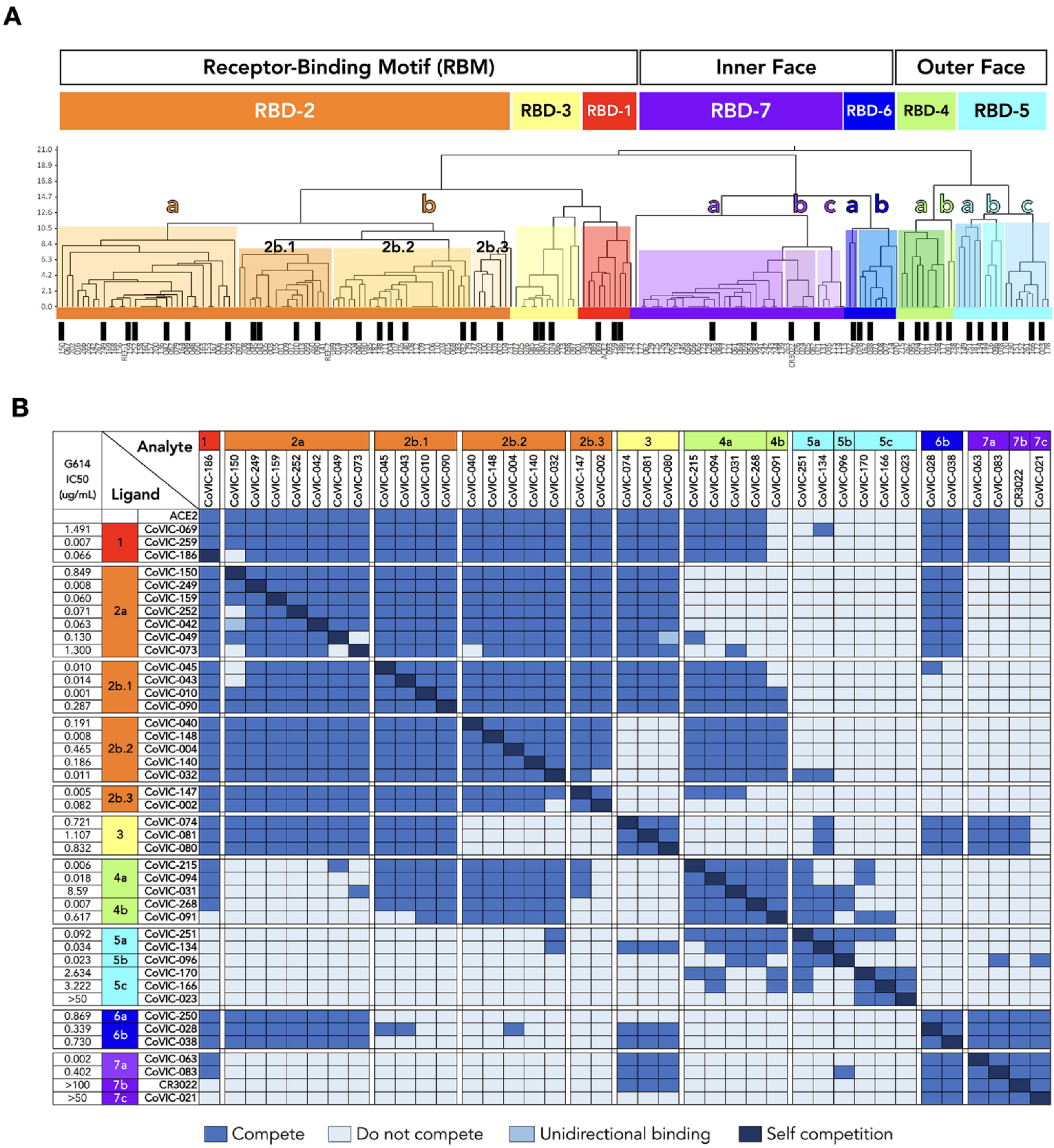
A
Receptor-Binding Motif (RBM)
Inner Face
Outer Face
RBD-2
RBD-3
RBD-1
RBD-7
RBD-6
RBD-4
RBD-5
a
b
2b.1
2b.2
2b.3
c
B
G614
IC50
(ug/mL)
Analyte
Ligand
1
2a
2b.1
2b.2
2b.3
3
4a
4b
5a
5b
5c
6a
6b
7a
7b
7c
ACE2
CoVIC-069
CoVIC-259
CoVIC-186
CoVIC-150
CoVIC-249
CoVIC-159
CoVIC-252
CoVIC-042
CoVIC-049
CoVIC-073
CoVIC-045
CoVIC-043
CoVIC-010
CoVIC-090
CoVIC-040
CoVIC-148
CoVIC-004
CoVIC-140
CoVIC-032
CoVIC-147
CoVIC-002
CoVIC-074
CoVIC-081
CoVIC-080
CoVIC-215
CoVIC-094
CoVIC-031
CoVIC-268
CoVIC-091
CoVIC-251
CoVIC-134
CoVIC-096
CoVIC-170
CoVIC-166
CoVIC-023
CoVIC-250
CoVIC-028
CoVIC-038
CoVIC-063
CoVIC-083
CR3022
CoVIC-021
1.491
0.007
0.066
0.849
0.008
0.060
0.071
0.063
0.130
1.300
0.010
0.014
0.001
0.287
0.191
0.008
0.465
0.186
0.011
0.005
0.082
0.721
1.107
0.832
0.006
0.018
8.59
0.007
0.617
0.092
0.034
0.023
2.634
3.222
>50
0.869
0.339
0.730
0.002
0.402
>100
>50
Compete
Do not compete
Unidirectional binding
Self competition

Fig. 1 (previous page). The antigenic landscape of the SARS-CoV-2 receptor binding domain can be divided into seven binding communities. (**A**) High-throughput SPR was used to determine the competitive relationship between 186 RBD-directed mAbs. The dataset was analyzed by Carterra Epitope software to sort competition profiles of clones into related clusters, which are represented as shared colored regions of the dendrogram. The RBD epitope landscape can be broadly divided into seven communities containing mAbs that bind the receptor-binding motif (RBD-1 through RBD-3), the outer face of the RBD (RBD-4 and RBD-5) or the inner face of the RBD (RBD-6 and RBD-7). Communities can be further divided into smaller clusters (e.g., RBD-2a and -2b) and bins (e.g., RBD-2b.1, -2b.2 and -2b.3) based on their discrete competition with other clusters and/or their ability to compete with ACE2 for Spike binding. Black bars indicate single clones that were used in further analyses. Table S1 lists additional metrics for the indicated mAbs (i.e., ACE2 blocking, kinetic analyses and germline information) and detailed information for the entire CoVIC panel is at covic.lji.org. (**B**) Binary heat-map matrix demonstrating the competition profile for the finer clusters and bins for the subset of single clones indicated by black bars in panel (A). The matrix here contains representative examples. The complete competition matrix for the study is in table S2. RBD-2 can be divided into clusters "a" and "b", which have varying ability to compete with mAbs in RBD-4 (e.g., RBD-2a mAbs do not compete while most RBD-2b mAbs do). Cluster RBD-2b can be divided into three smaller bins that vary in their competition with both RBD-3 and RBD-4 mAbs: those in 2b.1, but not 2b.2 or 2b.3, compete with RBD-3 mAbs whereas mAbs in 2b.1 and 2b.2, but not 2b.3, compete with RBD-4 mAbs. RBD-4 contains mAbs that do (RBD-4a) and do not (RBD-4b) compete with ACE2. RBD-5 and RBD-7 have clusters of mAbs with lower neutralizing potency (i.e., RBD-5c and RBD-7b and RBD-7c) relative to the other cluster in the same community (i.e., RBD-5a and RBD-5b and RBD-7a). Rows and columns indicate the immobilized mAb and injected analyte mAb, respectively. Table S2 shows the complete matrix for competition between all 186 mAbs.

Fig. 2 (next page). Negative stain EM analysis of representatives from each RBD-directed community. (**A**) The location of important emerging mutations in RBD. The Spike trimer (adapted from PDB: 7A94 (*39*)) viewed from the top with one RBD "up" RBD, is shown, with individual Spike monomers colored white, gray and black. The RBM can be topologically divided into three sub-sections: the "Peak" that includes residues F486, S477, T478 and E484, the "Valley" including residues Y453, K417 and L452 and the "Mesa" with residue N501. Stars indicate residues on the central axis of RBD. The "Outer Face" (exposed in the RBD down/closed conformation), and "Inner Face" (buried inside the trimer in the RBD down/closed conformation) define the lateral faces of RBD and "Escarpment" (contains residues V367, N439 and glycan 343). (**B** to **D**) NS-EM footprint of a representative antibody from each community mapped onto an RBD monomer. The colored shading corresponds to the community colors in Fig. 1. The ACE2 binding site is outlined with a dotted line. Side and top views of Spike trimers show the Fab approach angle and binding stoichiometry for each representative. Table S3 shows NS-EM data for all 29 RBD-directed mAbs analyzed.

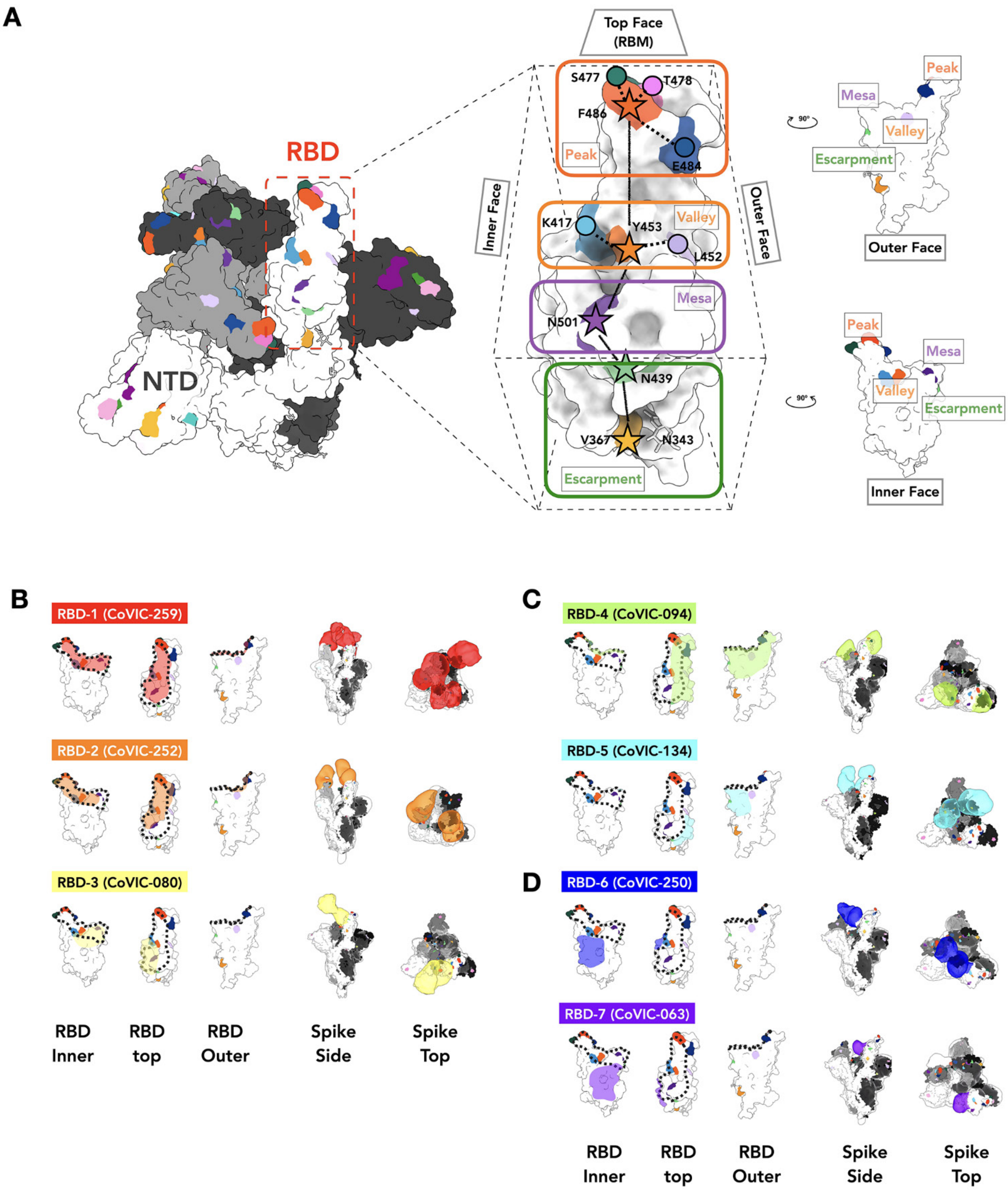
A
RBD
NTD
Top Face (RBM)
S477
T478
F486
E484
Peak
K417
Y453
Valley
L452
Mesa
N501
N439
V367
N343
Escarpment
Inner Face
Outer Face
90°
B
RBD-1 (CoVIC-259)
RBD-2 (CoVIC-252)
RBD-3 (CoVIC-080)
C
RBD-4 (CoVIC-094)
RBD-5 (CoVIC-134)
D
RBD-6 (CoVIC-250)
RBD-7 (CoVIC-063)
RBD Inner
RBD top
RBD Outer
Spike Side
Spike Top

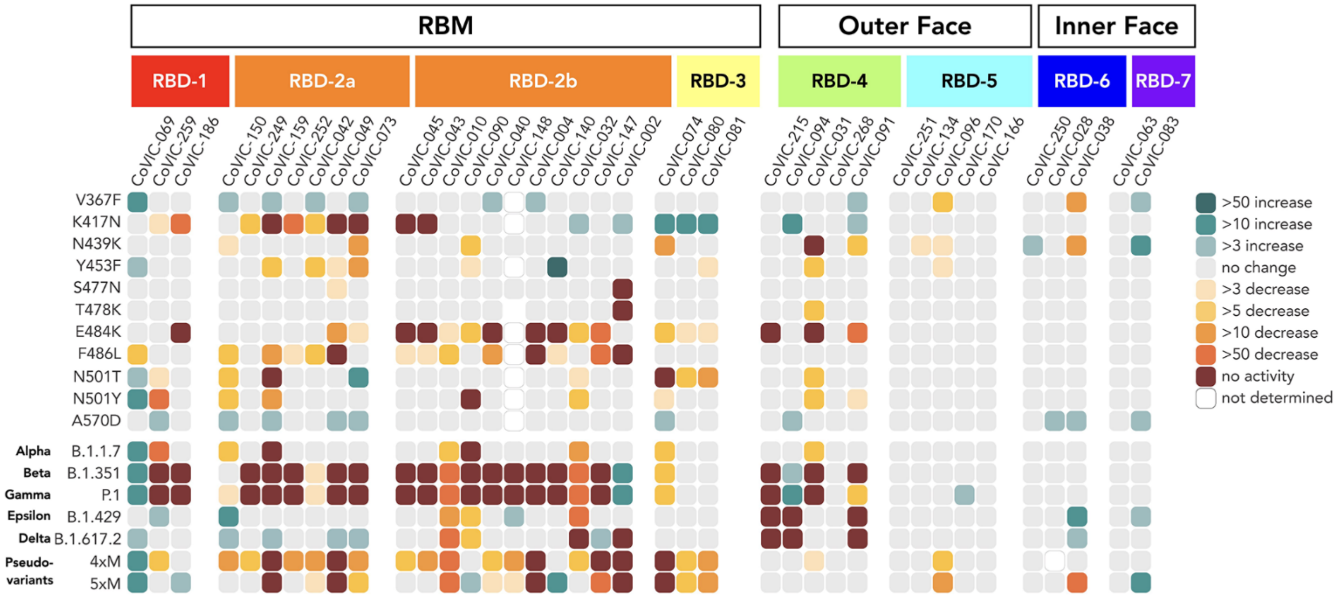

Fig. 3. RBD-5, -6 and -7 antibodies retain neutralization activity against pseudovirus bearing mutations singly or together in VOCs. Fold-change differences in potency for 38 RBD-directed antibodies and an ACE2-Fc fusion (CoVIC-069) are shown in a heat map. In addition to VOCs, we also examined two pseudoviruses bearing clusters of mink-associated mutations: 4xM (G261D, Y453F, F486L and N501T) and 5xM (G261D, Y453F, F486L, N501T and V367F). Fig. S1 lists mutations represented in each variant. Fig. S10 shows neutralization curves for each virus-variant pair and table S4 lists fold-change values corresponding to the heat map.

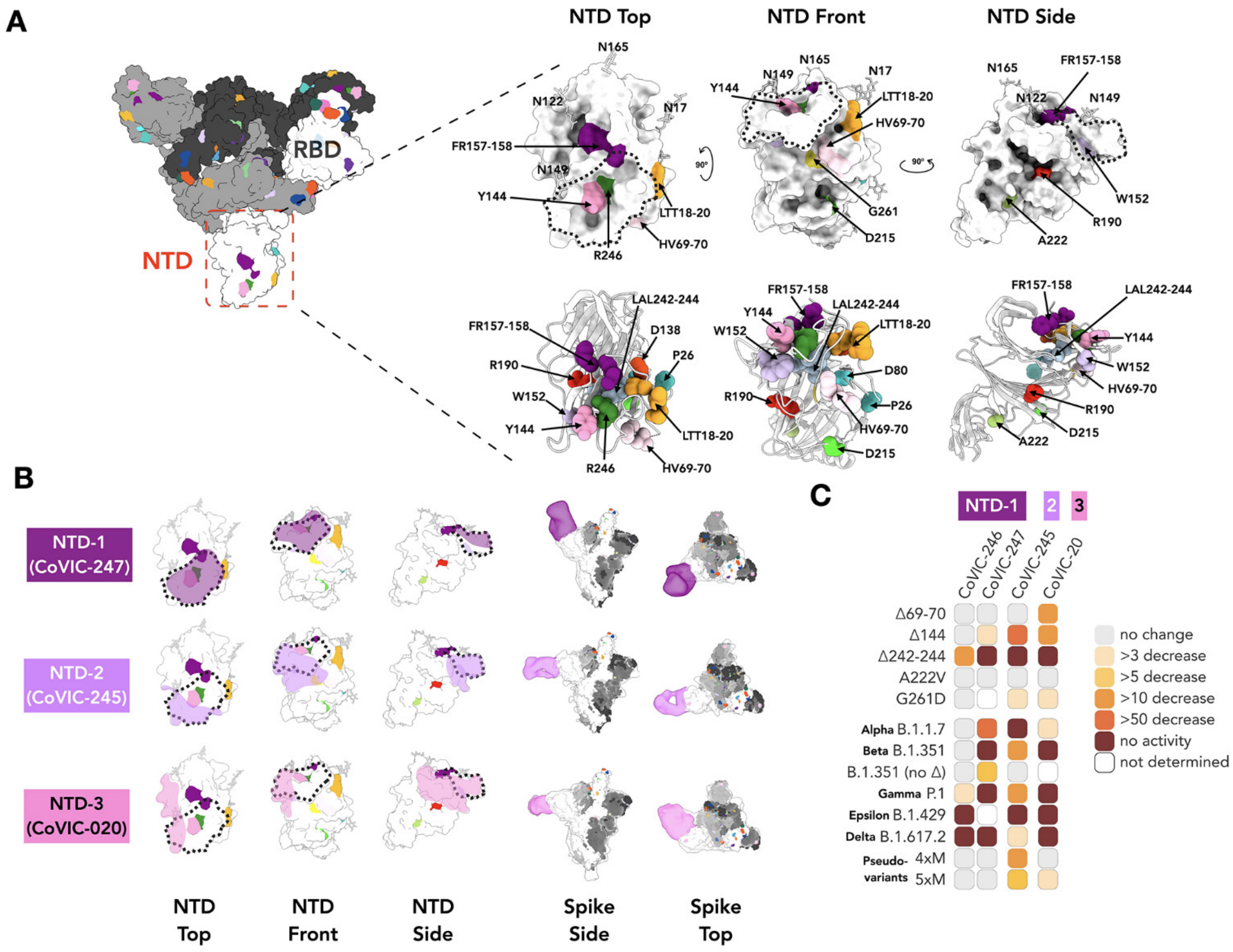

Fig. 4. NS-EM and neutralization analysis of mAb targeting NTD. (**A**) Surface and cartoon (adapted from PDB: 7A94 (*39*)) representation of the Spike NTD. The residue positions of mutations and deletions in circulating VOCs are indicated in three views of NTD. Fig. S1 lists mutations represented in each variant. (**B**) Footprints for three NTD-targeted antibodies with the NTD "supersite" (*26*) indicated as a dotted line. The NTD-directed antibodies shown here define the approximate boundaries of the neutralizing epitope landscape. Additional NS-EM data are in table S3. (**C**) Fold-change in potency of pseudovirus neutralization experiments for each antibody-variant pair.
