# Supplementary material for: Defining variant-resistant epitopes targeted by SARS-CoV-2 antibodies: A global consortium study
Source: Science. 2021 Sep 23;374(6566):472–8. doi: 10.1126/science.abh2315 (PMC9302186; doi:10.1126/science.abh2315)
Supplement: Supplementary file 2 — Materials and Methods Figs. S1 to S15 References (40–56) [file science.abh2315_sm.pdf]

Science AAAS

Supplementary Materials for

Defining variant-resistant epitopes targeted by SARS-CoV-2 antibodies: A global consortium study

Kathryn M. Hastie *et al.*

Corresponding author: Erica Ollmann Saphire, erica@lji.org

Science **374**, 472 (2021)
DOI: 10.1126/science.abh2315

The PDF file includes:

Materials and Methods
Figs. S1 to S15
References

Other Supplementary Material for this manuscript includes the following:

Tables S1 to S4
MDAR Reproducibility Checklist

Materials and Methods

Generation of mutant SARS-CoV-2 Spike proteins

Spike proteins were generated for epitope binning studies and structural biology using the HexaPro background [containing residues 14-1208 (Genbank: MN908947) of the ectodomain, six proline substitutions (F817P, A892P, A899P, A942P, K986P, V987P) (*40*)], as well as the D614G mutation, which was already established in all/most variants associated with spillover/spillback in Northern Europe(*2*), and replacement of cleavage site residues 682-685 ("RRAR" to "GSAS"). The resulting Spike variants were cloned into a phCMV mammalian expression vector containing an N-terminal Gaussia luciferase signal sequence (MGVKVLFALICIAVAEA) and a C-terminal foldon trimerization domain, followed by an HRV-3C cleavage site and a Twin-Strep-Tag. Plasmids were transformed into Stellar competent cells and isolated using a Plasmid Plus Midi kit (Qiagen).

Transient transfection and protein purification

SARS-CoV-2 HexaPro Spike was transiently transfected into Freestyle 293-F or ExpiCHO-S cells (Thermo Fisher). Both cell lines were maintained and transfected according to manufacturer's protocols. Briefly, 293-F cells were grown to a density of 2.0×10^6cells/mL and diluted to 1.0×10^6 cell/mL on the day of transfection (day 0). Plasmid DNA and polyethyleneimine were mixed in Opti-MEM (Gibco), incubated for 25 minutes, and then added to the cells. Cell cultures were incubated at 37 ˚C, 8% CO_2, and 120 RPM, and harvested on day 5. For ExpiCHO cultures, the manufacturer's "High Titer" protocol was used. Briefly, cells were grown to a density of 1×10^7 cells/mL and diluted to 6×10^6 cells/mL on the day of transfection (day 0). Plasmid DNA and Expifectamine were mixed in Opti-PRO SFM (Gibco) according to manufacturer's instructions, and added to the cells. On day 1, cells were fed with manufacturer-supplied feed and enhancer according to the suggested protocol, and cultures were then incubated at 32 ˚C, 5% CO_2 and 115 RPM. ExpiCHO cultures were harvested on day 7. All cultures were clarified by centrifugation, followed by addition of BioLock (IBA Life Sciences), and supernatants were flowed through a 0.22 µM sterile filter and purified on an ÄKTA GO (Cytiva) using a 5mL StrepTrap-HP column equilibrated with TBS buffer (25mM Tris pH 7.6, 200mM NaCl, 0.02% NaN_3), and eluted in TBS buffer supplemented with 5mM d-desthiobiotin (Sigma Aldrich). The strep-tags were cleaved using HRV-3C protease and the proteins were further purified by size-exclusion-chromatography (SEC) on a Superdex 6 increase 10/300 column (GE) in TBS.

CoVIC antibodies

Antibodies analyzed in this study were isolated using various methods. Unless otherwise noted, all antibodies were affinity-purified with Protein A chromatography (GE Life Sciences; MabSelect Sure Protein A affinity resin, Cytiva). Protein concentrations were calculated from the OD_{280} value and calculated extinction coefficient. A total of 27 antibodies used in the study were derived from memory B cells isolated from peripheral blood mononuclear cells (PMBCs) collected from convalescent COVID-19 patients after obtaining informed consent. These antibodies were isolated: (i) using a phage library of single-chain variable fragments in biopanning to screen for phage that bound SARS-CoV-2 RBD. Heavy and light chains for this antibody were inserted into mammalian expression vectors and the antibody was produced from stably transfected CHO-K1 cells; (ii) as described by Seydoux et al.(*41*); (iii) amplification of reverse-transcribed sequences from single B cell cDNA that encode immunoglobulin heavy (IGH) and light (IGL) chains and cloning via homologous recombination into mammalian expression vectors that were used for transient-transfection of Expi293 cells that were allowed to express the antibody for 5-7 days; (iv) labeling of memory B cells with SARS-CoV-2 Spike antigen conjugated with TotalSeq™-C0953 PE Streptavidin (cat# 405265) and loading the single cell suspension onto the 10x Genomics Chromium Controller, microfluidics chip and then preparing the VDJ library based on manufacturer's instructions, selecting the cells capable of binding to the SARS-CoV-2 Spike antigen and cloning of their VDJ sequences into IgG1 heavy and light chain vectors and co-transfection of the resulting plasmid into FreeStyle-293 cells (Thermo Fisher); (v) through affinity-purification of antigen-specific antibodies from patient serum followed by liquid chromatography-mass spectrometry (LC-MS) analysis. In parallel, B cells from the same patients were sequenced to generate an IgG repertoire database with paired VH and VL chains. The mass spectra data together with the paired repertoire database were then used to map the antibody sequence. This antibody was purified from TunaCHO cells (LakePharma); or (vi) using Berkeley Lights Beacon technology to identify and clone SARS-CoV-2 Spike-reactive B-cells. The resulting H/L chain pairs were amplified by rounds of RT and PCR and cloned into phCMV3-based expression vectors encoding the human IgG1 heavy chain constant region and kappa or lambda light chain constant region. For one antibody, a eukaryotic expression vector containing the DNA sequence corresponding to the extracellular domain of human ACE2 fused with human IgG1 Fc was used to produce stably transfected CHO cells that were then used for purification of clinical-grade protein by Shanghai Henlius Biotech, Inc. according to Good Manufacturing Practice guidelines.

Some antibodies were expressed from 293-F cells transfected with the heavy chain and light chain using polyethylenimine. Between 5 and 7 days after transfection the cells were pelleted, the supernatant filtered and the

antibodies were affinity-purified. One set of antibodies was generated via computational generation algorithms (Generate Biomedicines), formatted as scFvs, and tested for binding using yeast display. This set included one antibody that was computationally derived from a camelid VHH antibody. Following successful binding as an scFV, the variable domain was expressed and purified as an Fc-fusion. ScFv constructs fused to a c-myc epitope tag were synthesized as DNA fragments (Twist Biosciences) with overhangs for cloning into a yeast display vector. DNA inserts and digested vectors were transformed into yeast, and the full plasmid was generated *in vivo* by homologous recombination(*42*). Cells were induced for scFv expression and displayed on the yeast cell surface(*42*). Induced cells were stained for binding to biotinylated SARS-CoV-2 receptor binding domain (RBD) antigen (Acro Biosystems) or SARS-CoV RBD (Genscript) and anti-c-myc (Exalpha) for scFv expression. ScFv constructs demonstrating binding by yeast surface display were reformatted into full-length human immunoglobulin 1 (IgG1) by subcloning the variable heavy (VH) chain and variable light (VL) chains into mammalian expression vectors containing a CMV promoter sequence, signal peptide, and corresponding constant regions using the Gibson cloning method (Codex DNA). Purified DNA was transfected into ExpiCHO cells following the manufacturer's recommended methods (Thermo Fisher Scientific). Following purification with MabSelect Sure Protein A affinity resin (Cytiva) the antibodies were further purified by size exclusion chromatography using Superdex200 resin (Cytiva) following the manufacturer's recommended methods. One antibody derived from llama immunized with prefusion stabilized SARS-CoV-1 with cross-reactivity to SARS-CoV-2 was cloned into a single expression plasmid containing two copies of the VHH binding domain linked in tandem, which in turn was linked to a tetramerization domain as described previously(*43*). Simple expression of the binding domains from this monomeric building block constructed following transfection of 293 cells allowed for the expression and assembly of soluble secreted protein as stable tetramers. One antibody was derived from Retained Display (ReD) scFv libraries in a screen against the SARS-CoV-2 S protein RBD domain(*44*). The scFv was reformatted to a diabody with a 6x-His fusion. The diabody was overexpressed in *E. coli* and subsequently purified by ni-NTA and size exclusion chromatography. The purity was assessed by SDS-PAGE and aggregation status was determined by HPLC-size exclusion chromatography (SEC) with a column having a 300 Å pore size. One set of antibodies was identified by Sanger sequencing phage at the conclusion of panning rounds. Variable heavy and variable light regions were reformatted and synthesized as IgG or VHH-Fc and transfected in Expi293 cells (ThermoFisher A14524) at 2:1 heavy to light chain ratio. Antibodies were purified with Phynexus Protein A column tips (Phynexus PTH-91-20-07). Another antibody was obtained from a de novo computationally optimized, fully human SuperHuman 2.0 discovery library (Distributed Bio) and was selected from a rapid screen of

billions of human antibodies for cross-reactivity to SARS-CoV-2. The antibodies were expressed from ExpiCHO cells. Two antibodies were isolated and purified as described(*45*). Two antibodies were generated by screening from Sanyoubio's fully human naïve antibody library (FHuNAL, Fab format, 4 x 10^{11} library size), fused with IgG1 Fc and expressed from ExpiCHO cells (Thermo Fisher Scientific). A further set of antibodies were derived from human immune repertoire mice immunized with SARS-CoV-2 Spike protein. Spike binding memory B cells were single cell sorted and the IgG H/L chains sequenced. Fully human IgG1 antibodies were synthesized from the sequences, expressed in suspension CHO cells. Other antibodies were isolated and purified as described previously (*46–51*).

High-throughput SPR epitope binning

Epitope binning was performed with a classical sandwich assay format on a Carterra LSA® HT-SPR instrument equipped with a CMDP sensor chip at 25 ˚C and in a HBSTE-BSA running buffer (10 mM HEPES pH 7.4, 150 mM NaCl, 3 mM EDTA, 0.05% Tween-20, supplemented with 0.5 mg/ml BSA). Two microfluidic modules, a 96-channel print-head (96PH) and a single flow cell (SFC), were used to deliver samples onto the sensor chip. Surface preparation was performed with 25 mM MES pH 5.5 with 0.05% Tween-20 as a running buffer. The chip was activated with a freshly prepared solution of 130 mM 1-ethyl-3-(3-dimethylaminopropyl)carbodiimide (EDC) + 33 mM N-hydroxysulfosuccinimide (Sulfo-NHS) in 0.1 M MES pH 5.5 using the SFC. Antibodies were immobilized using the 96PH for 10 minutes at 10 µg/mL diluted into 10 mM sodium acetate (pH 4.25). Unreactive esters were quenched with a 7-minute injection of 1 M ethanolamine-HCl (pH 8.5) using the SFC. The binning analysis was performed over this array with the HBSTE-BSA buffer as the running buffer and sample diluent. The RBD antigen was injected in each cycle for 4 minutes at 50 nM (1.8 µg/mL) and followed immediately by a 4-minute injection of the analyte antibody at 30 µg/mL (200 nM for IgG constructs). The surface was regenerated each cycle with double pulses (17 seconds per pulse) of 10 mM Glycine pH 2.0.

Data was processed and analyzed with Epitope® software (Carterra). Briefly, data was referenced using unprinted locations on the array and each binding cycle was normalized to the RBD capture level. The binding level of the analyte antibody just after the end of the injection was compared to that of a buffer alone injection. Signals that were significantly increased relative to the buffer controls are described as sandwiches and represent non-blocking behavior. Competition results were visualized as a heat map that depicts blocking relationships of analyte/ligand pairs. Clones

having similar patterns of competition are clustered together in a dendrogram and can be assigned to shared communities.

High-throughput SPR binding kinetics

The binding kinetics measurements of CoVIC antibody constructs were done on the Carterra LSA platform using HC30M sensor chips (Carterra) at 25 ˚C. Two microfluidic modules, a 96-channel print-head (96PH) and a single flow cell (SFC), were used to deliver liquids onto the sensor chip. In each assay, a single analyte was titrated against multiple CoVIC antibody constructs.

The immobilization of antibody constructs onto the HC30M chips depended on the antibody construct type. For monoclonal IgG antibodies, goat anti-Human IgG Fc secondary antibody was first immobilized onto the chip through amine-coupling. Briefly, the chip was first activated by 100 mM N-Hydroxysuccinimide (NHS) and 400 mM 1-Ethyl-3-(3-dimethylaminopropyl) carbodiimide hydrochloride (EDC) (GE healthcare, mixed 1:1:1 with 0.1 M MES buffer at pH 5.5) for 600 seconds, followed by immobilization of anti-Hu IgG Fc (in 10 mM Sodium Acetate at pH 4.5) at 50 μg/ml for 900 seconds. Unreactive esters were quenched with a 600-second injection of 1 M ethanolamine-HCl at pH 8.5. The chip was then exposed to double pulses (30 seconds per pulse) of 10 mM Glycine at pH 2.0. The CoVIC IgG antibodies were then captured by the anti-Hu IgG Fc at 5 μg/ml for 600 seconds using the 96PH, with 1X HBSTE buffer (10 mM HEPES pH 7.4, 150 mM NaCl, 3 mM EDTA and 0.01% Tween-20) as running buffer and antibody diluent. For other types of CoVIC antibody constructs (Fab, scFv, diabodies, etc.), the chip was activated by NHS/EDC for 600 seconds, followed by direct immobilization of these CoVIC antibody constructs (in 10 mM Sodium Acetate at pH 4.5) at multiple concentrations for 600 seconds using the 96PH. Unreactive esters were then quenched with a 600-second injection of 1 M ethanolamine-HCl at pH 8.5. Except for the capture of IgG by anti-Hu IgG Fc, the running buffer was 10 mM MES buffer at pH 5.5 with 0.01% Tween-20, and each CoVIC antibody construct at a given diluted concentration was immobilized onto 8 separate spots of the same chip, enabling replicating binding kinetics measurements. Unless specified above, the steps were done using the SFC.

A two-fold dilution series of the antigen was prepared in 1x HBSTE buffer. The top concentration for RBD, NTD and D614-HexaPro was respectively 40 μg/ml (1.11 μM), 320 μg/ml (5.71 μM) and 100 μg/ml (0.181 μM). A single antigen was used in each assay. The antigen at different concentrations was then injected using SFC onto the chip surface from the lowest to the highest concentration without regeneration, including several injections of buffer before the lowest non-zero concentration for signal stabilization. For each concentration, the data collection time-length for

baseline, association and dissociation were 120 seconds, 300 seconds and 900 seconds, respectively. For all assays the running buffer for titration was 1X HBSTE.

The titration data collected were first pre-processed in the NextGenKIT (Carterra) software, including reference subtraction, buffer subtraction and data smoothing. The data were then exported and analyzed using the TitrationAnalysis tool developed in-house (*52*). The RBD, NTD and D614-HexaPro binding time courses for each antibody construct immobilized on different spots were fitted to a 1:1 Langmuir model to derive ka, kd and KD values. In case of D614-HexaPro, due to its trimeric nature, the KD values derived using the 1:1 binding model fitting correspond to the avidity of the interaction with antibodies. For each CoVIC antibody construct – antigen pair, the best triplicate measurements satisfying the preset data acceptance criteria were selected and the averaged ka, kd and KD values are reported. The preset acceptance criteria for quality control included 1) standard error of the estimated ka, kd and KD in each replicate ≤20% and 2) fold change for all 3 parameters within the triplicate ≤ 3.

ACE-2 blocking assay

ACE2 blocking was measured using Biolayer Interferometry (BLI) on an Octet HTX instrument (Sartorius) by covalently immobilizing SARS-CoV-2 RBD and Human Serum Albumin (HSA) (reference to subtract response due to non-specific interactions) onto Amine Reactive 2nd Generation (AR2G) biosensors (Sartorius). The data was analyzed using Data Analysis HT 12.0 (CFR11) software (Sartorius). The biosensors were activated with a freshly prepared solution of EDC (1-Ethyl-3-[3-dimethylaminopropyl] carbodiimide hydrochloride) and s-NHS (N-hydroxysulfosuccinimide) in molecular biology grade water. RBD and HSA were diluted in 10 mM sodium acetate pH 5 buffer and immobilized onto 96 separate sensors to a loading density threshold not to exceed $\Delta\lambda = 0.7$ nm. Unreactive NHS esters on the surface of the sensors were quenched with 1M ethanolamine pH 8.5. Antibody and ACE2 binding were performed sequentially by dipping the RBD and HSA loaded biosensors into a well plate containing antibodies at 20 µg/ml followed by a solution of recombinant ACE2 (human IgGFc fused at 27.5 µg/ml for 5 minutes each. The diluent used for preparing antibodies and ACE2 solution was 1x kinetics buffer (Sartorius). ACE2 binding to immobilized RBD was monitored in real time in the absence and presence of antibodies pre-bound to RBD. The CoVIC reference mAbs CC12.3 and CC12.14 (*24*), and control SARS-CoV-2 Spike Neutralizing mAb (Sino Biological) were included in each experiment. ACE2 blocking assays for CoVIC 240-269 were performed using a recombinant double-strep tagged ACE2 construct after performing a bridging assay to ensure consistency in percent ACE2 blocking (for a select set of antibodies) between the two ACE2 constructs. The percent ACE2 blocking was

calculated as the percentage of decrease in ACE2 binding due to antibodies pre-bound to RBD compared with the ACE2 binding to RBD untreated with any antibody (1x kinetics buffer in place of antibody). All measurements were made in triplicate. The average of ACE2 binding to antibody untreated RBD was set as 0% blocking. The ACE2 blocking percentages shown for the CoVIC antibodies are the mean of triplicate measurements. In each assay, The SARS-CoV-2 Spike Neutralizing mAb (Sinobiological) was used as a positive control.

Preset data acceptance criterion for CV of triplicate measurements was <20% for antibodies %ACE2 blocking above the lower limit of detection (LLOD) of 13%. The LLOD was determined empirically using an Influenza Hemagglutinin specific monoclonal antibody CH65 that does not show reactivity for RBD. ACE2 blocking results were confirmed (for a subset of antibodies) with a second ACE2 blocking method utilizing a Meso Scale discovery (MSD, Rockville, MD) assay with Sulfo-Tag conjugated ACE-2.

Negative-stain EM to define antibody binding area

Different antibody formats, including IgG, Fab, scFv and VHH, were used for EM study. Fabs were obtained using either IdeS (Promega) or papain (Sigma), and purified by ion exchange chromatography using a MonoQ column (GE). Fab (70 μg), VHH (50 μg), scFv (70 μg) or IgG (140 μg); were incubated with 140 μg purified HexaPro.D614G Spike ectodomain in TBS buffer overnight at room temperature. The final concentration for Spike or IgG in incubation solution was ~0.25 μg/μL. Spike-antibody complexes were purified by SEC with a Superdex 6 Increase 10/300 column (GE) and verified by SDS-PAGE. For each complex, 4 μL of sample (~0.02 mg/mL) was applied to a CF400-Cu negative-stain grid (Electron Microscopy Sciences), and stained with 0.75% uranyl formate (Electron Microscopy Sciences). Between 50 and 400 micrographs were collected for each sample using a Titan Halo electron microscope (Thermo Fisher) and a Falcon 3EC direct electron detector at the magnification of 58,000X. EM-map reconstruction was performed using CryoSPARC (*53*), and the maps were aligned and displayed using Chimera X (*54*). Specifically, models having different RBD status (One RBD up: PDB:7A94 (*39*); Two RBDs up: PDB:7DCX (*55*); and Three RBDs up: PDB:7K4N (*19*), were fitted into NS-EM maps for antibody binding area identification. The NS-EM maps are available in the EMD database (www.emdataresource.org) and access numbers are listed in Table S3.

Production of recombinant virions

Recombinant SARS-CoV-2-pseudotyped VSV-ΔG-GFP were generated by transfecting 293T cells with phCMV3-SARS-CoV-2 S using TransIT according to the manufacturer's instructions. At 24 hr post-transfection, cells were

washed 2x with OptiMEM and then infected with rVSV-G pseudotyped ΔG-GFP parent virus (VSV-G*ΔG-GFP) at MOI = 2 for 2 hours with rocking. The virus was then removed, and the cells were washed twice with OPTI-MEM containing 2% FBS (OPTI-2) before addition of fresh OPTI-2. Supernatants containing rVSV-SARS-2 were removed 24 hours post-infection and clarified by centrifugation.

Viral titrations

Vero cells were seeded in 96-well plates at a sufficient density to produce a monolayer at the time of infection. Then, 10-fold serial dilutions of pseudovirus were made and added to cells in triplicate wells. Infection was allowed to proceed for 16-18 hr at 37 ˚C before fixation of the cells with 4% PFA and staining with Hoechst (10 µg/mL) in PBS. Fixative/stain was replaced with PBS and pseudovirus titers were quantified as the number of GFP-positive cells (fluorescent forming units, ffu/mL) using a CellInsight CX5 imager (ThermoScientific) and automated enumeration of cells expressing GFP.

Neutralization Assay

Pre-titrated amounts of rVSV-SARS-CoV-2 were incubated with serially diluted monoclonal antibodies at 37 °C for 1 hr before addition to confluent Vero (ATCC CCL-81) monolayers in 96-well plates. Infection proceeded for 16-18 hrs at 37 °C in 5% CO_2 before cells were fixed in 4% paraformaldehyde and stained with 10 µg/mL Hoechst. Cells were imaged using a CellInsight CX5 imager and infection was quantified by automated enumeration of total cells and those expressing GFP. Infection was normalized to the average number of cells infected with rVSV-SARS-CoV-2 incubated with human IgG isotype control. Data are presented as the relative infection for each antibody concentration. Neutralization IC_{50} titers were calculated using "One-Site Fit LogIC_{50}" regression in GraphPad Prism 9.0. Precision and accuracy of the Saphire lab pseudovirus neutralization assay were evaluated in the SARS-CoV-2 neutralization assay concordance survey (SNACS), ranking among the highest for specificity, precision and accuracy.

A

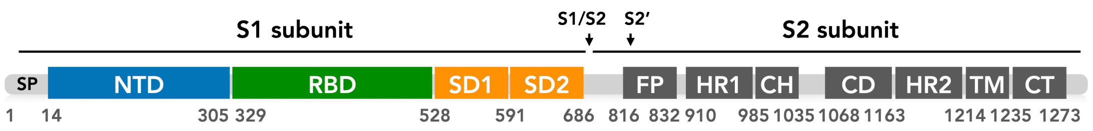

B

					SP		NTD																					
	Variant	WHO Label	Pango Lineage	GISAID	L5	S13	L18	T19	T20	P26	A67	HV 69-70	GT 75-76	D80	T95	D138	Y144	G142	W152	E154	FR 157 -158	R190	D215	A222	LAL 242 -244	R246	D253	G261
✤	VOC	Alpha	B.1.1.7	GRY								Δ					Δ											
✤	VOC	Beta	B.1.351	GH/501Y.V2			F							A									G		Δ	I		
✤	VOC	Gamma	P.1	GR/501Y.V3			F		N	S						Y						S						
✤	VOC	Delta	B.1.617.2	G/478K.V1				R													Δ							
	VOI	Epsilon	B.1.427	GH/452R.V1																								
✤	VOI	Epsilon	B.1.429	GH/452R.V1		I													C									
	VOI	Zeta	P.2	GR/484K.V2																								
	VOI	Eta	B.1.525	G/484K.V3							V	Δ					Δ											
	VOI	Theta	P.3	GR/1092K.V1																								
	VOI	Iota	B.1.526	GH/253G.V1	F										I												G	
	VOI	Kappa	B.1.617.1	G/452R.V3														D		K								
	VOI	Lambda	C.37	GR/452Q.V1									VI												RSYLTPGD246-253N			
	Mink-associated mutations											Δ																D
✤	4xM pseudovariant																											D
✤	5xM pseudovariant																											D
	Other high-frequency mutations																							V				

C

					RBD											SD1	SD2				
	Variant	WHO Label	Pango Lineage	GISAID	V367	K417	N439	L452	Y453	S477	T478	F486	E484	F490	N501	A570	D614	H655	Q677	P681	S2 mutations
✤	VOC	Alpha	B.1.1.7	GRY											Y	D	G			H	T716I/S982A/D1118H
✤	VOC	Beta	B.1.351	GH/501Y.V2		N							K		Y		G				A701V
✤	VOC	Gamma	P.1	GR/501Y.V3		T							K		Y		G	Y			T1027I/V1176F
✤	VOC	Delta	B.1.617.2	G/478K.V1				R			K						G			R	D950N
	VOI	Epsilon	B.1.427	GH/452R.V1				R									G				
✤	VOI	Epsilon	B.1.429	GH/452R.V1				R									G				
	VOI	Zeta	P.2	GR/484K.V2									K				G				V1176F
	VOI	Eta	B.1.525	G/484K.V3									K				G		H		F888L
	VOI	Theta	P.3	GR/ 1092K.V1									K		Y		G			H	E1092K
	VOI	Iota	B.1.526	GH/253G.V1									K				G				A701V
	VOI	Kappa	B.1.617.1	G/452R.V3				R					Q				G			R	Q1071H
	VOI	Lambda	C.37	GR/452Q.V1				Q						S			G				T859N
	Mink-associated mutations				F				F			L			T		G				
✤	4xM pseudovariant								F			L			T		G				
✤	5xM pseudovariant				F				F			L			T		G				
	Other high-frequency mutations						K			N											

Fig. S1

Figure S1. Emerging variants-related mutations on SARS-CoV-2 Spike. A. Schematic of SARS-CoV-2 Spike primary structure with subunit positions and domains. SP: signal peptide; NTD: N-terminal domain; RBD: receptor-binding domain; SD1: subdomain 1; SD2: subdomain 2; S1/S2: furin cleavage site; S2': S2 sub-cleavage site; FP: fusion peptide; CH: central helix HR1: heptad repeat 1; CD: connector domain; HR2: heptad repeat 2; TM: transmembrane domain; CT: cytoplasmic tail. **B.** Positions and substitutions of variant-related mutations in SP and NTD. **C.** Positions and substitutions of variant-related mutations in RBD, SD1, SD2 and S2 subunit. Variants of Concern (VOC) and Variants of Interest (VOI) are identified and updated (July 1st, 2021) by the World Health Organization (www.who.int/en/activities/tracking-SARS-CoV-2-variants). WHO Label, Pango lineage (cov-lineages.org) and GISAID clade/lineage (gisaid.org) for each variant is shown. The mutations and deletions are compared to the original Wuhan strain (GenBank: MN908947.3). The full-variants and mink-associated pseudovariants tested in the pseudovirus-based neutralization assay in this study are indicated by red stars, and single-point mutations and deletions (Δ) for neutralization testing are in red text.

Ab-Spike association

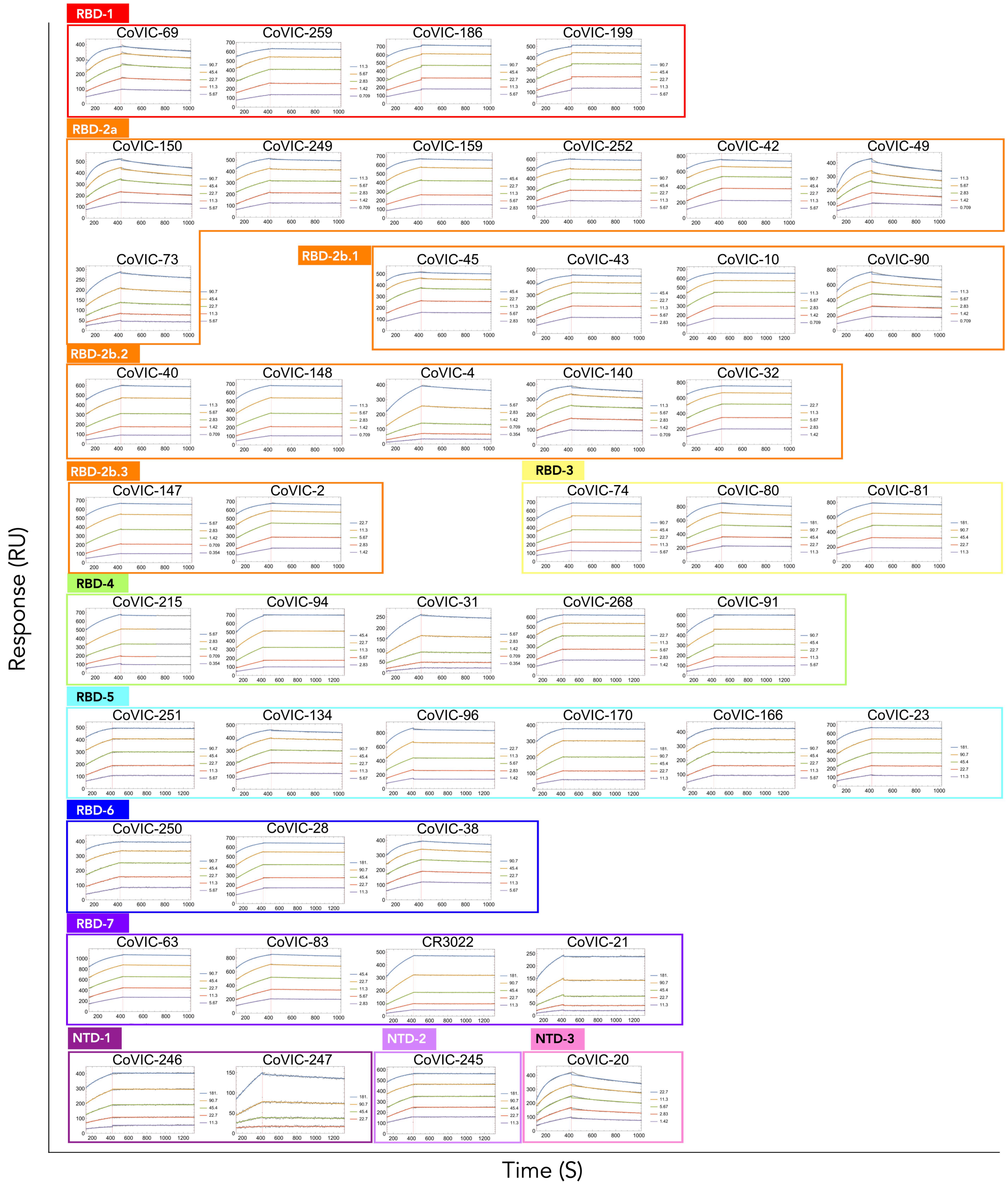

Fig. S2

Figure S2. Sensorgrams of Spike (D614-HexaPro) binding by a select number of CoVIC mAbs. Representative Spike-binding sensorgrams for a subset of CoVIC mAbs, grouped by community. Underlying data points are in gray, and colored lines were fitted using a Langmuir 1:1 binding model; D614 HexaPro concentrations (nM) selected for fitting are indicated. A red vertical line separates association data from dissociation data.

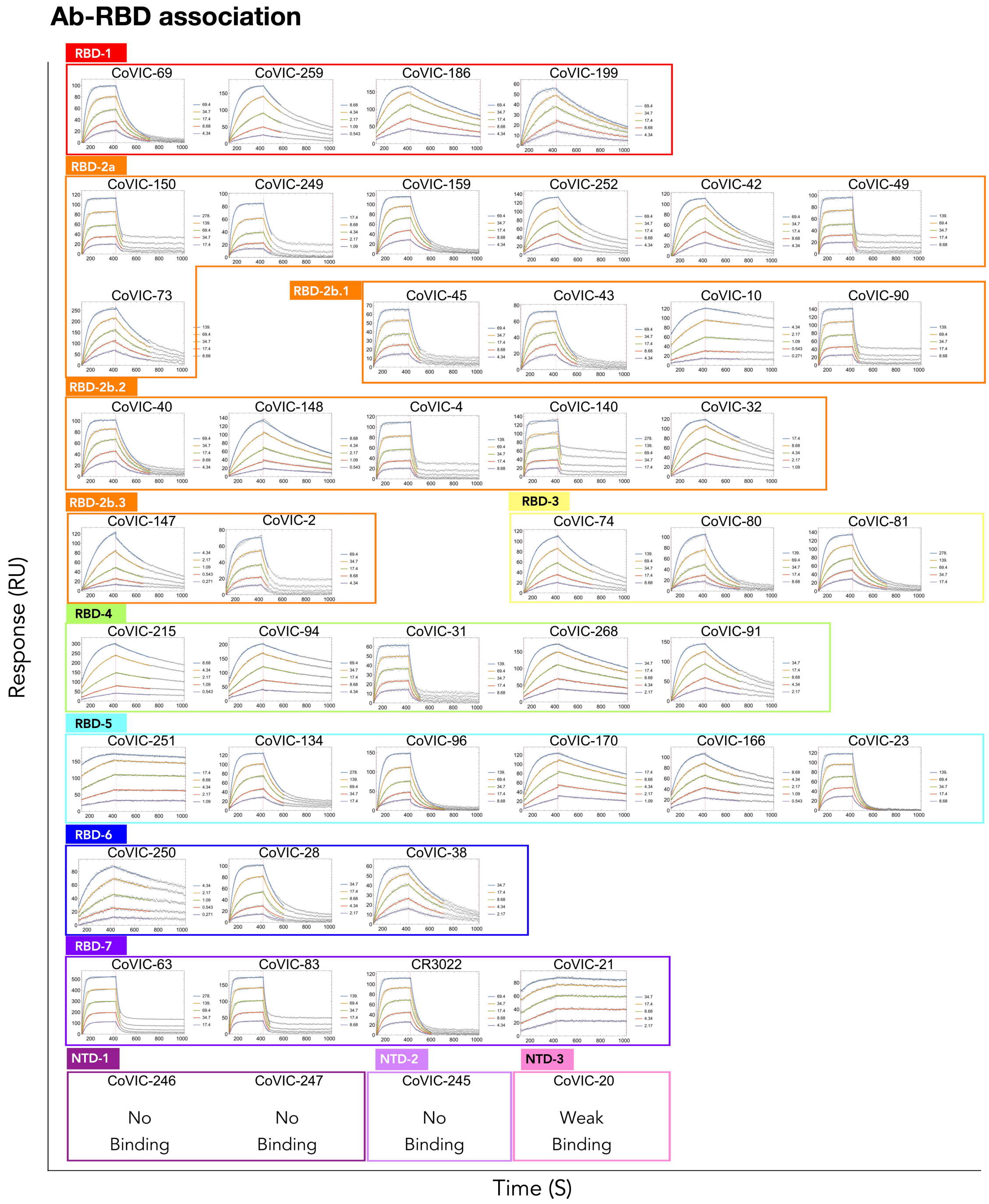
Ab-RBD association
Response (RU)
Time (S)
RBD-1
CoVIC-69
CoVIC-259
CoVIC-186
CoVIC-199
RBD-2a
CoVIC-150
CoVIC-249
CoVIC-159
CoVIC-252
CoVIC-42
CoVIC-49
CoVIC-73
RBD-2b.1
CoVIC-45
CoVIC-43
CoVIC-10
CoVIC-90
RBD-2b.2
CoVIC-40
CoVIC-148
CoVIC-4
CoVIC-140
CoVIC-32
RBD-2b.3
CoVIC-147
CoVIC-2
RBD-3
CoVIC-74
CoVIC-80
CoVIC-81
RBD-4
CoVIC-215
CoVIC-94
CoVIC-31
CoVIC-268
CoVIC-91
RBD-5
CoVIC-251
CoVIC-134
CoVIC-96
CoVIC-170
CoVIC-166
CoVIC-23
RBD-6
CoVIC-250
CoVIC-28
CoVIC-38
RBD-7
CoVIC-63
CoVIC-83
CR3022
CoVIC-21
NTD-1
CoVIC-246
No Binding
CoVIC-247
No Binding
NTD-2
CoVIC-245
No Binding
NTD-3
CoVIC-20
Weak Binding

Fig. S3

Figure S3. Sensorgrams of RBD binding by representative RBD-directed CoVIC mAbs. Representative RBD-binding sensorgrams for a subset of CoVIC mAbs grouped by community. Color schemes and fitting are as in Figure S2. If no or close to no binding or extremely weak (<30 RU) binding were observed, “No binding” and “Weak binding” is shown instead of the sensorgram.

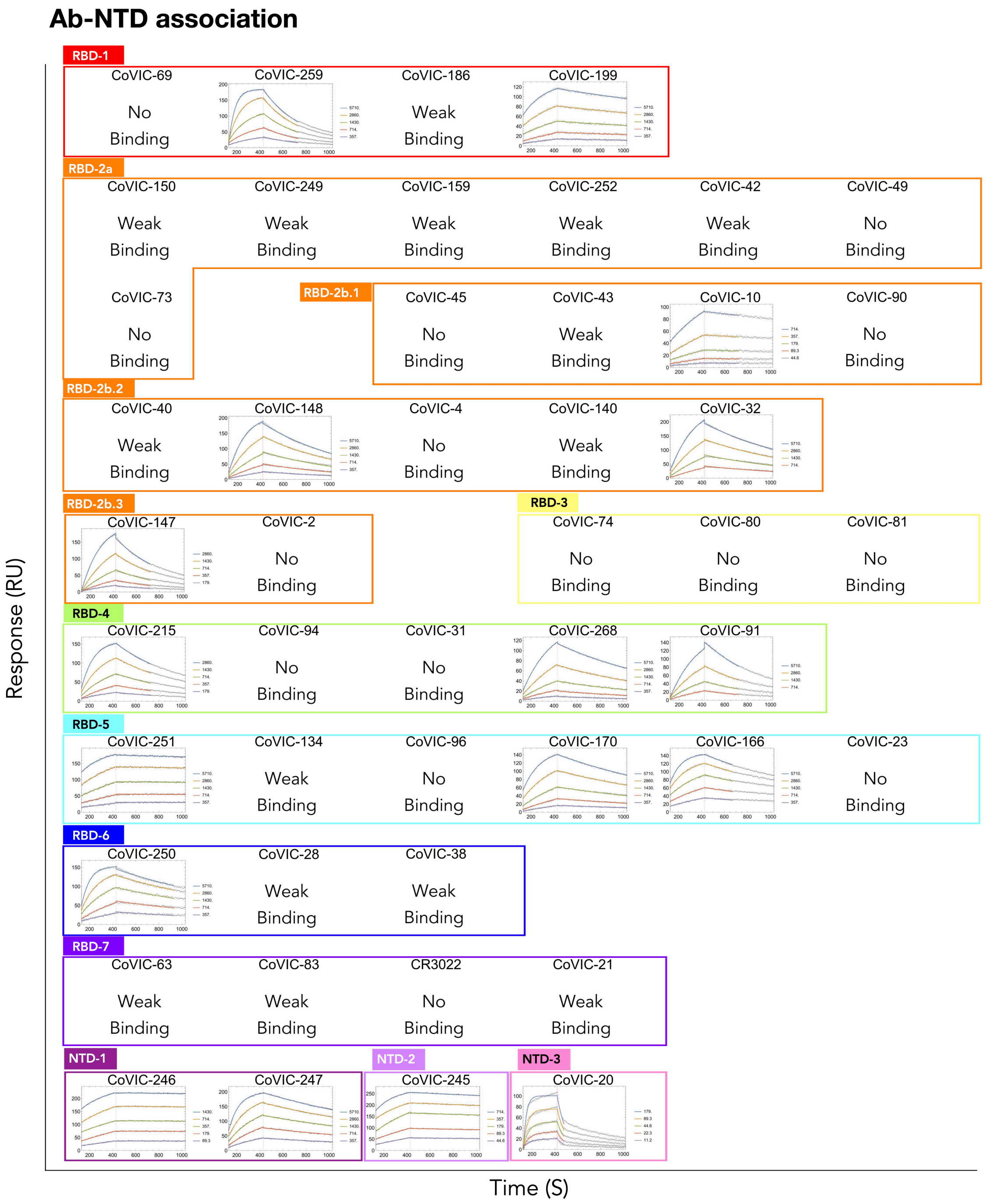
Ab-NTD association
RBD-1
CoVIC-69
No Binding
CoVIC-259
CoVIC-186
Weak Binding
CoVIC-199
RBD-2a
CoVIC-150
Weak Binding
CoVIC-249
Weak Binding
CoVIC-159
Weak Binding
CoVIC-252
Weak Binding
CoVIC-42
Weak Binding
CoVIC-49
No Binding
CoVIC-73
No Binding
RBD-2b.1
CoVIC-45
No Binding
CoVIC-43
Weak Binding
CoVIC-10
CoVIC-90
No Binding
RBD-2b.2
CoVIC-40
Weak Binding
CoVIC-148
CoVIC-4
No Binding
CoVIC-140
Weak Binding
CoVIC-32
RBD-2b.3
CoVIC-147
CoVIC-2
No Binding
RBD-3
CoVIC-74
No Binding
CoVIC-80
No Binding
CoVIC-81
No Binding
RBD-4
CoVIC-215
CoVIC-94
No Binding
CoVIC-31
No Binding
CoVIC-268
CoVIC-91
RBD-5
CoVIC-251
CoVIC-134
Weak Binding
CoVIC-96
No Binding
CoVIC-170
CoVIC-166
CoVIC-23
No Binding
RBD-6
CoVIC-250
CoVIC-28
Weak Binding
CoVIC-38
Weak Binding
RBD-7
CoVIC-63
Weak Binding
CoVIC-83
Weak Binding
CR3022
No Binding
CoVIC-21
Weak Binding
NTD-1
CoVIC-246
CoVIC-247
NTD-2
CoVIC-245
NTD-3
CoVIC-20
Response (RU)
Time (S)

Fig. S4

Figure S4. Sensorgrams of NTD binding by a select number of CoVIC mAbs. Representative NTD-binding sensorgrams for a subset of CoVIC mAbs grouped by community. Color schemes and fitting are as shown in Figure S2. “No binding” instead of a sensorgram appears if no binding or close to no binding was observed; “Weak binding” appears if a weak response appears (< 30 RU) or affinity (KD> 5.7 μM) was observed. The NTD binding shown for RBD community antibodies indicate the weak cross-reactive nature of these antibodies.

ACE2 blocking

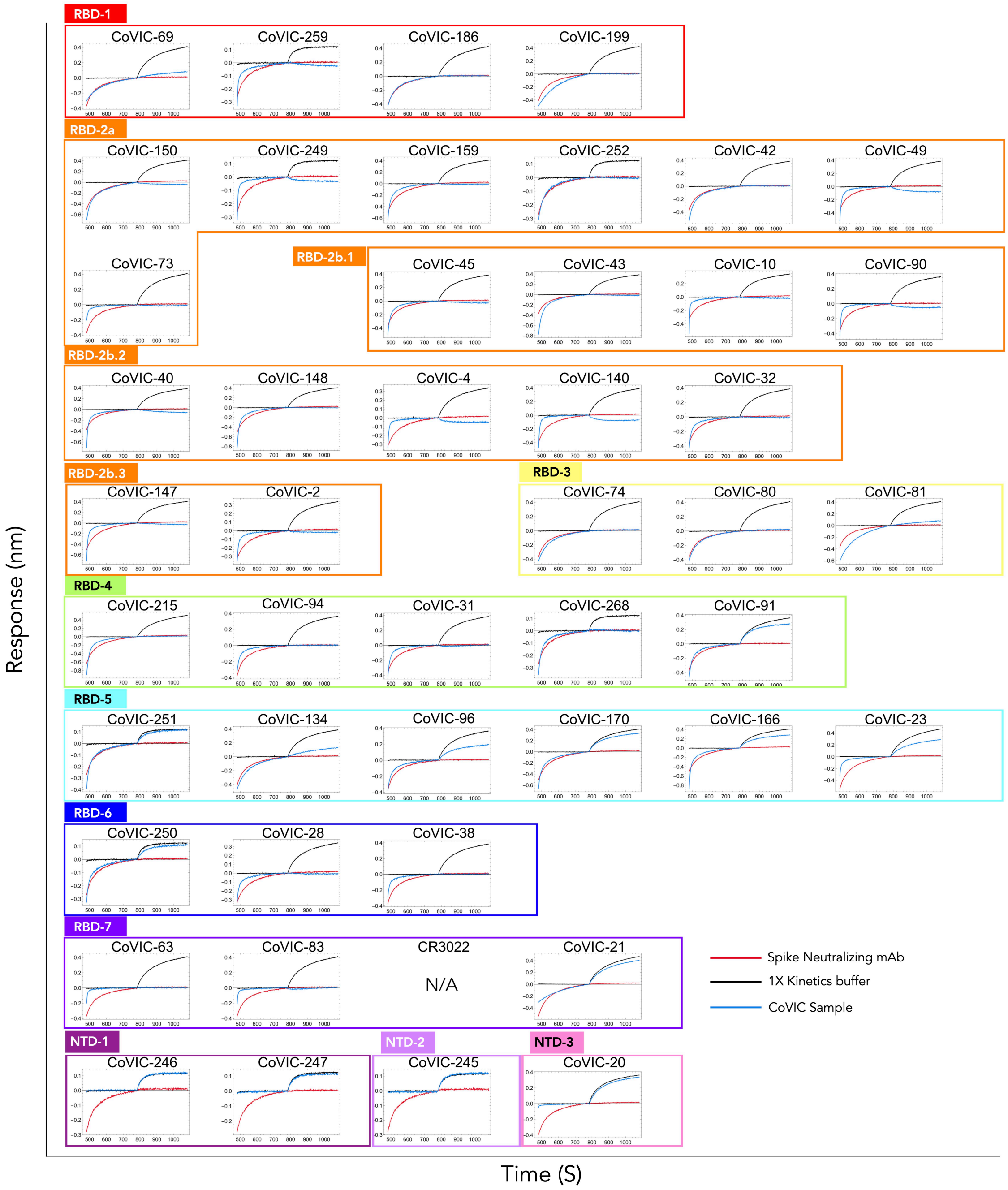

Fig. S5

Figure S5. ACE2 blocking assay sensorgrams. Representative BLI sensorgrams recorded to determine ACE2 blocking ability for a subset of CoVIC mAbs grouped by community. Each sensor was immobilized with RBD before initiating sensorgram acquisition. Each sensorgram consists of two steps, aligned at the beginning of the second step: binding of the CoVIC antibody construct, the positive control or kinetics buffer, followed by ACE-2 binding. In each sensorgram, the blue line corresponds to the CoVIC antibody construct in the first step, the red line corresponds to SARS-CoV-2 Spike neutralizing antibody (Sino Biological, positive control) in the first step and the black line corresponds to 1X kinetics buffer in the first step and is the average of triplicate measurements.

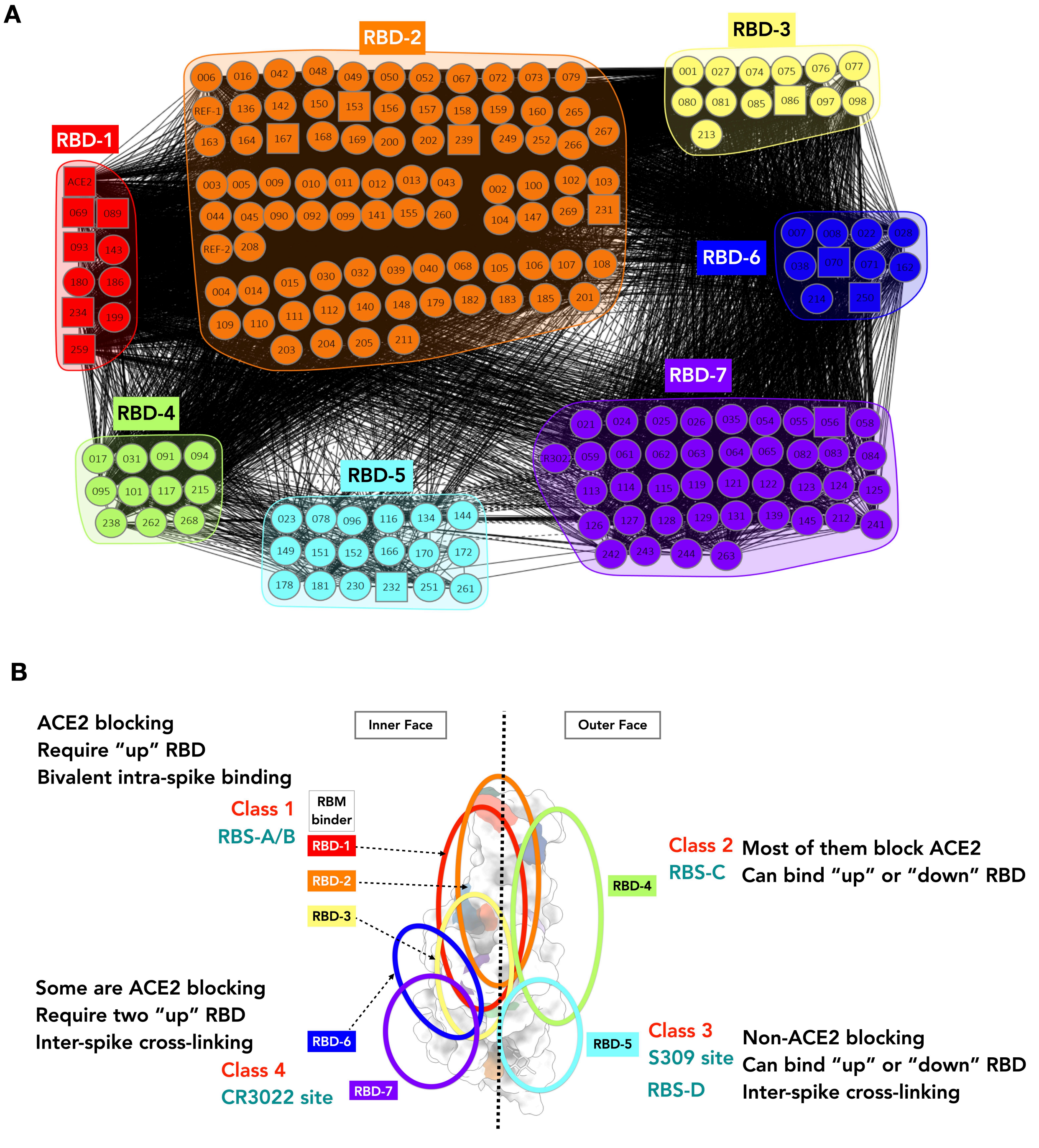
A
RBD-1
RBD-2
RBD-3
RBD-4
RBD-5
RBD-6
RBD-7
B
ACE2 blocking
Require "up" RBD
Bivalent intra-spike binding
Class 1
RBS-A/B
RBM binder
Inner Face
Outer Face
Class 2
RBS-C
Most of them block ACE2
Can bind "up" or "down" RBD
Some are ACE2 blocking
Require two "up" RBD
Inter-spike cross-linking
Class 4
CR3022 site
Class 3
S309 site
RBS-D
Non-ACE2 blocking
Can bind "up" or "down" RBD
Inter-spike cross-linking

Fig. S6

Figure S6. A. Community plot depicting competition relationships between CoVIC mAbs as determined by epitope binning using HT-SPR against SARS-CoV-2 Spike RBD. Colored nodes indicate individual mAbs. Circles and squares denote presence in the matrix either in both ligand and analyte directions or in only one orientation, respectively. Lines connecting nodes indicate that the two antibodies compete. The series of overlapping epitopes targeted by mAbs in the CoVIC panel created extensive competition between clusters. The dataset was analyzed by Carterra Epitope software to sort competition profiles of clones into related clusters, which are represented as shared colored regions. **B. Summary of the 7 RBD-directed communities based on antibody competition profiles and interpreted by previous epitope studies.** Ovals corresponding to the color scheme in Fig. 1 indicate binding epitopes of each community (model is adapted from PDB: 7A94 (*39*)). To facilitate comparison with antibodies outside of the CoVIC, compatibility of the CoVIC classification with previous classifications based on germline and structural information is shown (*10*, *16*). Communities RBD-1 through -3 (RBM binders): Class 1 or RBS-A/B; community RBD-4 (outer face binders): Class 2 or RBS-C; community RBD-5 (outer face binders): Class 3 or RBS-D/S309 site; community RBD-6 and -7 (inner face binders): Class 4 or CR3022 site. Information for ACE2 blocking and antibody binding modes of the communities is also indicated. Corresponding detailed information is provided in Table S1 and S3.

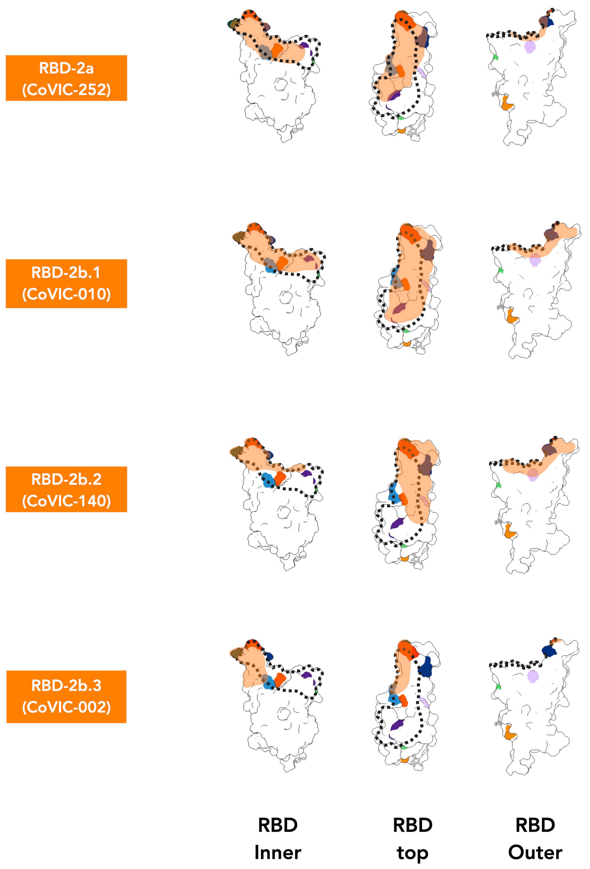
RBD-2a
(CoVIC-252)
RBD-2b.1
(CoVIC-010)
RBD-2b.2
(CoVIC-140)
RBD-2b.3
(CoVIC-002)
RBD
Inner
RBD
top
RBD
Outer

Fig. S7

Figure S7. RBD-2 mAbs bind across the top of the receptor binding motif (RBM). NS-EM footprints (colored orange) of representative antibodies from community RBD-2 are mapped onto an RBD monomer (adapted from PDB: 7A94(*39*)). The black dotted line indicates the RBM region.

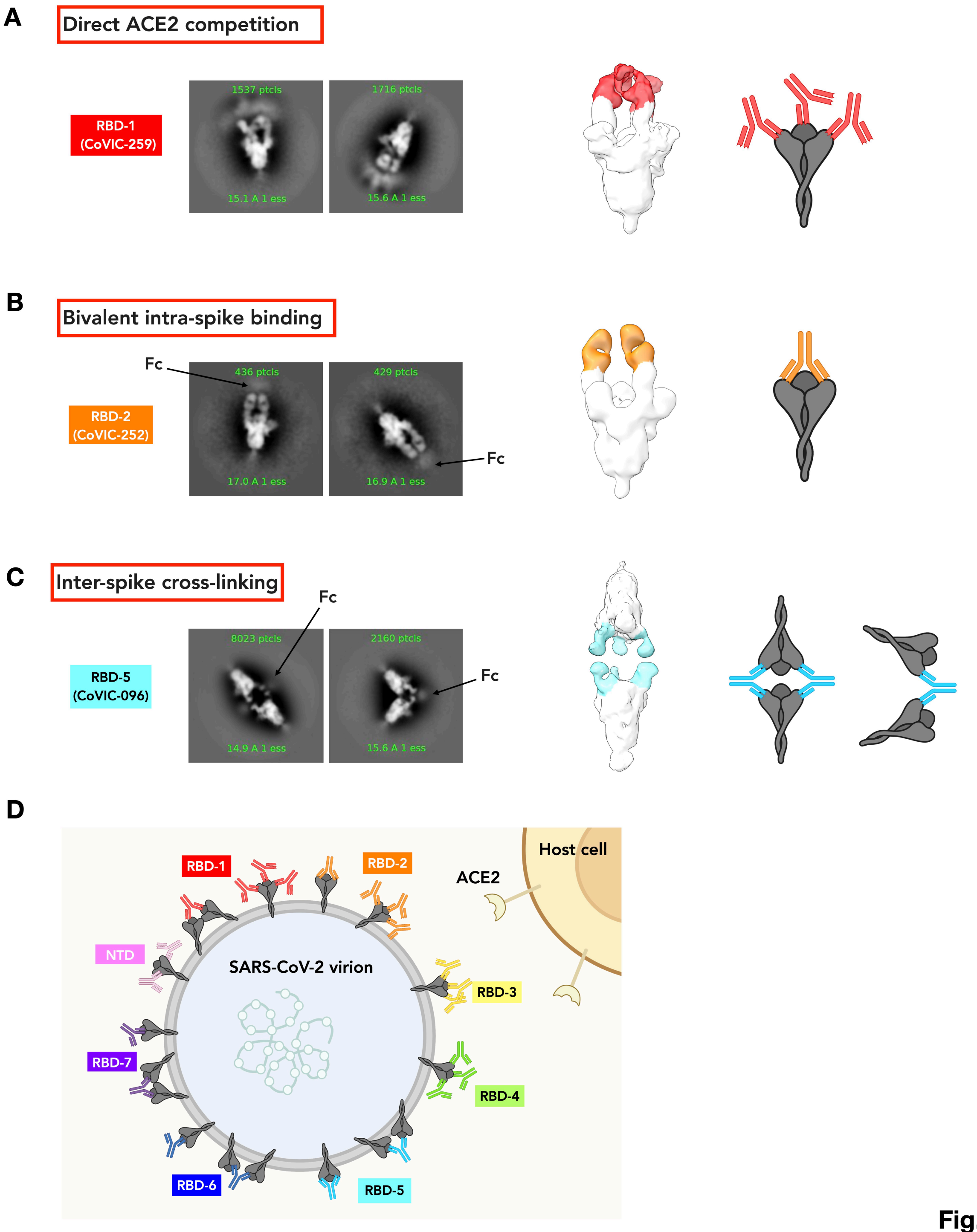
A
Direct ACE2 competition
RBD-1
(CoVIC-259)
1537 ptcls
15.1 A 1 ess
1716 ptcls
15.6 A 1 ess
B
Bivalent intra-spike binding
RBD-2
(CoVIC-252)
Fc
436 ptcls
17.0 A 1 ess
429 ptcls
16.9 A 1 ess
Fc
C
Inter-spike cross-linking
RBD-5
(CoVIC-096)
Fc
8023 ptcls
14.9 A 1 ess
2160 ptcls
15.6 A 1 ess
Fc
D
Host cell
ACE2
RBD-1
RBD-2
NTD
SARS-CoV-2 virion
RBD-3
RBD-7
RBD-4
RBD-6
RBD-5

Fig. S8

Figure S8. Three binding patterns for IgG bound to soluble Spike determined by NS-EM reveal potential neutralizing mechanisms. Particle-averaged 2D classes, 3D maps and cartoon illustrations of each binding pattern are shown. **A.** RBDs on Spikes are occupied by Fabs from IgGs in a binding mode that directly blocks interaction between Spike and ACE2 (e.g., CoVIC-259 from community RBD-1). **B.** Bivalent intra-Spike binding. Two RBDs on one Spike trimer are occupied by two Fabs from the same IgG (e.g., CoVIC-252 from community RBD-2). **C.** Inter-Spike crosslinking. Two Spike trimers are crosslinked by IgGs, which may sterically hinder ACE2 access (e.g., CoVIC-096 from community RBD-5). Specifically, there are two different crosslinking populations: 1) “head-to-head” Spikes crosslinked by 2 or 3 IgGs (this configuration was seen for the majority of particles) and 2) two “tilted” Spikes crosslinked by one IgG (see for the remainder of particles). **D.** Summary of possible IgG-Spike binding patterns for each RBD community.

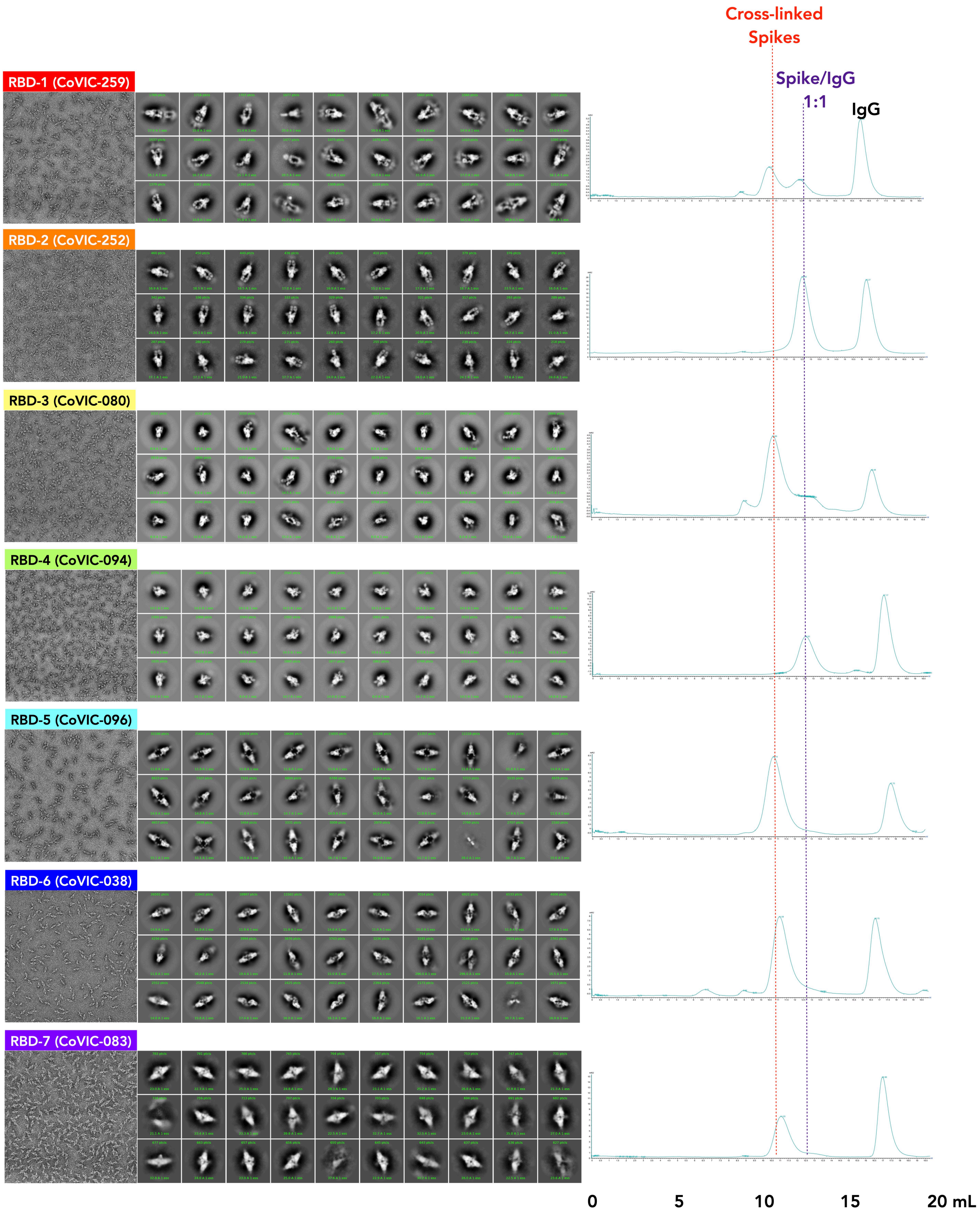

Cross-linked Spikes
Spike/IgG 1:1
IgG
RBD-1 (CoVIC-259)
RBD-2 (CoVIC-252)
RBD-3 (CoVIC-080)
RBD-4 (CoVIC-094)
RBD-5 (CoVIC-096)
RBD-6 (CoVIC-038)
RBD-7 (CoVIC-083)
0
5
10
15
20 mL

Fig. S9

Figure S9. Spike/IgG complexes with typical antibodies from each community. NS-EM micrographs and 2D-average classes are shown on the left, and the curves of size-exclusion chromatography (Superdex S6 Increase, GE) are on the right. Each complex was formed by incubating Spike and IgG at the same final concentrations (0.25 μg/μL Spike + 0.25 μg/μL IgG). Micrographs were taken at 58,000X magnification. The number of particles used to achieve each 2D-average class are shown in green with the averaged images. The red and purple dotted lines indicate positions of the peaks corresponding to cross-linked complexes and 1:1 ratio Spike/IgG complexes, respectively.

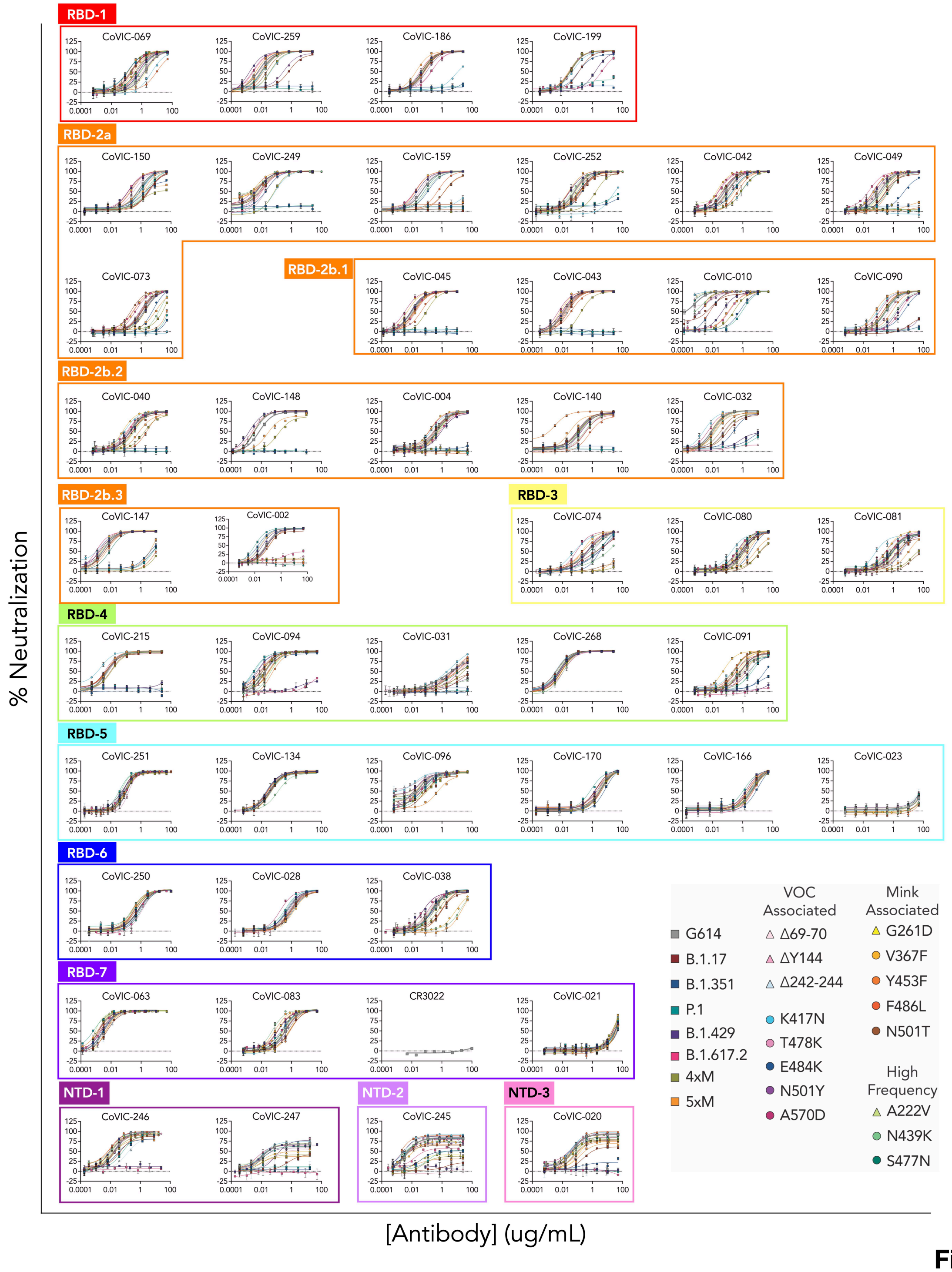

Fig. S10

369

370

371

Figure S10. Neutralization of pseudoviruses with emerging mutations. The community and antibody are indicated for each set of neutralization curves. Error bars indicate the standard deviation of technical duplicates, except for the parent G614 pseudovirus in which error bars indicate the standard deviation of at least two biological replicates (each performed in duplicate). IC_{50} values for each neutralization curve were calculated by nonlinear regression analysis and used in the fold-change analysis displayed in Fig. 3 and Table S4.

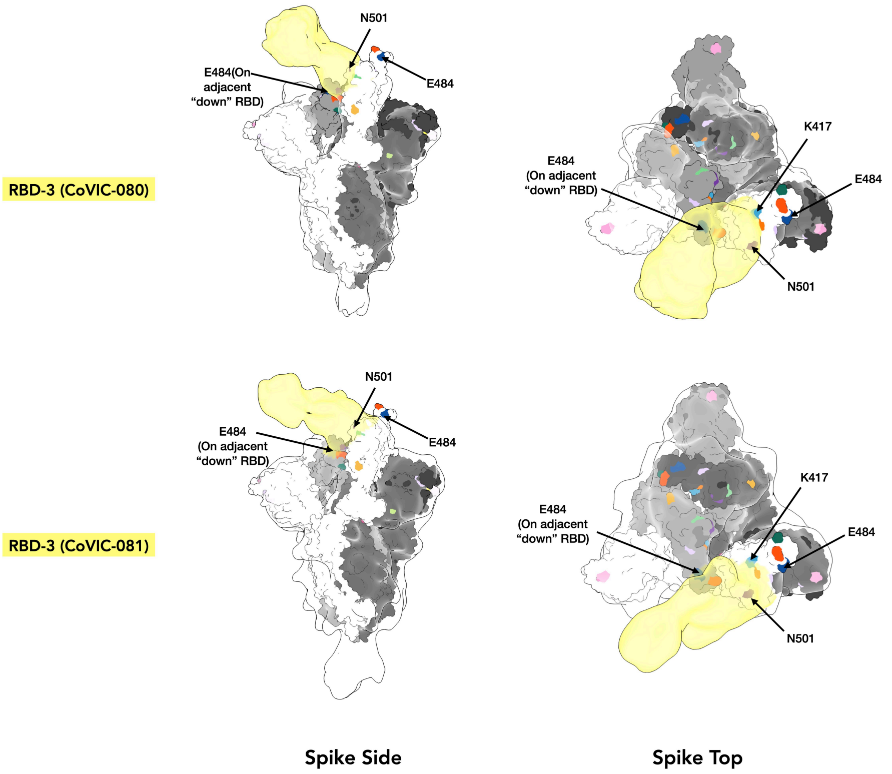
RBD-3 (CoVIC-080)
N501
E484(On adjacent “down” RBD)
E484
K417
E484 (On adjacent “down” RBD)
E484
N501
RBD-3 (CoVIC-081)
N501
E484 (On adjacent “down” RBD)
E484
K417
E484 (On adjacent “down” RBD)
E484
N501
Spike Side
Spike Top

Fig. S11

Figure S11. Footprints and binding pattern of RBD-3 antibodies. SARS-CoV-2 Spike model with one "up" RBD (PDB:7A94(*39*)) fits into the NS-EM maps of RBD-3 antibodies CoVIC-080 and -081. The antibody densities are colored in yellow. Residues that are mutated in VOCs are labeled. RBD-3 antibodies bind only to "up" RBDs, but approach at an angle that nears an adjacent "down" RBD. The neutralizing activity of RBD-3 is decreased by N501T/Y and E484K, and is increased by the K417N mutation. N501 and K417 are located within the footprints on the "up" RBD, whereas E484K from the adjacent "down" RBD may impact interaction between Spike and RBD-3 antibodies.

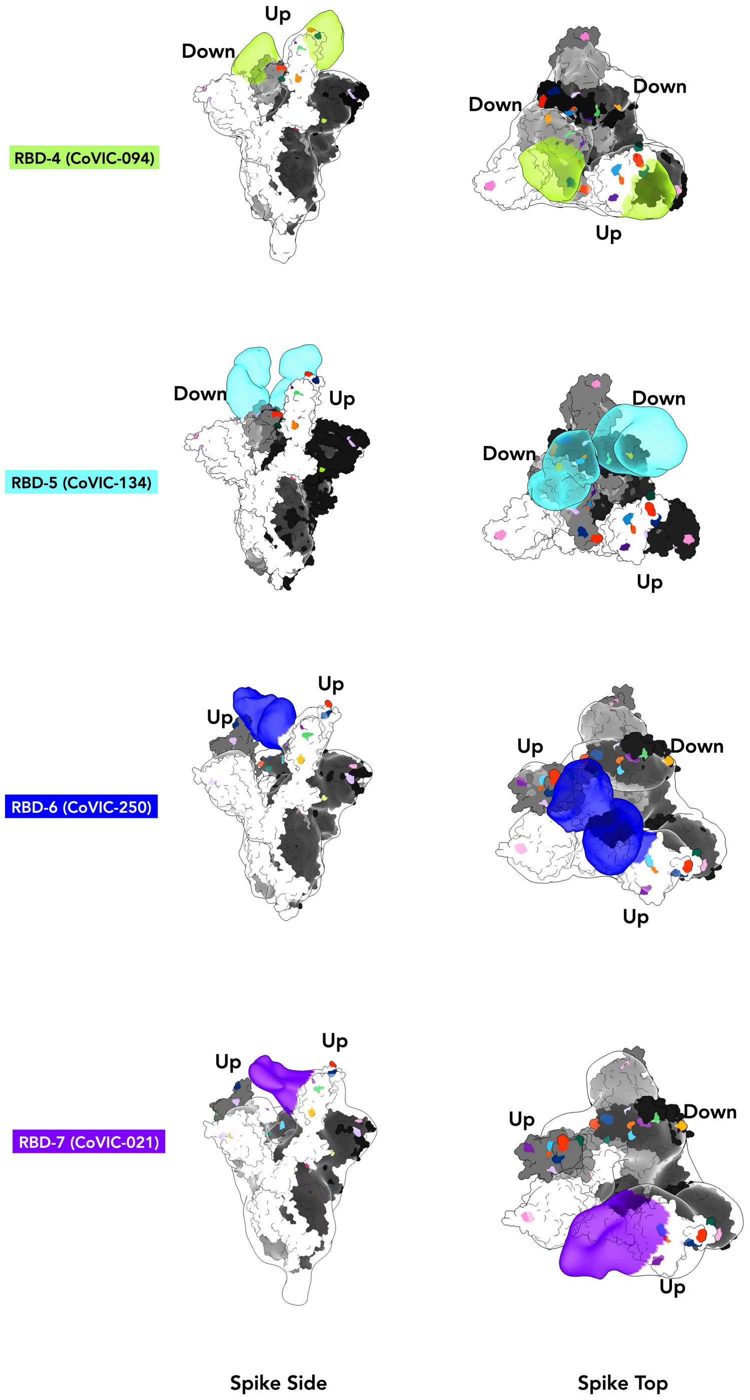
RBD-4 (CoVIC-094)
Up
Down
Down
Down
Up
RBD-5 (CoVIC-134)
Down
Up
Down
Down
Up
RBD-6 (CoVIC-250)
Up
Up
Up
Down
Up
RBD-7 (CoVIC-021)
Up
Up
Up
Down
Up
Spike Side
Spike Top

Fig. S12

Figure S12. RBD-binding profiles for select antibodies from communities RBD-4, RBD-5, RBD-6 and RBD-7. Representative antibodies from communities RBD-4 and -5 bind to the outer face of RBD, and can bind to either "up" or "down" RBDs. Antibodies from communities RBD-6 and -7 bind to the inner face of RBD and require both the binding and adjacent RBDs to be in the "up" conformation. Fabs are indicated by corresponding community colors. Docking models: PDB:7A94(*39*) (one RBD up); PDB:7DCX(*55*) (two RBDs up).

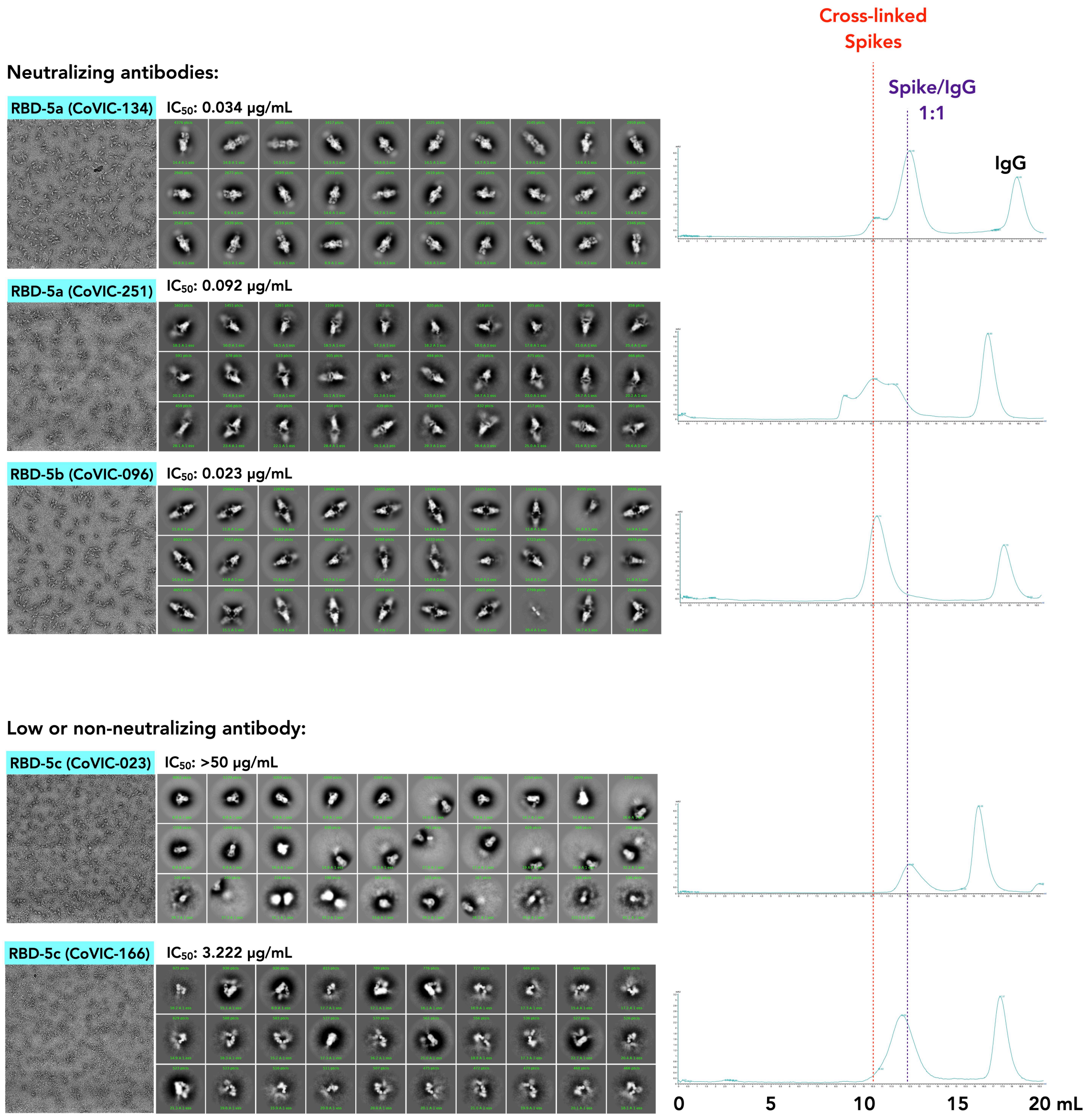
Cross-linked
Spikes
Neutralizing antibodies:
Spike/IgG
1:1
RBD-5a (CoVIC-134)
IC$_{50}$: 0.034 µg/mL
IgG
RBD-5a (CoVIC-251)
IC$_{50}$: 0.092 µg/mL
RBD-5b (CoVIC-096)
IC$_{50}$: 0.023 µg/mL
Low or non-neutralizing antibody:
RBD-5c (CoVIC-023)
IC$_{50}$: >50 µg/mL
RBD-5c (CoVIC-166)
IC$_{50}$: 3.222 µg/mL
0
5
10
15
20 mL

Fig. S13

Figure S13. Spike/IgG complexes with typical antibodies from community RBD-5. NS-EM micrographs and 2D-average classes are shown on the left, and the curves of size-exclusion chromatography (Superdex S6 Increase, GE) are on the right. Complex formation, image collection and chromatograph labels are the same as in Fig. S9.

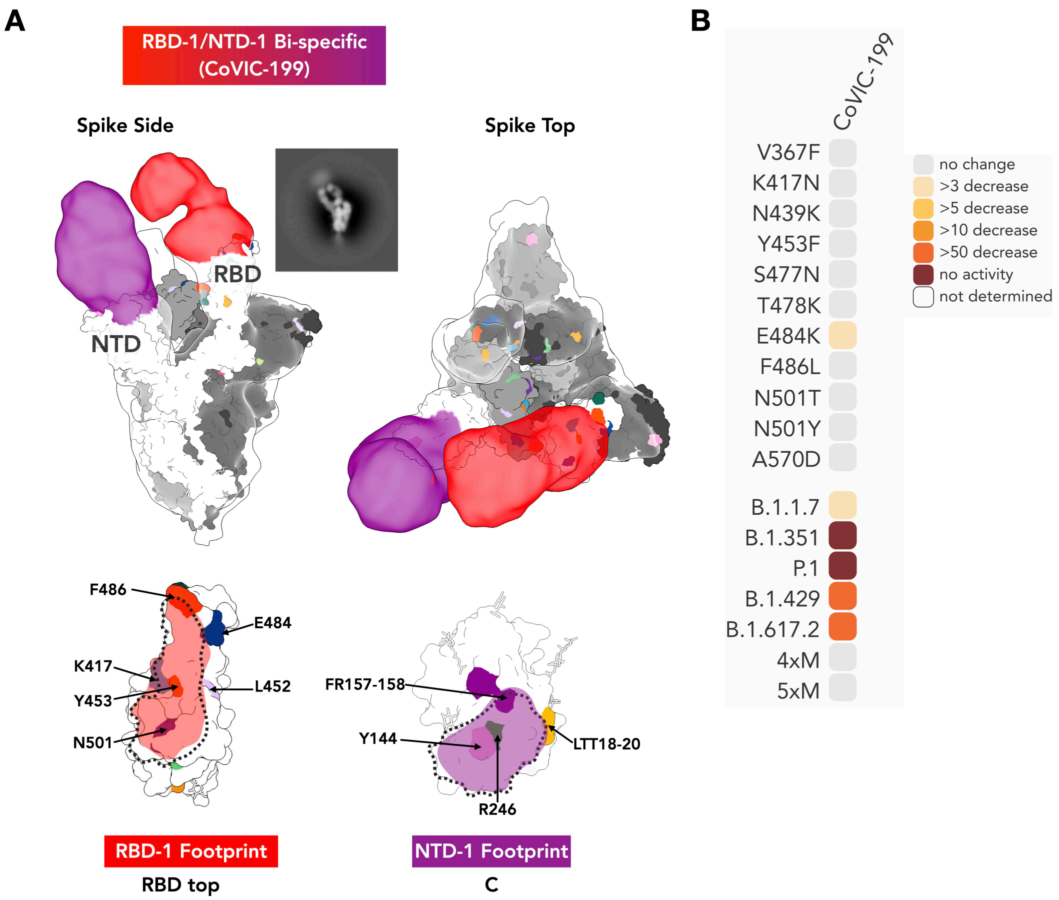
A
RBD-1/NTD-1 Bi-specific
(CoVIC-199)
Spike Side
Spike Top
RBD
NTD
F486
E484
K417
L452
Y453
N501
FR157-158
Y144
LTT18-20
R246
RBD-1 Footprint
RBD top
NTD-1 Footprint
C
B
CoVIC-199
V367F
K417N
N439K
Y453F
S477N
T478K
E484K
F486L
N501T
N501Y
A570D
B.1.1.7
B.1.351
P.1
B.1.429
B.1.617.2
4xM
5xM
no change
>3 decrease
>5 decrease
>10 decrease
>50 decrease
no activity
not determined

Fig. S14

Figure S14. NS-EM and neutralization of a bi-specific antibody. (A) NS-EM of CoVIC-199 demonstrates that this bi-specific antibody simultaneously engages the RBD-1 and NTD-1 epitopes. The ACE2 binding site and the NTD-supersite are outlined with a dotted line on the RBD and NTD, respectively. **(B)** Fold-change in potency of pseudovirus neutralization by CoVIC-199. Fig. S1 lists mutations present in each variant.

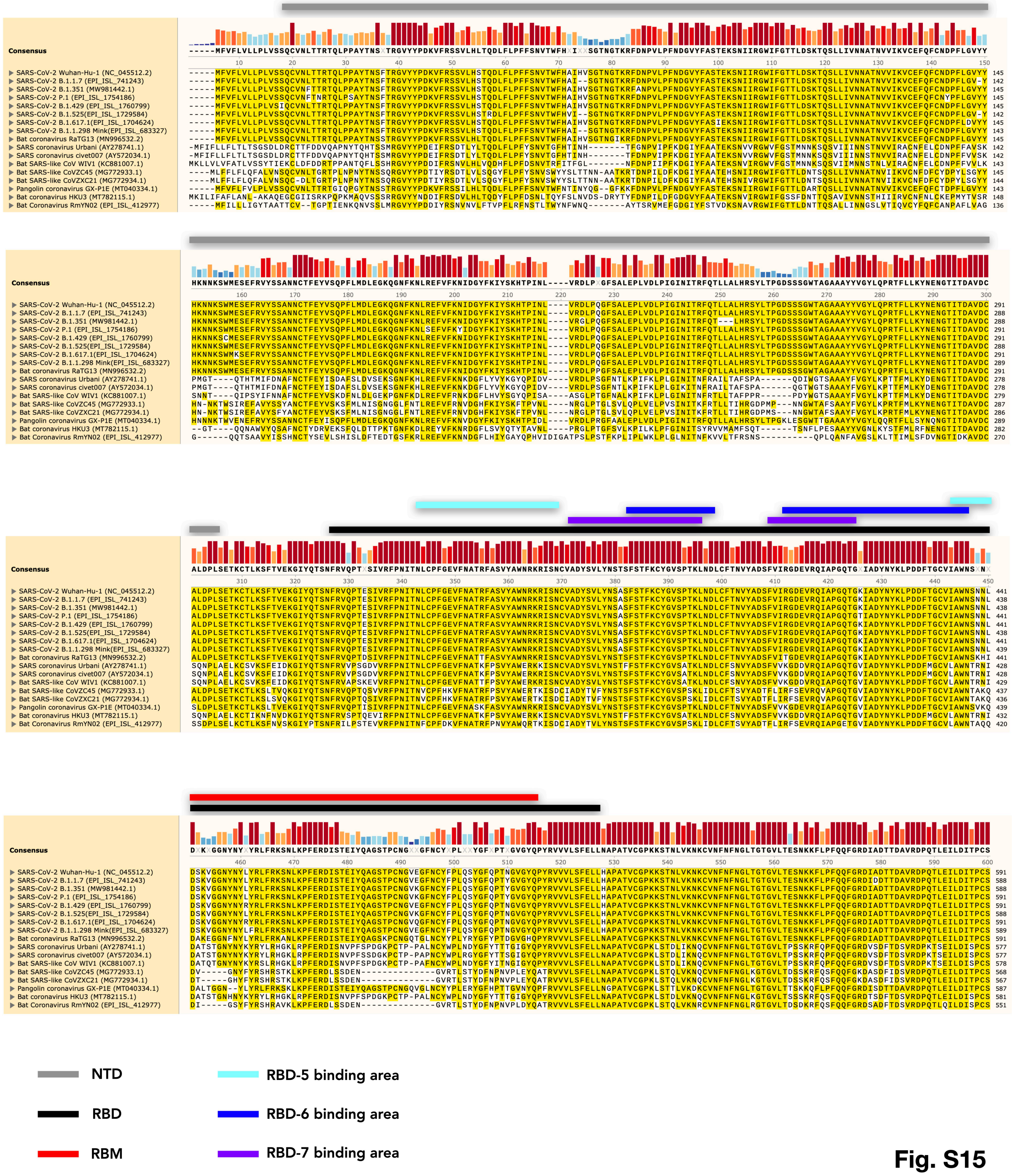
Consensus
SARS-CoV-2 Wuhan-Hu-1 (NC_045512.2)
SARS-CoV-2 B.1.1.7 (EPI_ISL_741243)
SARS-CoV-2 B.1.351 (MW981442.1)
SARS-CoV-2 P.1 (EPI_ISL_1754186)
SARS-CoV-2 B.1.429 (EPI_ISL_1760799)
SARS-CoV-2 B.1.525(EPI_ISL_1729584)
SARS-CoV-2 B.1.617.1(EPI_ISL_1704624)
SARS-CoV-2 B.1.1.298 Mink(EPI_ISL_683327)
Bat coronavirus RaTG13 (MN996532.2)
SARS coronavirus Urbani (AY278741.1)
SARS coronavirus civet007 (AY572034.1)
Bat SARS-like CoV WIV1 (KC881007.1)
Bat SARS-like CoVZC45 (MG772933.1)
Bat SARS-like CoVZXC21 (MG772934.1)
Pangolin coronavirus GX-P1E (MT040334.1)
Bat coronavirus HKU3 (MT782115.1)
Bat Coronavirus RmYN02 (EPI_ISL_412977)
NTD
RBD
RBM
RBD-5 binding area
RBD-6 binding area
RBD-7 binding area

Fig. S15

Figure S15. Alignment of S1 subunit protein sequences of typical *Sarbecoviruses*. VOCs of SARS-CoV-2, SARS-CoV-2 like bat virus (RaTG13), SARS-CoV and other SARS-like *Sarbecoviruses* were selected for the alignment using sequences obtained from GenBank or the GISAID database. Sequence alignment was carried out using Snapgene with the Clustal Omega method(*56*)). Conserved residues are highlighted in yellow. NTDs and RBMs are the two most variable regions among these *Sarbecoviruses*. Regions corresponding to the NTD, RBD and RBM are indicated by gray, black and red bars, respectively. The approximate binding areas of RBD-5, -6 and -7 antibodies are indicated by cyan, blue, and purple bars, respectively.

References and Notes

1. L. Piccoli, Y.-J. Park, M. A. Tortorici, N. Czudnochowski, A. C. Walls, M. Beltramello, C. Silacci-Fregni, D. Pinto, L. E. Rosen, J. E. Bowen, O. J. Acton, S. Jaconi, B. Guarino, A. Minola, F. Zatta, N. Sprugasci, J. Bassi, A. Peter, A. De Marco, J. C. Nix, F. Mele, S. Jovic, B. F. Rodriguez, S. V. Gupta, F. Jin, G. Piumatti, G. Lo Presti, A. F. Pellanda, M. Biggiogero, M. Tarkowski, M. S. Pizzuto, E. Cameroni, C. Havenar-Daughton, M. Smithey, D. Hong, V. Lepori, E. Albanese, A. Ceschi, E. Bernasconi, L. Elzi, P. Ferrari, C. Garzoni, A. Riva, G. Snell, F. Sallusto, K. Fink, H. W. Virgin, A. Lanzavecchia, D. Corti, D. Veesler, Mapping Neutralizing and Immunodominant Sites on the SARS-CoV-2 Spike Receptor-Binding Domain by Structure-Guided High-Resolution Serology. *Cell* **183**, 1024–1042.e21 (2020). doi:10.1016/j.cell.2020.09.037 Medline

2. B. Korber, W. M. Fischer, S. Gnanakaran, H. Yoon, J. Theiler, W. Abfalterer, N. Hengartner, E. E. Giorgi, T. Bhattacharya, B. Foley, K. M. Hastie, M. D. Parker, D. G. Partridge, C. M. Evans, T. M. Freeman, T. I. de Silva, C. McDanal, L. G. Perez, H. Tang, A. Moon-Walker, S. P. Whelan, C. C. LaBranche, E. O. Saphire, D. C. Montefiori; Sheffield COVID-19 Genomics Group, Tracking Changes in SARS-CoV-2 Spike: Evidence that D614G Increases Infectivity of the COVID-19 Virus. *Cell* **182**, 812–827.e19 (2020). doi:10.1016/j.cell.2020.06.043 Medline

3. E. C. Thomson, L. E. Rosen, J. G. Shepherd, R. Spreafico, A. da Silva Filipe, J. A. Wojcechowskyj, C. Davis, L. Piccoli, D. J. Pascall, J. Dillen, S. Lytras, N. Czudnochowski, R. Shah, M. Meury, N. Jesudason, A. De Marco, K. Li, J. Bassi, A. O'Toole, D. Pinto, R. M. Colquhoun, K. Culap, B. Jackson, F. Zatta, A. Rambaut, S. Jaconi, V. B. Sreenu, J. Nix, I. Zhang, R. F. Jarrett, W. G. Glass, M. Beltramello, K. Nomikou, M. Pizzuto, L. Tong, E. Cameroni, T. I. Croll, N. Johnson, J. Di Iulio, A. Wickenhagen, A. Ceschi, A. M. Harbison, D. Mair, P. Ferrari, K. Smollett, F. Sallusto, S. Carmichael, C. Garzoni, J. Nichols, M. Galli, J. Hughes, A. Riva, A. Ho, M. Schiuma, M. G. Semple, P. J. M. Openshaw, E. Fadda, J. K. Baillie, J. D. Chodera, S. J. Rihn, S. J. Lycett, H. W. Virgin, A. Telenti, D. Corti, D. L. Robertson, G. Snell; ISARIC4C Investigators; COVID-19 Genomics UK (COG-UK) Consortium, Circulating SARS-CoV-2 spike N439K variants maintain fitness while evading antibody-mediated immunity. *Cell* **184**, 1171–1187.e20 (2021). doi:10.1016/j.cell.2021.01.037 Medline

4. CDC, Science Brief: Emerging SARS-CoV-2 Variants (2021); www.cdc.gov/coronavirus/2019-ncov/more/science-and-research/scientific-brief-emerging-variants.html.

5. N. Oreshkova, R. J. Molenaar, S. Vreman, F. Harders, B. B. Oude Munnink, R. W. Hakze-van der Honing, N. Gerhards, P. Tolsma, R. Bouwstra, R. S. Sikkema, M. G. Tacken, M. M. de Rooij, E. Weesendorp, M. Y. Engelsma, C. J. Bruschke, L. A. Smit, M. Koopmans, W. H. van der Poel, A. Stegeman, SARS-CoV-2 infection in farmed minks, the Netherlands, April and May 2020. *Euro Surveill.* **25**, (2020). doi:10.2807/1560-7917.ES.2020.25.23.2001005 Medline

6. WHO, COVID-19 - Denmark (2020); www.who.int/emergencies/disease-outbreak-news/item/2020-DON301.

7. B. B. Oude Munnink, R. S. Sikkema, D. F. Nieuwenhuijse, R. J. Molenaar, E. Munger, R. Molenkamp, A. van der Spek, P. Tolsma, A. Rietveld, M. Brouwer, N. Bouwmeester-

Vincken, F. Harders, R. Hakze-van der Honing, M. C. A. Wegdam-Blans, R. J. Bouwstra, C. GeurtsvanKessel, A. A. van der Eijk, F. C. Velkers, L. A. M. Smit, A. Stegeman, W. H. M. van der Poel, M. P. G. Koopmans, Transmission of SARS-CoV-2 on mink farms between humans and mink and back to humans. *Science* **371**, 172–177 (2021). doi:10.1126/science.abe5901 Medline

8. L. van Dorp, C. C. S. Tan, S. D. Lam, D. Richard, C. Owen, D. Berchtold, C. Orengo, F. Balloux, Recurrent mutations in SARS-CoV-2 genomes isolated from mink point to rapid host-adaptation. bioRxiv 2020.11.16.384743 [Preprint](2020); https://doi.org/10.1101/2020.11.16.384743.

9. CDC, SARS-CoV-2 Variant Classifications and Definitions (2021); www.cdc.gov/coronavirus/2019-ncov/cases-updates/variant-surveillance/variant-info.html.

10. M. Yuan, D. Huang, C.-C. D. Lee, N. C. Wu, A. M. Jackson, X. Zhu, H. Liu, L. Peng, M. J. van Gils, R. W. Sanders, D. R. Burton, S. M. Reincke, H. Prüss, J. Kreye, D. Nemazee, A. B. Ward, I. A. Wilson, Structural and functional ramifications of antigenic drift in recent SARS-CoV-2 variants. *Science* **373**, 818–823 (2021). doi:10.1126/science.abh1139 Medline

11. J. Chen, R. Wang, M. Wang, G.-W. Wei, Mutations Strengthened SARS-CoV-2 Infectivity. *J. Mol. Biol.* **432**, 5212–5226 (2020). doi:10.1016/j.jmb.2020.07.009 Medline

12. E. B. Hodcroft, M. Zuber, S. Nadeau, T. G. Vaughan, K. H. D. Crawford, C. L. Althaus, M. L. Reichmuth, J. E. Bowen, A. C. Walls, D. Corti, J. D. Bloom, D. Veesler, D. Mateo, A. Hernando, I. Comas, F. González-Candelas, Spread of a SARS-CoV-2 variant through Europe in the summer of 2020. *Nature* **595**, 707–712 (2021). doi:10.1038/s41586-021-03677-y Medline

13. M. McCallum, J. Bassi, A. D. Marco, A. Chen, A. C. Walls, J. D. Iulio, M. A. Tortorici, M.-J. Navarro, C. Silacci-Fregni, C. Saliba, M. Agostini, D. Pinto, K. Culap, S. Bianchi, S. Jaconi, E. Cameroni, J. E. Bowen, S. W. Tilles, M. S. Pizzuto, S. B. Guastalla, G. Bona, A. F. Pellanda, C. Garzoni, W. C. Van Voorhis, L. E. Rosen, G. Snell, A. Telenti, H. W. Virgin, L. Piccoli, D. Corti, D. Veesler, SARS-CoV-2 immune evasion by the B.1.427/B.1.429 variant of concern. *Science* **373**, 648–654 (2021). doi:10.1126/science.abi7994 Medline

14. F. S. Collins, J. Woodcock, B. S. Graham, A. Arvin, P. Bieniasz, D. Ho, G. Alter, M. Nussenzweig, D. Burton, J. Tavel, S. L. Schendel, E. Ollmann Saphire, J. Dye, "Therapeutic Neutralizing Monoclonal Antibodies: Report of a Summit sponsored by Operation Warp Speed and the National Institutes of Health" (2020); www.nih.gov/sites/default/files/research-training/initiatives/activ/20200909-mAb-summit-pub.pdf.

15. Coronavirus Immunotherapy Consortium (2020); https://covic.lji.org/.

16. C. O. Barnes, C. A. Jette, M. E. Abernathy, K. A. Dam, S. R. Esswein, H. B. Gristick, A. G. Malyutin, N. G. Sharaf, K. E. Huey-Tubman, Y. E. Lee, D. F. Robbiani, M. C. Nussenzweig, A. P. West Jr., P. J. Bjorkman, SARS-CoV-2 neutralizing antibody structures inform therapeutic strategies. *Nature* **588**, 682–687 (2020). doi:10.1038/s41586-020-2852-1 Medline

17. J. Hansen, A. Baum, K. E. Pascal, V. Russo, S. Giordano, E. Wloga, B. O. Fulton, Y. Yan, K. Koon, K. Patel, K. M. Chung, A. Hermann, E. Ullman, J. Cruz, A. Rafique, T. Huang, J. Fairhurst, C. Libertiny, M. Malbec, W.-Y. Lee, R. Welsh, G. Farr, S. Pennington, D. Deshpande, J. Cheng, A. Watty, P. Bouffard, R. Babb, N. Levenkova, C. Chen, B. Zhang, A. Romero Hernandez, K. Saotome, Y. Zhou, M. Franklin, S. Sivapalasingam, D. C. Lye, S. Weston, J. Logue, R. Haupt, M. Frieman, G. Chen, W. Olson, A. J. Murphy, N. Stahl, G. D. Yancopoulos, C. A. Kyratsous, Studies in humanized mice and convalescent humans yield a SARS-CoV-2 antibody cocktail. *Science* **369**, 1010–1014 (2020). doi:10.1126/science.abd0827 Medline

18. N. C. Wu, M. Yuan, H. Liu, C. D. Lee, X. Zhu, S. Bangaru, J. L. Torres, T. G. Caniels, P. J. M. Brouwer, M. J. van Gils, R. W. Sanders, A. B. Ward, I. A. Wilson, An Alternative Binding Mode of IGHV3-53 Antibodies to the SARS-CoV-2 Receptor Binding Domain. *Cell Rep.* **33**, 108274 (2020). doi:10.1016/j.celrep.2020.108274 Medline

19. M. A. Tortorici, M. Beltramello, F. A. Lempp, D. Pinto, H. V. Dang, L. E. Rosen, M. McCallum, J. Bowen, A. Minola, S. Jaconi, F. Zatta, A. De Marco, B. Guarino, S. Bianchi, E. J. Lauron, H. Tucker, J. Zhou, A. Peter, C. Havenar-Daughton, J. A. Wojcechowskyj, J. B. Case, R. E. Chen, H. Kaiser, M. Montiel-Ruiz, M. Meury, N. Czudnochowski, R. Spreafico, J. Dillen, C. Ng, N. Sprugasci, K. Culap, F. Benigni, R. Abdelnabi, S. C. Foo, M. A. Schmid, E. Cameroni, A. Riva, A. Gabrieli, M. Galli, M. S. Pizzuto, J. Neyts, M. S. Diamond, H. W. Virgin, G. Snell, D. Corti, K. Fink, D. Veesler, Ultrapotent human antibodies protect against SARS-CoV-2 challenge via multiple mechanisms. *Science* **370**, 950–957 (2020). doi:10.1126/science.abe3354 Medline

20. A. Z. Wec, D. Wrapp, A. S. Herbert, D. P. Maurer, D. Haslwanter, M. Sakharkar, R. K. Jangra, M. E. Dieterle, A. Lilov, D. Huang, L. V. Tse, N. V. Johnson, C.-L. Hsieh, N. Wang, J. H. Nett, E. Champney, I. Burnina, M. Brown, S. Lin, M. Sinclair, C. Johnson, S. Pudi, R. Bortz 3rd, A. S. Wirchnianski, E. Laudermilch, C. Florez, J. M. Fels, C. M. O'Brien, B. S. Graham, D. Nemazee, D. R. Burton, R. S. Baric, J. E. Voss, K. Chandran, J. M. Dye, J. S. McLellan, L. M. Walker, Broad neutralization of SARS-related viruses by human monoclonal antibodies. *Science* **369**, 731–736 (2020). doi:10.1126/science.abc7424 Medline

21. T. Sztain, S.-H. Ahn, A. T. Bogetti, L. Casalino, J. A. Goldsmith, E. Seitz, R. S. McCool, F. L. Kearns, F. Acosta-Reyes, S. Maji, G. Mashayekhi, J. A. McCammon, A. Ourmazd, J. Frank, J. S. McLellan, L. T. Chong, R. E. Amaro, A glycan gate controls opening of the SARS-CoV-2 spike protein. *Nat. Chem.* **13**, 963–968 (2021). doi:10.1038/s41557-021-00758-3 Medline

22. H. Yao, Y. Song, Y. Chen, N. Wu, J. Xu, C. Sun, J. Zhang, T. Weng, Z. Zhang, Z. Wu, L. Cheng, D. Shi, X. Lu, J. Lei, M. Crispin, Y. Shi, L. Li, S. Li, Molecular Architecture of the SARS-CoV-2 Virus. *Cell* **183**, 730–738.e13 (2020). doi:10.1016/j.cell.2020.09.018 Medline

23. H. Liu, N. C. Wu, M. Yuan, S. Bangaru, J. L. Torres, T. G. Caniels, J. van Schooten, X. Zhu, C.-C. D. Lee, P. J. M. Brouwer, M. J. van Gils, R. W. Sanders, A. B. Ward, I. A. Wilson, Cross-neutralization of a SARS-CoV-2 antibody to a functionally conserved site is mediated by avidity. *Immunity* **53**, 1272–1280.e5 (2020). doi:10.1016/j.immuni.2020.10.023 Medline

24. M. Yuan, H. Liu, N. C. Wu, C. D. Lee, X. Zhu, F. Zhao, D. Huang, W. Yu, Y. Hua, H. Tien, T. F. Rogers, E. Landais, D. Sok, J. G. Jardine, D. R. Burton, I. A. Wilson, Structural basis of a shared antibody response to SARS-CoV-2. *Science* **369**, 1119–1123 (2020). doi:10.1126/science.abd2321 Medline

25. J. Huo, Y. Zhao, J. Ren, D. Zhou, H. M. E. Duyvesteyn, H. M. Ginn, L. Carrique, T. Malinauskas, R. R. Ruza, P. N. M. Shah, T. K. Tan, P. Rijal, N. Coombes, K. R. Bewley, J. A. Tree, J. Radecke, N. G. Paterson, P. Supasa, J. Mongkolsapaya, G. R. Screaton, M. Carroll, A. Townsend, E. E. Fry, R. J. Owens, D. I. Stuart, Neutralization of SARS-CoV-2 by Destruction of the Prefusion Spike. *Cell Host Microbe* **28**, 445–454.e6 (2020). doi:10.1016/j.chom.2020.06.010 Medline

26. M. McCallum, A. De Marco, F. A. Lempp, M. A. Tortorici, D. Pinto, A. C. Walls, M. Beltramello, A. Chen, Z. Liu, F. Zatta, S. Zepeda, J. di Iulio, J. E. Bowen, M. Montiel-Ruiz, J. Zhou, L. E. Rosen, S. Bianchi, B. Guarino, C. S. Fregni, R. Abdelnabi, S. C. Foo, P. W. Rothlauf, L.-M. Bloyet, F. Benigni, E. Cameroni, J. Neyts, A. Riva, G. Snell, A. Telenti, S. P. J. Whelan, H. W. Virgin, D. Corti, M. S. Pizzuto, D. Veesler, N-terminal domain antigenic mapping reveals a site of vulnerability for SARS-CoV-2. *Cell* **184**, 2332–2347.e16 (2021). doi:10.1016/j.cell.2021.03.028 Medline

27. X. Chi, R. Yan, J. Zhang, G. Zhang, Y. Zhang, M. Hao, Z. Zhang, P. Fan, Y. Dong, Y. Yang, Z. Chen, Y. Guo, J. Zhang, Y. Li, X. Song, Y. Chen, L. Xia, L. Fu, L. Hou, J. Xu, C. Yu, J. Li, Q. Zhou, W. Chen, A neutralizing human antibody binds to the N-terminal domain of the Spike protein of SARS-CoV-2. *Science* **369**, 650–655 (2020). doi:10.1126/science.abc6952 Medline

28. G. Cerutti, Y. Guo, T. Zhou, J. Gorman, M. Lee, M. Rapp, E. R Reddem, J. Yu, F. Bahna, J. Bimela, Y. Huang, P. S. Katsamba, L. Liu, M. S. Nair, R. Rawi, A. S. Olia, P. Wang, B. Zhang, G.-Y. Chuang, D. D. Ho, Z. Sheng, P. D. Kwong, L. Shapiro, Potent SARS-CoV-2 neutralizing antibodies directed against spike N-terminal domain target a single supersite. *Cell Host Microbe* **29**, 819–833.e7 (2021). doi:10.1016/j.chom.2021.03.005 Medline

29. M. McCallum, A. D. Marco, F. Lempp, M. A. Tortorici, D. Pinto, A. C. Walls, M. Beltramello, A. Chen, Z. Liu, F. Zatta, S. Zepeda, J. di Iulio, J. E. Bowen, M. Montiel-Ruiz, J. Zhou, L. E. Rosen, S. Bianchi, B. Guarino, C. S. Fregni, R. Abdelnabi, S.-Y. Caroline Foo, P. W. Rothlauf, L.-M. Bloyet, F. Benigni, E. Cameroni, J. Neyts, A. Riva, G. Snell, A. Telenti, S. P. J. Whelan, H. W. Virgin, D. Corti, M. S. Pizzuto, D. Veesler, N-terminal domain antigenic mapping reveals a site of vulnerability for SARS-CoV-2. bioRxiv 2021.01.14.426475 [Preprint] (2021); https://doi.org/10.1101/2021.01.14.426475.

30. Y. Cao, A. Yisimayi, Y. Bai, W. Huang, X. Li, Z. Zhang, T. Yuan, R. An, J. Wang, T. Xiao, S. Du, W. Ma, L. Song, Y. Li, X. Li, W. Song, J. Wu, S. Liu, X. Li, Y. Zhang, B. Su, X. Guo, Y. Wei, C. Gao, N. Zhang, Y. Zhang, Y. Dou, X. Xu, R. Shi, B. Lu, R. Jin, Y. Ma, C. Qin, Y. Wang, Y. Feng, J. Xiao, X. S. Xie, Humoral immune response to circulating SARS-CoV-2 variants elicited by inactivated and RBD-subunit vaccines. *Cell Res.* **31**, 732–741 (2021). doi:10.1038/s41422-021-00514-9 Medline

31. A. Baum, D. Ajithdoss, R. Copin, A. Zhou, K. Lanza, N. Negron, M. Ni, Y. Wei, K. Mohammadi, B. Musser, G. S. Atwal, A. Oyejide, Y. Goez-Gazi, J. Dutton, E.

Clemmons, H. M. Staples, C. Bartley, B. Klaffke, K. Alfson, M. Gazi, O. Gonzalez, E. Dick Jr., R. Carrion Jr., L. Pessaint, M. Porto, A. Cook, R. Brown, V. Ali, J. Greenhouse, T. Taylor, H. Andersen, M. G. Lewis, N. Stahl, A. J. Murphy, G. D. Yancopoulos, C. A. Kyratsous, REGN-COV2 antibodies prevent and treat SARS-CoV-2 infection in rhesus macaques and hamsters. *Science* **370**, 1110–1115 (2020). doi:10.1126/science.abe2402 Medline

32. NIH, Clinical trials of monoclonal antibodies to prevent COVID-19 now enrolling (2020); www.nih.gov/news-events/news-releases/clinical-trials-monoclonal-antibodies-prevent-covid-19-now-enrolling.

33. Z. Wang, F. Schmidt, Y. Weisblum, F. Muecksch, C. O. Barnes, S. Finkin, D. Schaefer-Babajew, M. Cipolla, C. Gaebler, J. A. Lieberman, T. Y. Oliveira, Z. Yang, M. E. Abernathy, K. E. Huey-Tubman, A. Hurley, M. Turroja, K. A. West, K. Gordon, K. G. Millard, V. Ramos, J. Da Silva, J. Xu, R. A. Colbert, R. Patel, J. Dizon, C. Unson-O'Brien, I. Shimeliovich, A. Gazumyan, M. Caskey, P. J. Bjorkman, R. Casellas, T. Hatziioannou, P. D. Bieniasz, M. C. Nussenzweig, mRNA vaccine-elicited antibodies to SARS-CoV-2 and circulating variants. *Nature* **592**, 616–622 (2021). doi:10.1038/s41586-021-03324-6 Medline

34. P. Wang, M. S. Nair, L. Liu, S. Iketani, Y. Luo, Y. Guo, M. Wang, J. Yu, B. Zhang, P. D. Kwong, B. S. Graham, J. R. Mascola, J. Y. Chang, M. T. Yin, M. Sobieszczyk, C. A. Kyratsous, L. Shapiro, Z. Sheng, Y. Huang, D. D. Ho, Antibody resistance of SARS-CoV-2 variants B.1.351 and B.1.1.7. *Nature* **593**, 130–135 (2021). doi:10.1038/s41586-021-03398-2 Medline

35. Y. Weisblum, F. Schmidt, F. Zhang, J. DaSilva, D. Poston, J. C. Lorenzi, F. Muecksch, M. Rutkowska, H.-H. Hoffmann, E. Michailidis, C. Gaebler, M. Agudelo, A. Cho, Z. Wang, A. Gazumyan, M. Cipolla, L. Luchsinger, C. D. Hillyer, M. Caskey, D. F. Robbiani, C. M. Rice, M. C. Nussenzweig, T. Hatziioannou, P. D. Bieniasz, Escape from neutralizing antibodies by SARS-CoV-2 spike protein variants. *eLife* **9**, e61312 (2020). doi:10.7554/eLife.61312 Medline

36. Z. Liu, L. A. VanBlargan, L.-M. Bloyet, P. W. Rothlauf, R. E. Chen, S. Stumpf, H. Zhao, J. M. Errico, E. S. Theel, M. J. Liebeskind, B. Alford, W. J. Buchser, A. H. Ellebedy, D. H. Fremont, M. S. Diamond, S. P. J. Whelan, Identification of SARS-CoV-2 spike mutations that attenuate monoclonal and serum antibody neutralization. *Cell Host Microbe* **29**, 477–488.e4 (2021). doi:10.1016/j.chom.2021.01.014 Medline

37. A. J. Greaney, T. N. Starr, P. Gilchuk, S. J. Zost, E. Binshtein, A. N. Loes, S. K. Hilton, J. Huddleston, R. Eguia, K. H. D. Crawford, A. S. Dingens, R. S. Nargi, R. E. Sutton, N. Suryadevara, P. W. Rothlauf, Z. Liu, S. P. J. Whelan, R. H. Carnahan, J. E. Crowe Jr., J. D. Bloom, Complete Mapping of Mutations to the SARS-CoV-2 Spike Receptor-Binding Domain that Escape Antibody Recognition. *Cell Host Microbe* **29**, 44–57.e9 (2021). doi:10.1016/j.chom.2020.11.007 Medline

38. S. A. Kemp, D. A. Collier, R. P. Datir, I. A. T. M. Ferreira, S. Gayed, A. Jahun, M. Hosmillo, C. Rees-Spear, P. Mlcochova, I. Ushiro Lumb, D. J. Roberts, A. Chandra, N. Temperton, The CITIID-NIHR BioResource COVID-19 Collaboration, The COVID-19 Genomics UK (COG-UK) Consortium, K. Sharrocks, E. Blane, Y. Modis, K. E. Leigh, J. A. G. Briggs, M. J. van Gils, K. G. C. Smith, J. R. Bradley, C. Smith, R. Doffinger, L.

Ceron-Gutierrez, G. Barcenas-Morales, D. D. Pollock, R. A. Goldstein, A. Smielewska, J. P. Skittrall, T. Gouliouris, I. G. Goodfellow, E. Gkrania-Klotsas, C. J. R. Illingworth, L. E. McCoy, R. K. Gupta, SARS-CoV-2 evolution during treatment of chronic infection. *Nature* **592**, 277–282 (2021). doi:10.1038/s41586-021-03291-y Medline

39. D. J. Benton, A. G. Wrobel, P. Xu, C. Roustan, S. R. Martin, P. B. Rosenthal, J. J. Skehel, S. J. Gamblin, Receptor binding and priming of the spike protein of SARS-CoV-2 for membrane fusion. *Nature* **588**, 327–330 (2020). doi:10.1038/s41586-020-2772-0 Medline

40. C.-L. Hsieh, J. A. Goldsmith, J. M. Schaub, A. M. DiVenere, H.-C. Kuo, K. Javanmardi, K. C. Le, D. Wrapp, A. G. Lee, Y. Liu, C.-W. Chou, P. O. Byrne, C. K. Hjorth, N. V. Johnson, J. Ludes-Meyers, A. W. Nguyen, J. Park, N. Wang, D. Amengor, J. J. Lavinder, G. C. Ippolito, J. A. Maynard, I. J. Finkelstein, J. S. McLellan, Structure-based design of prefusion-stabilized SARS-CoV-2 spikes. *Science* **369**, 1501–1505 (2020). doi:10.1126/science.abd0826 Medline

41. E. Seydoux, L. J. Homad, A. J. MacCamy, K. R. Parks, N. K. Hurlburt, M. F. Jennewein, N. R. Akins, A. B. Stuart, Y.-H. Wan, J. Feng, R. E. Whaley, S. Singh, M. Boeckh, K. W. Cohen, M. J. McElrath, J. A. Englund, H. Y. Chu, M. Pancera, A. T. McGuire, L. Stamatatos, Analysis of a SARS-CoV-2-Infected Individual Reveals Development of Potent Neutralizing Antibodies with Limited Somatic Mutation. *Immunity* **53**, 98–105.e5 (2020). doi:10.1016/j.immuni.2020.06.001 Medline

42. G. Chao, W. L. Lau, B. J. Hackel, S. L. Sazinsky, S. M. Lippow, K. D. Wittrup, Isolating and engineering human antibodies using yeast surface display. *Nat. Protoc.* **1**, 755–768 (2006). doi:10.1038/nprot.2006.94 Medline

43. A. Miller, S. Carr, T. Rabbitts, H. Ali, Multimeric antibodies with increased valency surpassing functional affinity and potency thresholds using novel formats. *mAbs* **12**, 1752529 (2020). doi:10.1080/19420862.2020.1752529 Medline

44. M. D. Beasley, K. P. Niven, W. R. Winnall, B. R. Kiefel, Bacterial cytoplasmic display platform Retained Display (ReD) identifies stable human germline antibody frameworks. *Biotechnol. J.* **10**, 783–789 (2015). doi:10.1002/biot.201400560 Medline

45. H. Yao, Y. Sun, Y.-Q. Deng, N. Wang, Y. Tan, N.-N. Zhang, X.-F. Li, C. Kong, Y.-P. Xu, Q. Chen, T.-S. Cao, H. Zhao, X. Yan, L. Cao, Z. Lv, D. Zhu, R. Feng, N. Wu, W. Zhang, Y. Hu, K. Chen, R.-R. Zhang, Q. Lv, S. Sun, Y. Zhou, R. Yan, G. Yang, X. Sun, C. Liu, X. Lu, L. Cheng, H. Qiu, X.-Y. Huang, T. Weng, D. Shi, W. Jiang, J. Shao, L. Wang, J. Zhang, T. Jiang, G. Lang, C.-F. Qin, L. Li, X. Wang, Rational development of a human antibody cocktail that deploys multiple functions to confer Pan-SARS-CoVs protection. *Cell Res.* **31**, 25–36 (2021). doi:10.1038/s41422-020-00444-y Medline

46. S. J. Zost, P. Gilchuk, J. B. Case, E. Binshtein, R. E. Chen, J. P. Nkolola, A. Schäfer, J. X. Reidy, A. Trivette, R. S. Nargi, R. E. Sutton, N. Suryadevara, D. R. Martinez, L. E. Williamson, E. C. Chen, T. Jones, S. Day, L. Myers, A. O. Hassan, N. M. Kafai, E. S. Winkler, J. M. Fox, S. Shrihari, B. K. Mueller, J. Meiler, A. Chandrashekar, N. B. Mercado, J. J. Steinhardt, K. Ren, Y.-M. Loo, N. L. Kallewaard, B. T. McCune, S. P. Keeler, M. J. Holtzman, D. H. Barouch, L. E. Gralinski, R. S. Baric, L. B. Thackray, M. S. Diamond, R. H. Carnahan, J. E. Crowe Jr., Potently neutralizing and protective human antibodies against SARS-CoV-2. *Nature* **584**, 443–449 (2020). doi:10.1038/s41586-020-2548-6 Medline

47. S. J. Zost, P. Gilchuk, R. E. Chen, J. B. Case, J. X. Reidy, A. Trivette, R. S. Nargi, R. E. Sutton, N. Suryadevara, E. C. Chen, E. Binshtein, S. Shrihari, M. Ostrowski, H. Y. Chu, J. E. Didier, K. W. MacRenaris, T. Jones, S. Day, L. Myers, F. Eun-Hyung Lee, D. C. Nguyen, I. Sanz, D. R. Martinez, P. W. Rothlauf, L.-M. Bloyet, S. P. J. Whelan, R. S. Baric, L. B. Thackray, M. S. Diamond, R. H. Carnahan, J. E. Crowe Jr., Rapid isolation and profiling of a diverse panel of human monoclonal antibodies targeting the SARS-CoV-2 spike protein. *Nat. Med.* **26**, 1422–1427 (2020). doi:10.1038/s41591-020-0998-x Medline

48. M. T. Tomic, Y. Espinoza, Z. Martinez, K. Pham, R. R. Cobb, D. M. Snow, C. G. Earnhart, T. Pals, E. S. Syar, N. Niemuth, D. J. Kobs, S. Farr-Jones, J. D. Marks, Monoclonal Antibody Combinations Prevent Serotype A and Serotype B Inhalational Botulism in a Guinea Pig Model. *Toxins* **11**, 208 (2019). doi:10.3390/toxins11040208 Medline

49. L. Liu, P. Wang, M. S. Nair, J. Yu, M. Rapp, Q. Wang, Y. Luo, J. F.-W. Chan, V. Sahi, A. Figueroa, X. V. Guo, G. Cerutti, J. Bimela, J. Gorman, T. Zhou, Z. Chen, K.-Y. Yuen, P. D. Kwong, J. G. Sodroski, M. T. Yin, Z. Sheng, Y. Huang, L. Shapiro, D. D. Ho, Potent neutralizing antibodies against multiple epitopes on SARS-CoV-2 spike. *Nature* **584**, 450–456 (2020). doi:10.1038/s41586-020-2571-7 Medline

50. J. Wan, S. Xing, L. Ding, Y. Wang, C. Gu, Y. Wu, B. Rong, C. Li, S. Wang, K. Chen, C. He, D. Zhu, S. Yuan, C. Qiu, C. Zhao, L. Nie, Z. Gao, J. Jiao, X. Zhang, X. Wang, T. Ying, H. Wang, Y. Xie, Y. Lu, J. Xu, F. Lan, Human-IgG-Neutralizing Monoclonal Antibodies Block the SARS-CoV-2 Infection. *Cell Rep.* **32**, 107918 (2020). doi:10.1016/j.celrep.2020.107918 Medline

51. W. Schaefer, J. T. Regula, M. Bähner, J. Schanzer, R. Croasdale, H. Dürr, C. Gassner, G. Georges, H. Kettenberger, S. Imhof-Jung, M. Schwaiger, K. G. Stubenrauch, C. Sustmann, M. Thomas, W. Scheuer, C. Klein, Immunoglobulin domain crossover as a generic approach for the production of bispecific IgG antibodies. *Proc. Natl. Acad. Sci. U.S.A.* **108**, 11187–11192 (2011). doi:10.1073/pnas.1019002108 Medline

52. K. Li, G. Q. Horn, S. M. Alam, G. D. Tomaras, S. M. Dennison, Titrationanalysis: A Tool for High-throughput Analysis of Binding Kinetics Data for Multiple Label-Free Platforms. *Biophys. J.* **120**, 265a–266a (2021). doi:10.1016/j.bpj.2020.11.1701

53. A. Punjani, J. L. Rubinstein, D. J. Fleet, M. A. Brubaker, cryoSPARC: Algorithms for rapid unsupervised cryo-EM structure determination. *Nat. Methods* **14**, 290–296 (2017). doi:10.1038/nmeth.4169 Medline

54. E. F. Pettersen, T. D. Goddard, C. C. Huang, E. C. Meng, G. S. Couch, T. I. Croll, J. H. Morris, T. E. Ferrin, UCSF ChimeraX: Structure visualization for researchers, educators, and developers. *Protein Sci.* **30**, 70–82 (2021). doi:10.1002/pro.3943 Medline

55. C. Zhang, Y. Wang, Y. Zhu, C. Liu, C. Gu, S. Xu, Y. Wang, Y. Zhou, Y. Wang, W. Han, X. Hong, Y. Yang, X. Zhang, T. Wang, C. Xu, Q. Hong, S. Wang, Q. Zhao, W. Qiao, J. Zang, L. Kong, F. Wang, H. Wang, D. Qu, D. Lavillette, H. Tang, Q. Deng, Y. Xie, Y. Cong, Z. Huang, Development and structural basis of a two-MAb cocktail for treating SARS-CoV-2 infections. *Nat. Commun.* **12**, 264 (2021). doi:10.1038/s41467-020-20465-w Medline

56. F. Sievers, A. Wilm, D. Dineen, T. J. Gibson, K. Karplus, W. Li, R. Lopez, H. McWilliam, M. Remmert, J. Söding, J. D. Thompson, D. G. Higgins, Fast, scalable generation of

high-quality protein multiple sequence alignments using Clustal Omega. *Mol. Syst. Biol.* **7**, 539 (2011). doi:10.1038/msb.2011.75 Medline
