# Supplementary material for: Defining variant-resistant epitopes targeted by SARS-CoV-2 antibodies: A global consortium study
Source: Science. 2021 Sep 23;374(6566):472–8. doi: 10.1126/science.abh2315 (PMC9302186; doi:10.1126/science.abh2315)
Supplement: Supplementary file 3 — Tables S1 to S4 [file science.abh2315_tables_s1_to_s4.zip › science.abh2315_table_captions.pdf]

Captions for Tables S1 to S4

Table S1. Receptor blocking, antibody-RBD affinity and neutralization potencies for each antibody. IC_{50} values are the average of three to six independent experiments using different virus preparations. The mean and standard deviation of triplicate measurements, when applicable, are shown for the percent blocking of ACE-2 receptor RBD binding, association and dissociation rate constants. "N.D": value not determined; "N.B": no binding or close to no binding was observed; "W.B.": extremely weak in response (< 30 RU) or in affinity (KD> 5.7 μM).

Table S2. Binary heatmap for the 186 RBD-directed antibodies. Pairwise competition results from a classical sandwich epitope binning with monomeric RBD using HT-SPR. The rows indicate immobilized mAbs and the columns represent injected analyte mAbs. Light blue cells indicate antibodies that formed a sandwiching pair and dark blue cells indicate blocking interactions.

Table S3. Negative-stain EM analysis of representative CoVIC antibodies. A total of 33 CoVIC antibodies representing different communities/clusters/bins were analyzed by NS-EM in complex with Spike trimer. Antibody formats used for NS-EM study, EMDB access numbers, 3D views of NS-maps and representative 2D classes of dominant particle populations are listed. The map for CoVIC-245 was obtained from a cryo-dataset. Models having different RBD status were fitted into NS-maps: PDB:7A94(*39*) (one RBD up); PDB:7DCX(*55*) (two RBDs up); PDB:7K4N(*19*) (three RBDs up).

Table S4. Fold-change in IC_{50} of neutralizing mAbs against pseudoviruses 458 with single mutations, relative to G614-parent virus. Values above 3 (cyan shading) and below -3 (orange shading) indicate an increase and decrease in potency, respectively. Dark red indicates a complete loss of neutralization for that virus-antibody pair.
