# Supplementary material for: Defining variant-resistant epitopes targeted by SARS-CoV-2 antibodies: A global consortium study
Source: Science. 2021 Sep 23;374(6566):472–8. doi: 10.1126/science.abh2315 (PMC9302186; doi:10.1126/science.abh2315)
Supplement: Supplementary file 4 — MDAR Reproducibility Checklist [file science.abh2315_mdar_reproducibility_checklist.pdf]

Materials Design Analysis Reporting (MDAR) Checklist for Authors

The MDAR framework establishes a minimum set of requirements in transparent reporting applicable to studies in the life sciences (see Statement of Task: doi:10.31222/osf.io/9sm4x.). The MDAR checklist is a tool for authors, editors and others seeking to adopt the MDAR framework for transparent reporting in manuscripts and other outputs.   Please refer to the MDAR Elaboration Document for additional context for the MDAR framework.

Materials

Antibodies	No (indicate where provided: page no/section/legend)	n/a
For commercial reagents, provide supplier name, catalogue number and RRID, if available.		X

Cell materials	Yes (indicate where provided: page no/section/legend)	n/a
**Cell lines:** Provide species information, strain. Provide accession number in repository **OR** supplier name, catalog number, clone number, **OR** RRID	293T, ATTC CRL-3216<br>VERO, ATTC CCL-81<br>ExpiCHO-S, ThermoFisher, #A29127	Materials and Methods
**Primary cultures:** Provide species, strain, sex of origin, genetic modification status.		X

Experimental animals	No (indicate where provided: page no/section/legend)	n/a
**Laboratory animals:** Provide species, strain, sex, age, genetic modification status. Provide accession number in repository **OR** supplier name, catalog number, clone number, **OR** RRID		X
**Animal observed in or captured from the field:** Provide species, sex and age where possible		X
**Model organisms:** Provide Accession number in repository (where relevant) **OR** RRID		X

Plants and microbes	No (indicate where provided: page no/section/legend)	n/a
**Plants:** provide species and strain, unique accession number if available, and source (including location for collected wild specimens)		X
**Microbes:** provide species and strain, unique accession number if available, and source		X

Human research participants	No (indicate where provided: page no/section/legend)	n/a
Identify authority granting ethics approval (IRB or equivalent committee(s), provide reference number for approval.		X
Provide statement confirming informed consent obtained from study participants.		X
Report on age and sex for all study participants.		X

Design

Study protocol	No (indicate where provided: page no/section/legend)	n/a
For clinical trials, provide the trial registration number **OR** cite DOI in		X

Laboratory protocol	No (indicate where provided: page no/section/legend)	n/a
Provide DOI or other citation details if detailed step-by-step protocols are		X

Experimental study design (statistics	No (indicate where provided: page no/section/legend)	n/a
State whether and how the following have been done**, or** if they were not		X
Sample size determination		X
Randomisation		X
Blinding		X
Inclusion/exclusion criteria		X

Sample definition and in-laboratory	Yes (indicate where provided: page no/section/legend)	n/a
State number of times the experiment was replicated in laboratory	**Kinetic analysis:** For each CoVIC antibody– antigen pair, the best triplicate measurements satisfying the preset data acceptance criteria were selected and the averaged ka, kd and KD values are reported. The preset acceptance criteria for quality control included 1) standard error of the estimated ka, kd and KD in each replicate ≤20% and 2) fold change for all 3 parameters within the triplicate ≤ 3.<br><br>**ACE2 blocking analysis:** The ACE2 blocking percentages shown for the CoVIC antibodies are the mean of triplicate measurements.<br><br>**Competition analysis:**<br>The majority of the clones used for the competition study were run in at least two binning runs (independent biological replicates).<br><br>**Viral neutralization assays** involving the parent virus (G614) were repeated at least two times for each antibody, with most repeated at least four times. Assays involving the viral variants are technical duplicates.	Materials and Methods, Fig. S9 legend.
Define whether data describe technical or biological replicates	Parent Virus (G614)-Antibody pairs are biological replicates while those for viral variants are techincal replicates.	

Ethics	No (indicate where provided: page no/section/legend)	n/a
Studies involving human participants: State details of authority granting ethics approval (IRB or equivalent committee(s), provide reference		X
Studies involving experimental animals: State details of authority granting ethics approval (IRB or equivalent committee(s), provide reference		X
Studies involving specimen and field samples: State if relevant permits obtained, provide details of authority approving study; if none were required,		X

Dual Use Research of Concern (DURC)	No (indicate where provided: page no/section/legend)	n/a

If study is subject to dual use research of concern, state the authority granting approval and reference number for the		

Analysis

Attrition	Yes (indicate where provided: page no/section/legend)	n/a
State if sample or data point from the analysis is excluded, and whether the criteria for exclusion were determined	Results for 9 bispecific antibodies were excluded from the competition network shown in Figure S6 and heat map in Table 2.	

Statistics	No (indicate where provided: page no/section/legend)	n/a
Describe statistical tests used and justify choice of tests.		

Data Availability	Yes (indicate where provided: page no/section/legend)	n/a
State whether newly created datasets are available, including protocols for access or restriction on access.	(EMDB and CoVIC DB (https://covicdb-submission.lji.org/summary)	
If data are publicly available, provide accession number in repository or DOI		
If publicly available data are reused, provide accession number in repository or DOI or URL, where possible.		X

Code Availability	No (indicate where provided: page no/section/legend)	n/a
For all newly generated code and software essential for replicating the		X
State whether the code or software is		X
If code is publicly available, provide accession number in repository, or DOI		X

Reporting

Adherence to community standards	Yes (indicate where provided: page no/section/legend)	n/a
MDAR framework recommends adoption of discipline-specific guidelines, established and endorsed through community initiatives. Journals have their own policy about requiring specific guidelines and recommendations to complement MDAR.		
State if relevant guidelines (eg., ICMJE, MIBBI, ARRIVE) have been followed, and whether a checklist (eg., CONSORT, PRISMA, ARRIVE) is provided with the manuscript.		X
