# Supplementary material for: Defining variant-resistant epitopes targeted by SARS-CoV-2 antibodies: A global consortium study
Source: Science. 2021 Sep 23;374(6566):472–8. doi: 10.1126/science.abh2315 (PMC9302186; doi:10.1126/science.abh2315)
Supplement: Supplementary file 3 — Tables S1 to S4 [file science.abh2315_table_s3.pdf › science.abh2315_table_s3.pdf]

Table S3. Negative-staining EM analysis of representative CoVIC antibodies.

Community	CoVIC NO.	Antibody format used for NS-EM	EMD Access NO.	EM-map Side view	EM-map Top view	Typical populations (EM 2D classes)	
RBD-1	Soluble human ACE2		EMD-24293	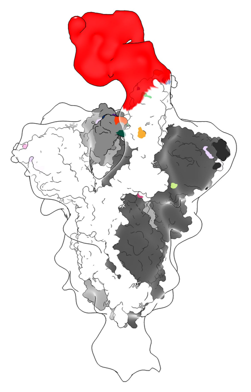	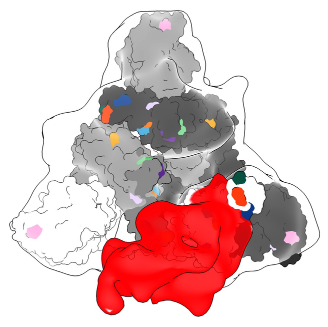	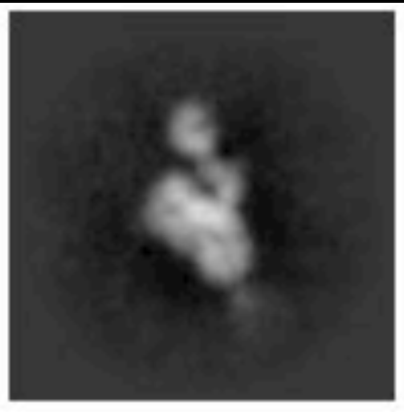	
	CoVIC-186	IgG	EMD-24336	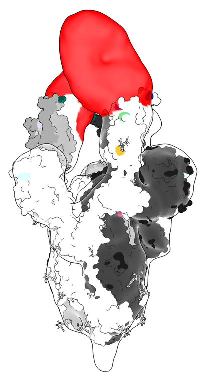	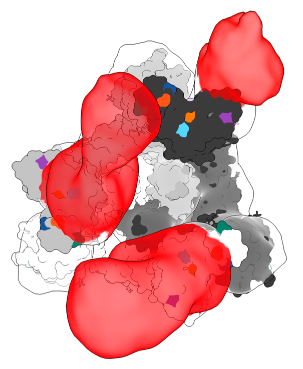	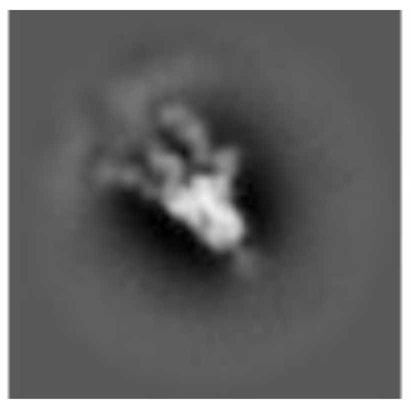	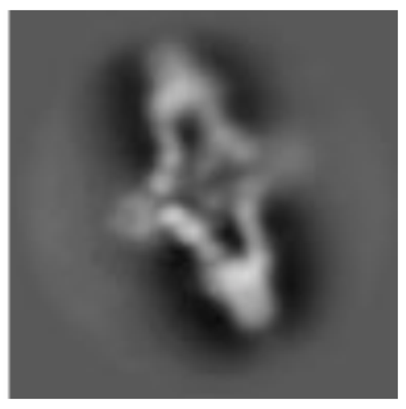
	CoVIC-259	IgG	EMD-24335	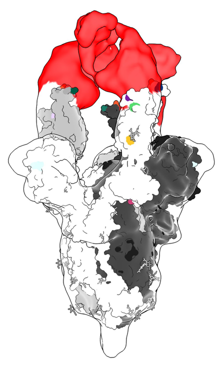	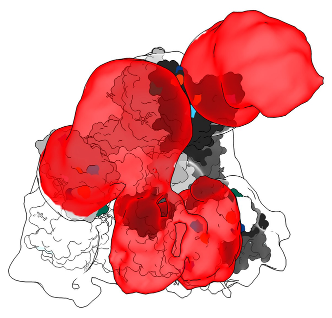	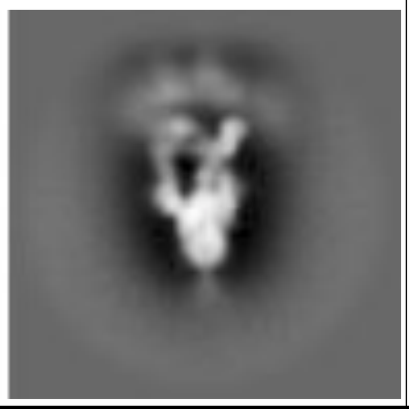	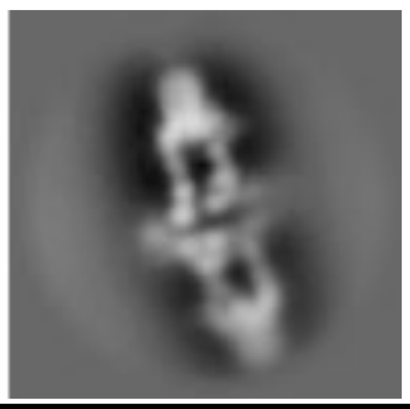
RBD-2a	CoVIC-249	IgG	EMD-24338	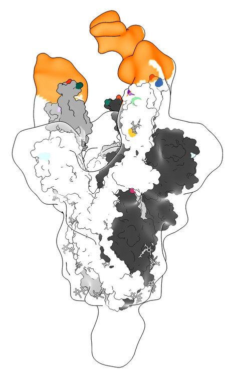	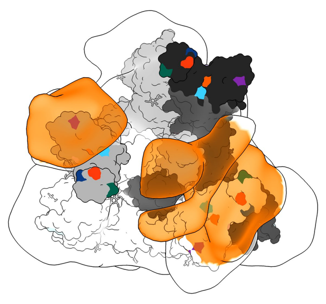	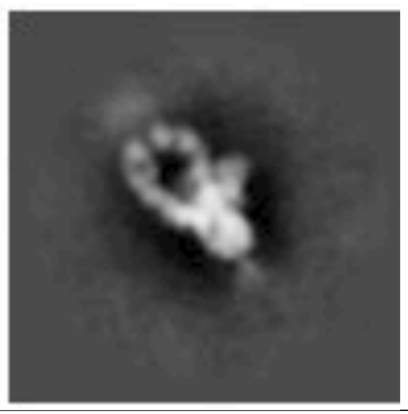	
	CoVIC-252	IgG	EMD-24339	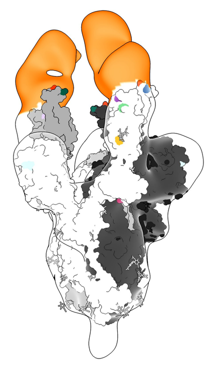	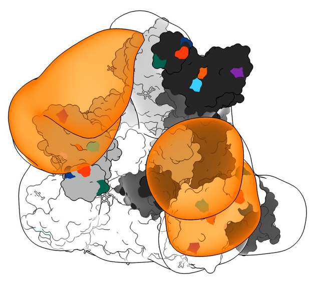	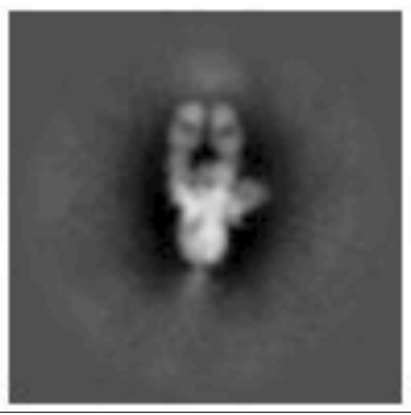	
	CoVIC-049	IgG	EMD-24340	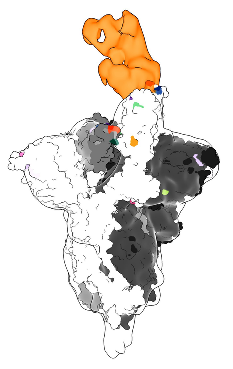	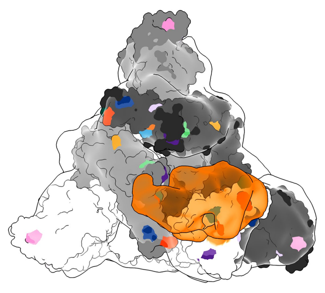	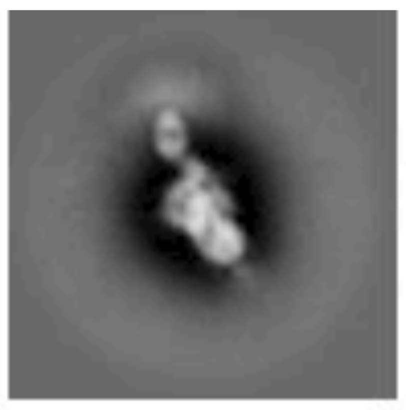	
	CoVIC-073	scFv	EMD-24341	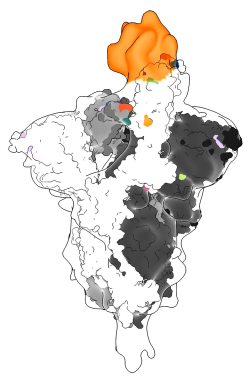	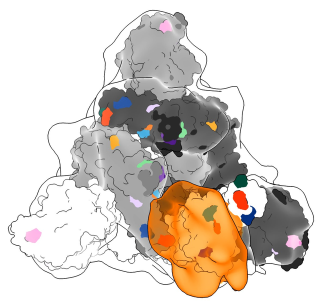	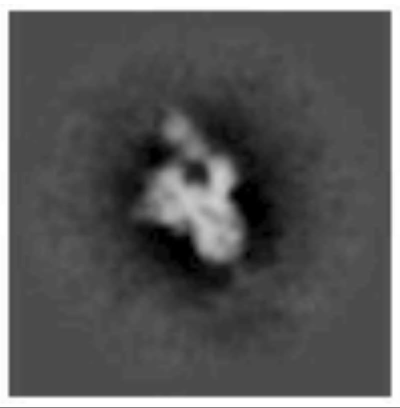	
RBD-2b.1	CoVIC-043	IgG	EMD-24342	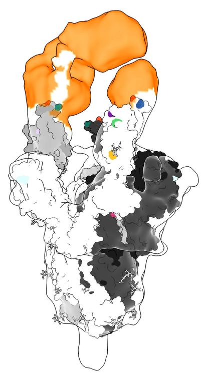	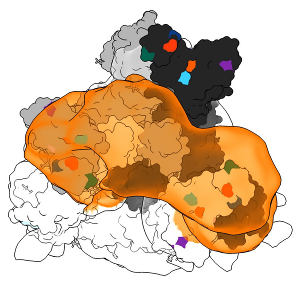	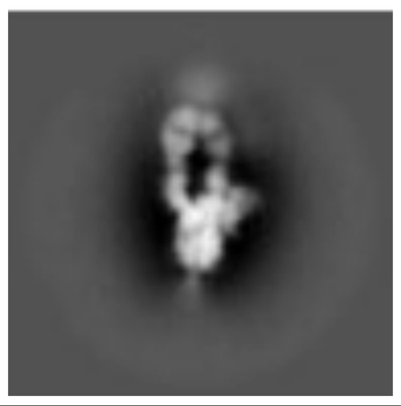	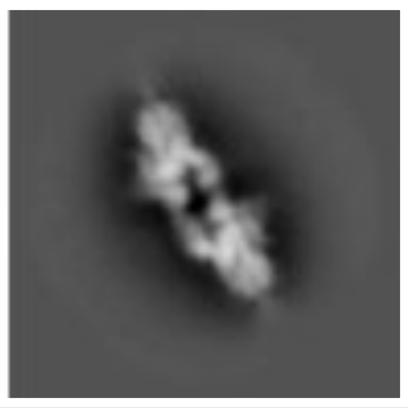
	CoVIC-010	IgG	EMD-24343	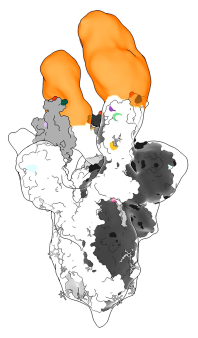	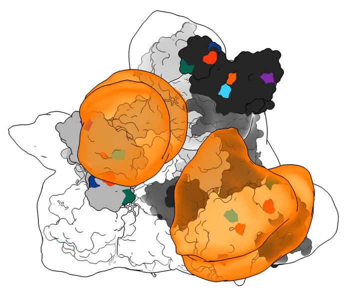	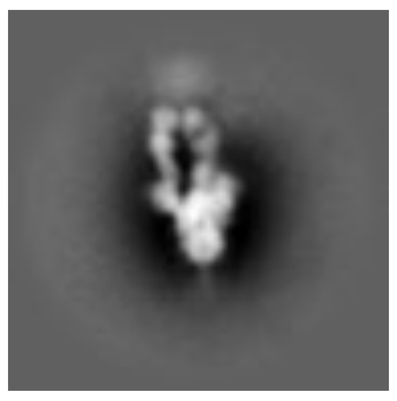	
	CoVIC-90	IgG	EMD-24357	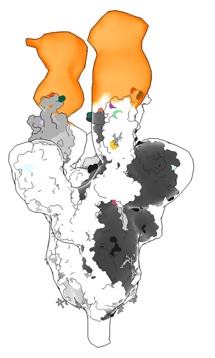	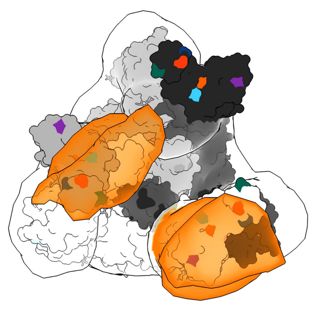	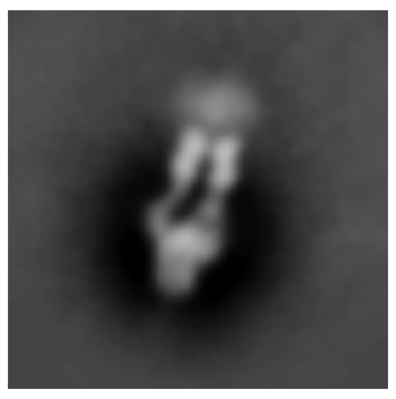	

Community	CoVIC NO.	Antibody format used for NS-EM	EMD Access NO.	EM-map Side view	EM-map Top view	Typical populations (EM 2D classes)
RBD-2b.2	CoVIC-140	IgG	EMD-24383	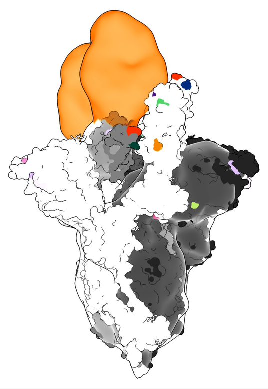	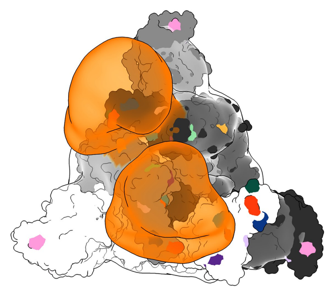	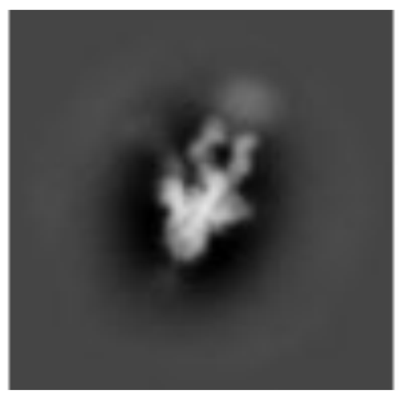 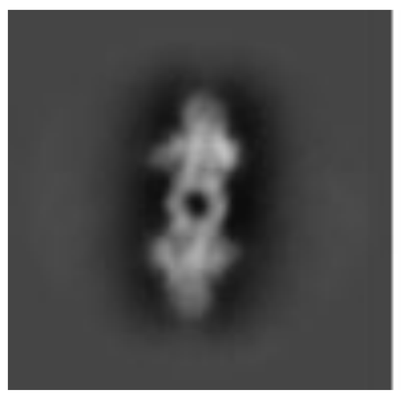
RBD-2b.2	CoVIC-148	IgG	EMD-24344	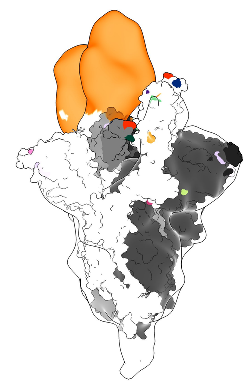	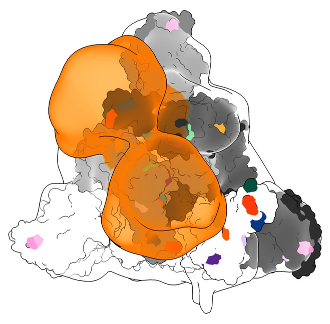	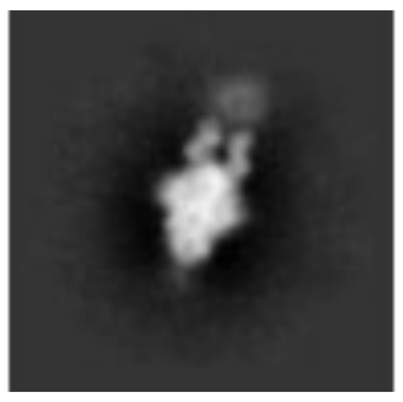
RBD-2b.3	CoVIC-002	Fab	EMD-24345	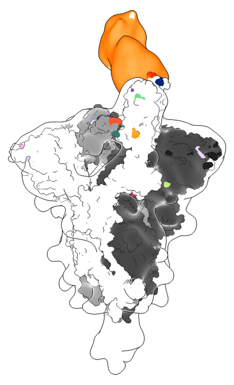	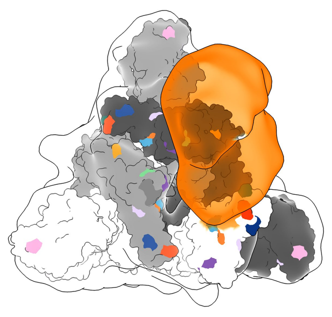	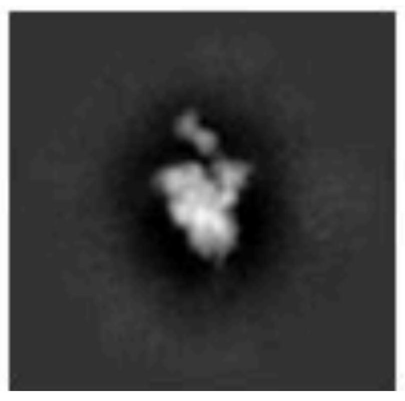
RBD-2b.3	CoVIC-147	IgG	EMD-24358	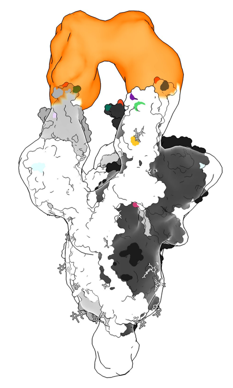	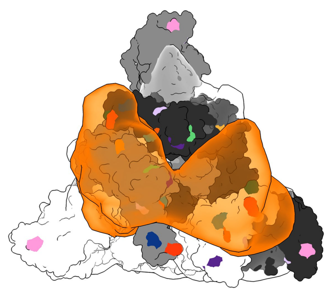	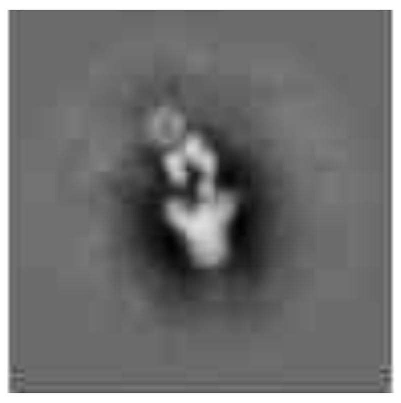
RBD-3	CoVIC-074	IgG	EMD-24361	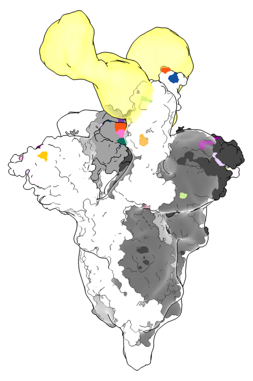	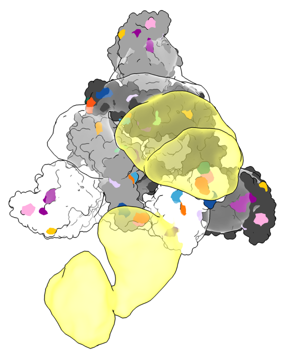	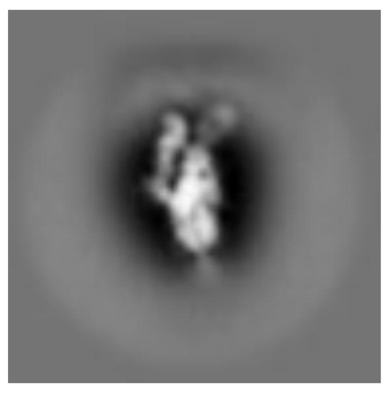 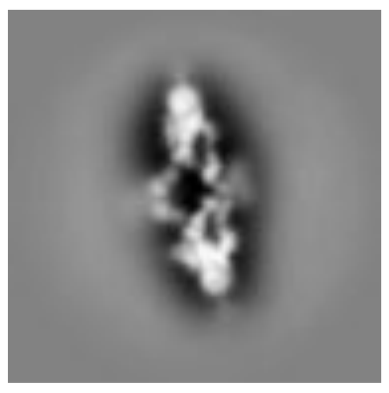
RBD-3	CoVIC-080	IgG	EMD-24346	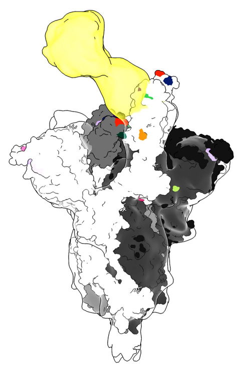	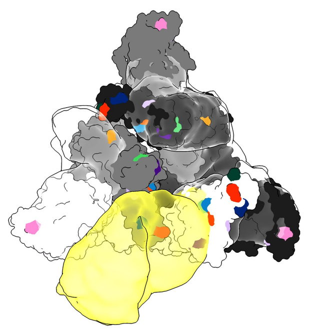	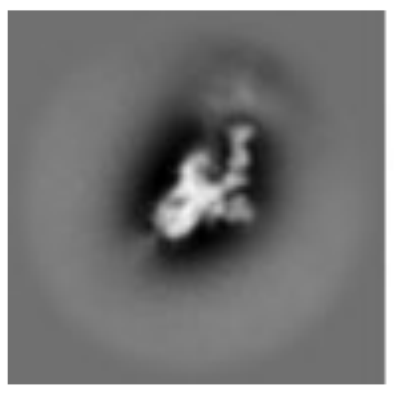 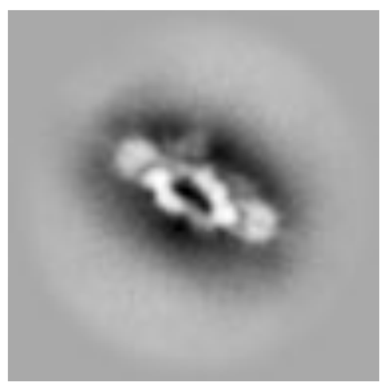
RBD-3	CoVIC-081	Fab	EMD-24348	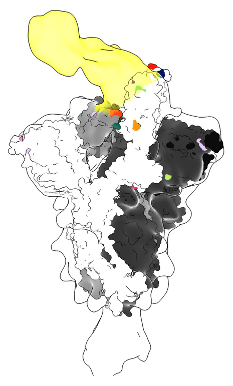	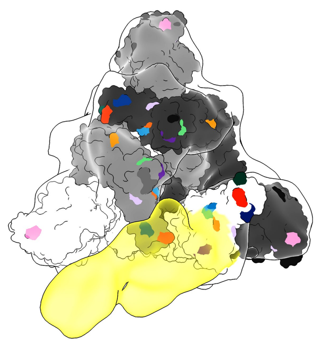	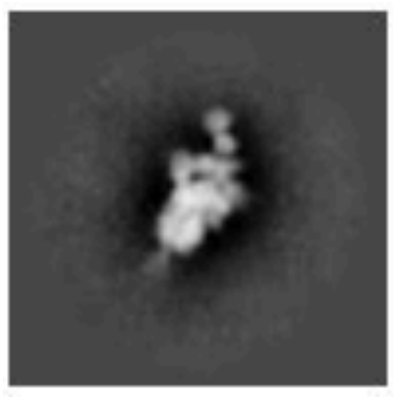
RBD-4a	CoVIC-094	VHH-Fc	EMD-24350	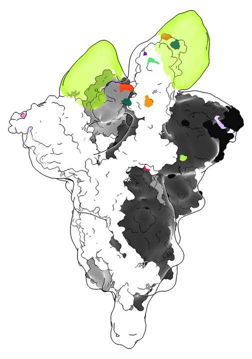	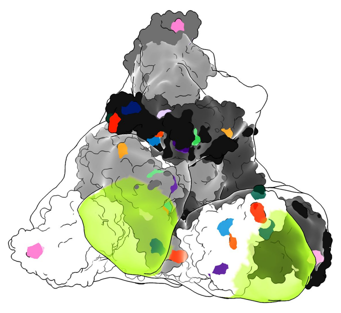	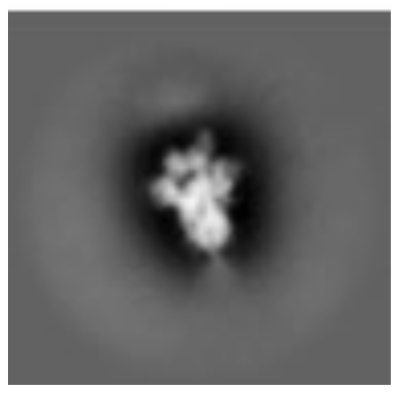
RBD-4a	CoVIC-031	IgG	N/A	N/A	N/A	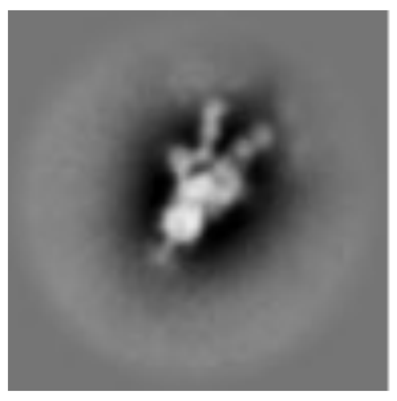 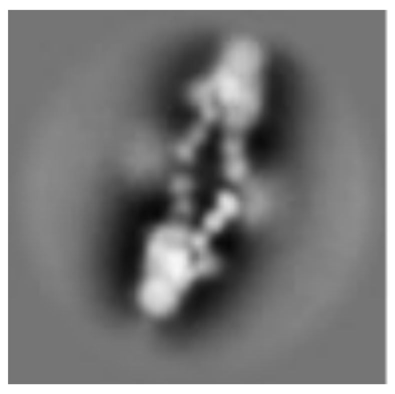
RBD-4b	CoVIC-091	IgG	EMD-24349	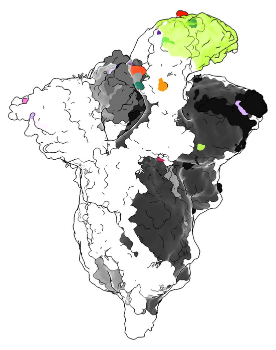	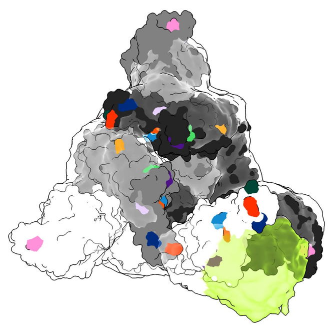	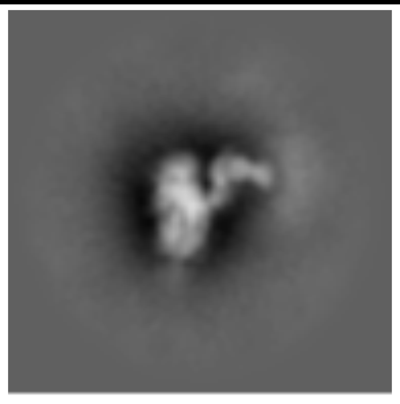

Community	CoVIC NO.	Antibody format used for NS-EM	EMD Access NO.	EM-map Side view	EM-map Top view	Typical populations (EM 2D classes)	
RBD-5a	CoVIC-134	IgG	EMD-24384	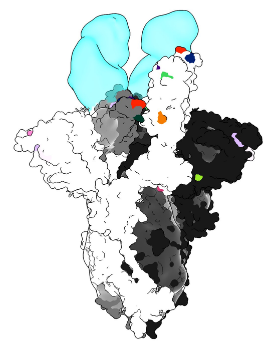	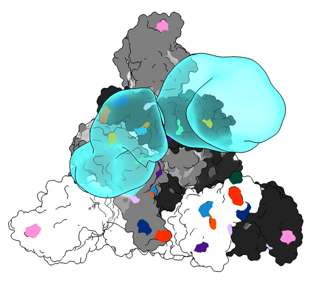	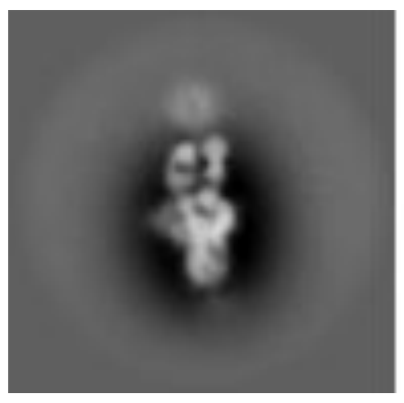	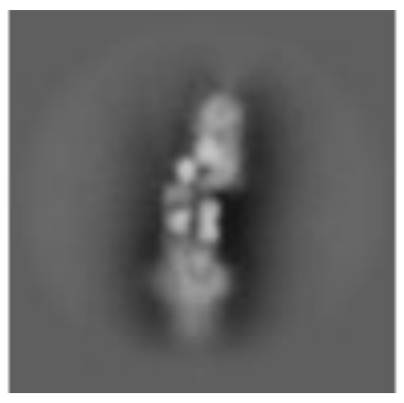
RBD-5b	CoVIC-096	IgG	EMD-24351	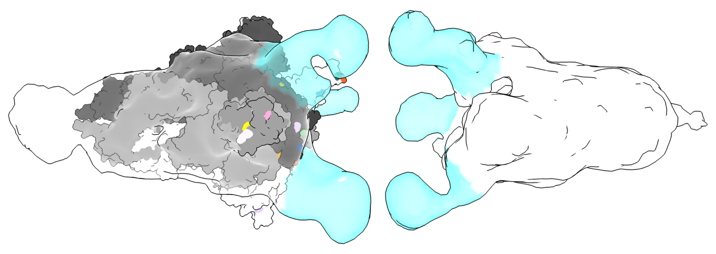			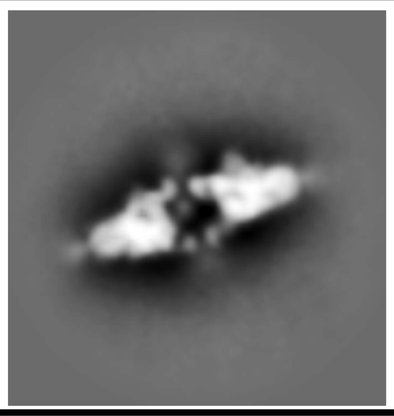
RBD-5c	CoVIC-166	IgG	N/A	N/A	N/A	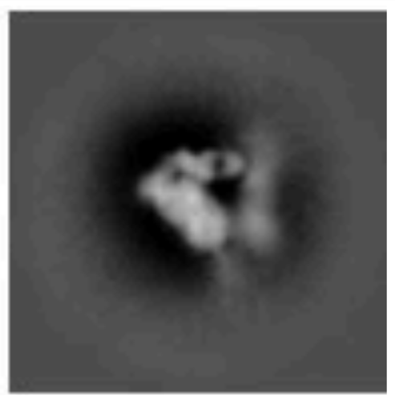	
RBD-6a	CoVIC-250	IgG	EMD-24352	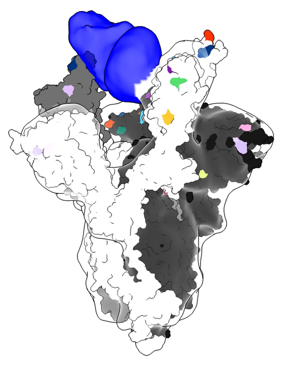	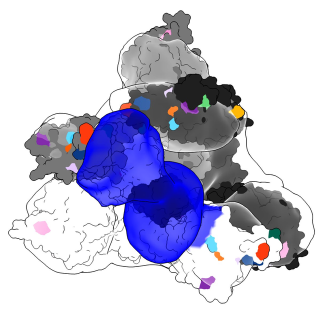	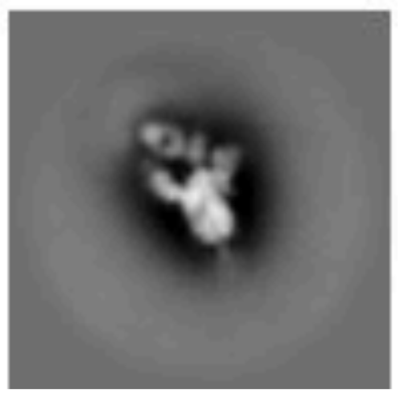	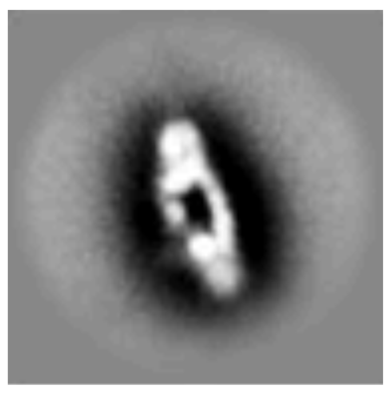
RBD-6b	CoVIC-038	IgG	EMD-24388	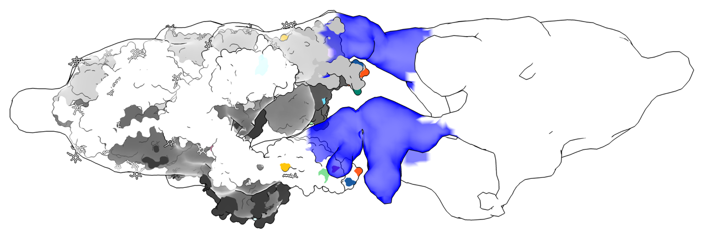			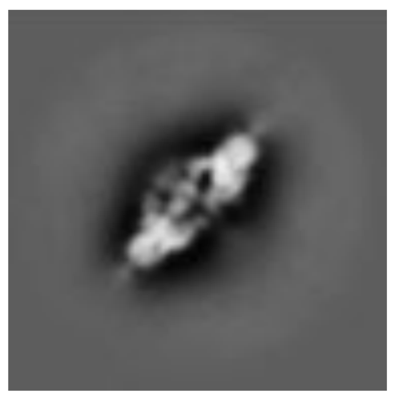
RBD-7a	CoVIC-063	VHH	EMD-24353	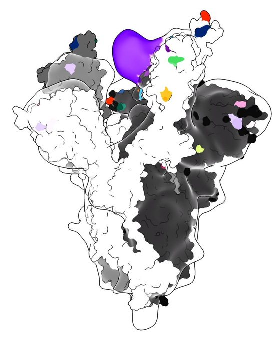	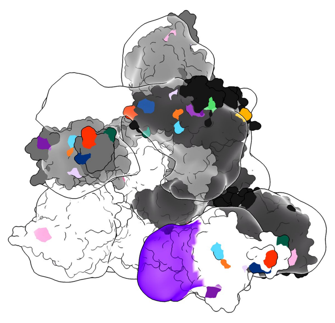	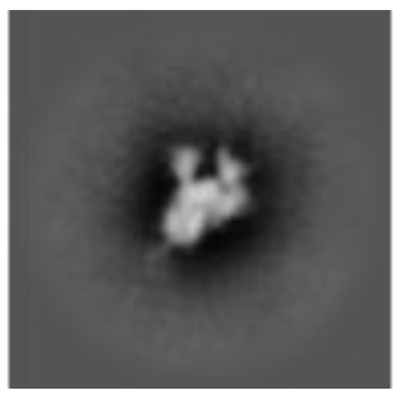	
	CoVIC-083	IgG	N/A	N/A	N/A		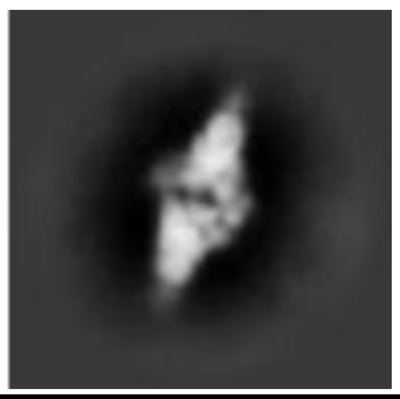
RBD-7b	CR3022	PDB: 7LOP (PMID: 34016740)					
RBD-7c	CoVIC-021	Fab	EMD-24354	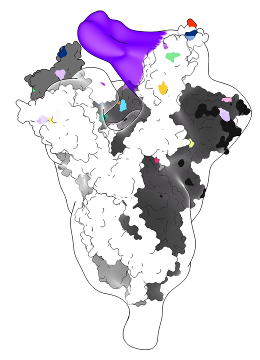	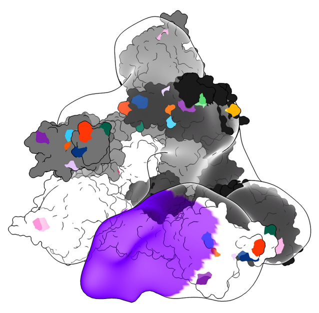	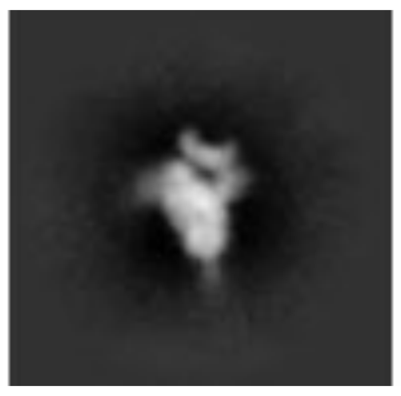	

Community	CoVIC NO.	Antibody format used for NS-EM	EMD Access NO.	EM-map Side view	EM-map Top view	Typical populations (EM 2D classes)	
NTD-1	CoVIC-246	IgG	EMD-24359	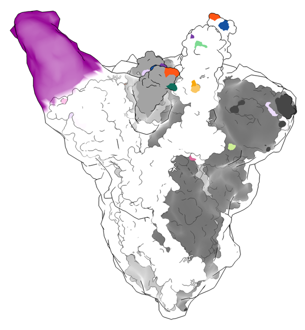	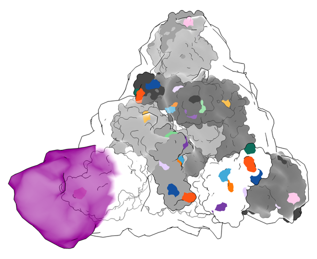	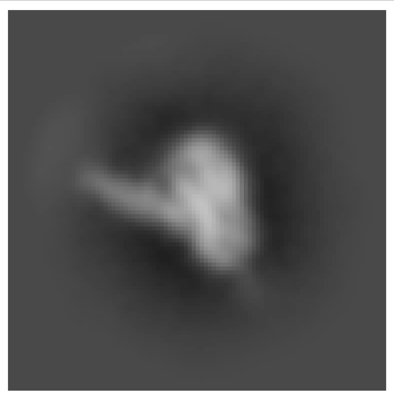	
	CoVIC-247	IgG	EMD-24355	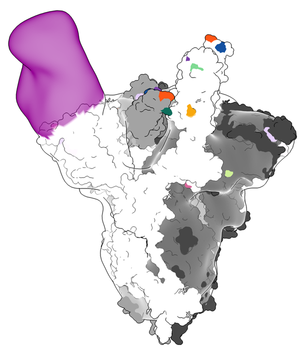	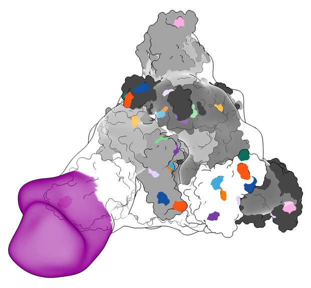	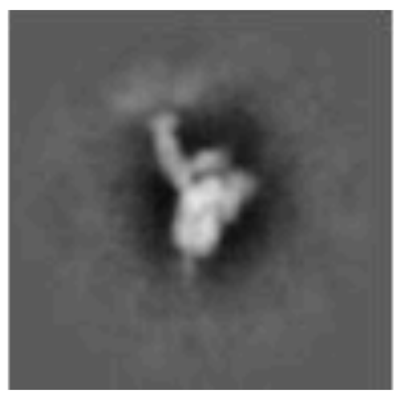	
NTD-2	CoVIC-245	IgG	EMD-24360	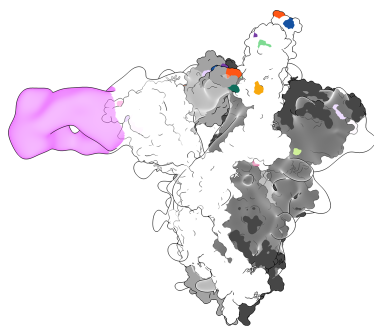	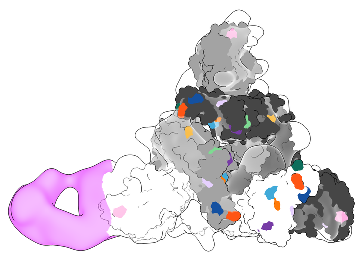	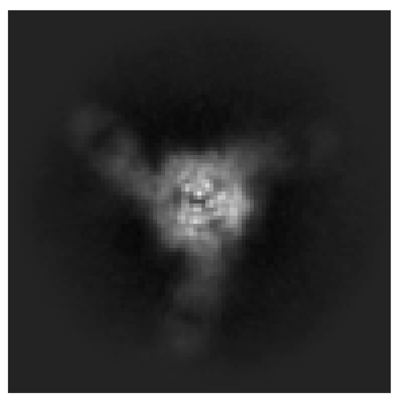	
NTD-3	CoVIC-020	Fab	EMD-24356	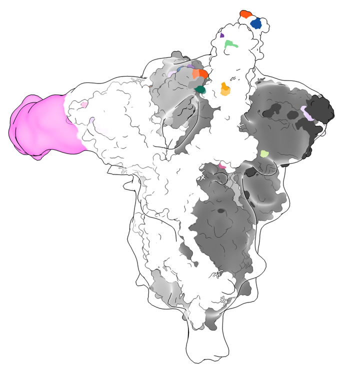	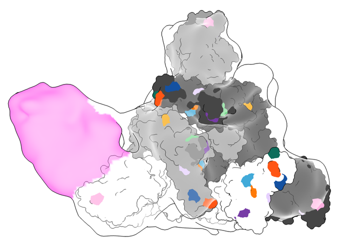	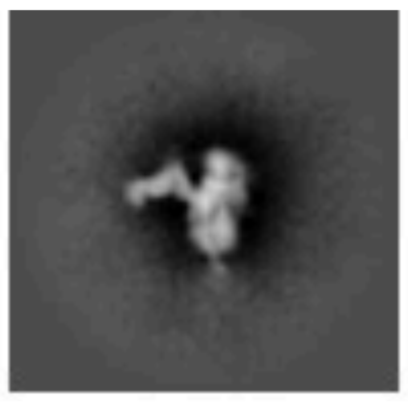	
RBD-1/NTD-1	CoVIC-199	Bi-specific IgG	EMD-24337	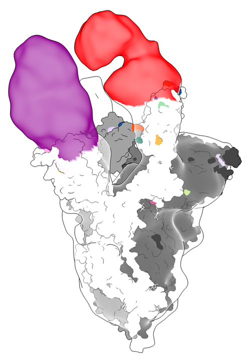	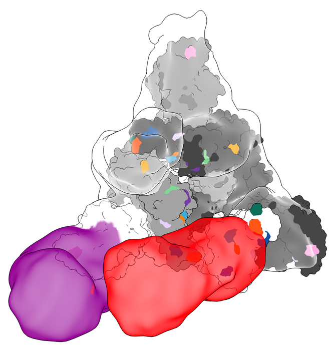	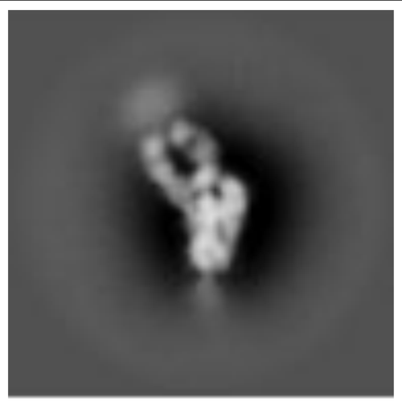	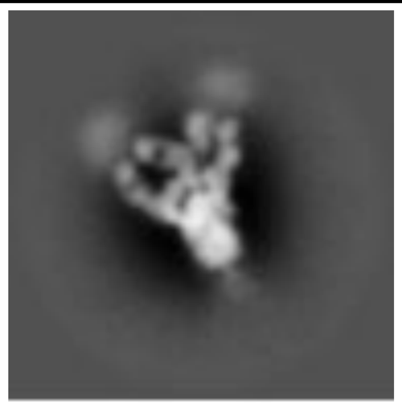
